# Current and Near-Term Earth-Observing Environmental Satellites, Their Missions, Characteristics, Instruments, and Applications

**DOI:** 10.3390/s24113488

**Published:** 2024-05-28

**Authors:** Susan L. Ustin, Elizabeth McPhee Middleton

**Affiliations:** 1 Institute of the Environment, University of California, Davis, Davis, CA 95616, USA; 2 National Aeronautics and Space Administration, Greenbelt, MD 20771, USA; betsymail@comcast.net

**Keywords:** earth-observing satellites, space-based environmental satellites, pioneering satellites, active and passive instruments, imaging spectrometers, multispectral imagers, radar and microwave instruments, current satellites, climate change, planned satellites into the 2030s

## Abstract

Among the essential tools to address global environmental information requirements are the Earth-Observing (EO) satellites with free and open data access. This paper reviews those EO satellites from international space programs that already, or will in the next decade or so, provide essential data of importance to the environmental sciences that describe Earth’s status. We summarize factors distinguishing those pioneering satellites placed in space over the past half century, and their links to modern ones, and the changing priorities for spaceborne instruments and platforms. We illustrate the broad sweep of instrument technologies useful for observing different aspects of the physio-biological aspects of the Earth’s surface, spanning wavelengths from the UV-A at 380 nanometers to microwave and radar out to 1 m. We provide a background on the technical specifications of each mission and its primary instrument(s), the types of data collected, and examples of applications that illustrate these observations. We provide websites for additional mission details of each instrument, the history or context behind their measurements, and additional details about their instrument design, specifications, and measurements.

## 1. Introduction

Space-based Earth imaging began with primitive and somewhat blurry images as seen from the first Landsat Multispectral Scanner images acquired over a half century ago (in 1972) that nevertheless changed the way we think about the Earth. Those first coarse-scale images acquired across a disparate collection of landscapes reminded us that we live on an amazing sphere in space and stimulated the ensuing recognition of our need to sustainably manage our planet and its resources. Over the past 50 years, various Earth-Observing (EO) satellites have provided ever more important new sources of information about the land, oceans, ice, and atmosphere of our Earth. And new remote sensing technologies from the last decade or so have provided transformational satellite capabilities, and even more sophisticated instruments are forthcoming over the next decade and beyond.

Three years ago, Ustin and Middleton in 2021 [1] summarized and described basic characteristics of EO satellites that are, or have been, in orbit over the past five decades, and some of those expected to be in orbit over the coming decade that provide data products for ecological and environmental research communities, as well as information for environmental resource managers and policy decision makers. While widely viewed, the information provided in Ustin and Middleton [1] was a static view of the international array of EO satellites, from the perspective in 2020. The quality and sophistication of instruments employed for space-based observations, and the science information derived from them, are rapidly improving, facilitated by coincident exceptional advances in computational and geospatial technologies. Consequently, rapid advances in the ability to address Earth Science topics and practical applications are yielding new societal benefits.

This paper updates the status of current, ongoing environmental satellites and those planned for launch later this decade and into the early 2030s, in context with early space-based pioneers. Satellite programs require enormous investments at all stages, from concept development through operational implementation and generation of products for distribution to interested parties. We summarize the science objectives of current and planned EO satellites of the [2,3] major participating space agencies, adding significant new information on aspects not previously covered, and updates on changes and expansion of information that has recently become available. We include instrument summaries for space-based EO observations, incorporating information on improved measurements that are required to achieve advanced scientific objectives, as well as reporting on changing mission priorities and altered launch schedules for those in the pipeline.

### 1.1. International Recognition and Cooperation towards Documenting Planet Earth

As we approach the middle of the 21st century, there is a critical need to establish the baseline state of our planet, in terms of the actual amount, distribution, and health status of ecosystems worldwide, and to document the impacts imposed by humankind. There is increasing acceptance that Planet Earth is experiencing many simultaneous environmental and societal challenges, which are often occurring at accelerating rates [2,3,4] that necessitates more sophisticated and innovative space-based approaches to acquire the science-quality synoptic information, especially imagery-based information, necessary to accurately describe, evaluate, and manage the surface resources of our home planet, while global population levels increase and biological species are being lost at alarming rates [5,6,7]. Today, only approximately 17% of the land and 10% of the oceans are legally protected from biodiversity degradation. The strategic goal #11 of the 2011 Aichi Targets was to bring the percent of terrestrial and inland water to 17% conserved and 10% marine, with well-connected pathways to maximize their integrity and functioning [8]. While it is likely that this goal may have been met, the results indicate the need for more aggressive conservation in areas of high biodiversity concern since further protection of areas with low biodiversity does not advance the strategic goals. The global impacts of the mismanagement of ecosystems and their essential provisioning services for the Earth’s people is increasingly apparent [5] and of increasing concern as to whether we will or have passed a global “tipping point(s)” [9,10]. The 2022 United Nations’ Convention on Biological Diversity, recognizing accelerating biodiversity losses across the globe, approved an international agreement to protect 30% of Earth’s lands and oceans by 2030 (the “30 × 30” Initiative), which was signed by representatives of 188 countries of the 196 participants (www.unep.org/news-and-stories/story/cop15-ends-landmark-biodiversity-agreement/ (accessed on 23 November 2023).

### 1.2. The Rationale for More Sophisticated Space-Based Surveillance of Our Earth System

The urgency and cost burden of acquiring consistent global-scale data have forged stronger international space agency cooperation and commitment to support the technology developments necessary to measure, manage, and interpret the unprecedented large data volumes, and the software analysis tools necessary to produce user-friendly data products. In 2020, for example, the EO portfolio accumulated by the USA’s National Aeronautics and Space Agency (NASA) was on the order of 100 Terabytes/day (https://sealevel.nasa.gov/news/226/nasa-turns-to-the-cloud-for-help-with-next-generation-earth-missions/ accessed on 23 November 2023), and similar large data volumes were collected by other international space agencies such as the European Space Agency (ESA), Paris, France and the Japan Aerospace Exploration Agency (JAXA), Tokyo, Japan. New satellites in development will multiply these numbers by orders of magnitude, both because of more satellites in orbit hosting multiple instruments, but also because new data collections will have higher spectral, spatial, and temporal resolutions (and other properties); these factors taken together will yield tremendously larger collections. For example, it is estimated that climate change-related data will increase NASA’s EO archive to an estimated 230 Petabytes by 2030 (https://www.codingninjas.com/codestudio/library/nasa/ (accessed on 3 April 2024)). This impressive data volume represents a relatively small fraction of the entire space-based collections compiled by various USA and international space agencies and by other nations.

It is enlightening to note that seventy-eight international organizations have sponsored satellites since 1974, as summarized in 2022 by OSCAR (Observing Systems Capability Analysis and Review Tool (https:/space.oscar.wmo.int/ (accessed on 12 May 2024)). The most active programs have been sponsored by the USA (NASA and the National Oceanic and Atmospheric Administration, NOAA), Europe (ESA and the European Organization for Exploitation of Meteorological Satellites, EUMETSAT), China, France, India, and Japan. Satellites to collect EO information have been sponsored by space agencies and other governmental departments plus commercial enterprises in a host of countries, including Algeria, Argentina, Brazil, Canada, China, France, Germany, India, Italy, Japan, Korea, Peru, Spain, the Russian Federation, and the UK. However, there have been few efforts to combine and/or compare data and imagery among these already existing satellite-acquired data including imagery to address regional or global patterns and trends to establish a climate change baseline. In 2024 we are well into an era of rapid environmental changes and yet this global data remains unsynthesized and largely ignored, while the ability to construct a baseline from future data is becoming impossible. This is an environmental project of utmost importance and urgency.

In recent years, there has been a growing interest in explicit descriptions of our planet’s changing surface to delineate landscapes at local rather than synoptic scales yet embedded within global surveys. The rationale for more sophisticated space-based surveillance of our Earth System at these local scales is tied to the urgent need to address the many interlocking science and societal challenges, including issues such as (i) monitoring agricultural status for food security and sustainability [11,12], reducing agricultural chemical and water inputs, with less available arable land and more people to feed [13]; (ii) identifying ecosystem biodiversity hotspots and areas of biodiversity degradation, quantifying losses and gains, and prioritizing restoration targets [14,15]; (iii) determining the physical properties of land surfaces, such as forests and rangelands, urban and developed landscapes, and snow and ice cover [16,17], and their effects on weather systems and climate [18,19]; (iv) surveying surface water quality and quantity [20,21] and impacts of increasing eutrophication and salinization [22,23]; (v) reducing the errors in global models that estimate land–ocean–atmosphere processes and biogeochemical cycles, including biosphere interactions linked through assimilation, respiration and [18] evapotranspiration [24,25]; (vi) developing workable protocols for establishing and sustaining more favorable microclimates in cities, such as with urban forests and parks to moderate excessive heat and air pollution [26,27]; sea-surface temperatures, sea ice extent/concentration [28,29,30]; sea-surface salinity [31,32]; and (vii) tracking hazard impacts, such as flashfloods, landslides, airborne volcanic debris and extreme weather-related events such as wildfires, sandstorms, hurricanes and tornadoes [33,34,35,36,37,38].

Advances are being made to develop mitigating strategies that improve these problem areas with existing data, but ongoing monitoring is essential. In addition, upgraded capabilities to acquire more sophisticated space-based data, such as imaging spectroscopy, will benefit from different combinations of temporal frequency, spatial resolution and enhanced information content. For space-based imagers, this is being addressed with additional and narrower spectral bands for precise targeting/discrimination, and by expanding into other wavelength/frequency regions beyond the visible/near-infrared (VNIR) wavelengths, such as shortwave infrared (SWIR), thermal infrared (TIR), microwave and radar—also with better waveband and spatial resolutions. Another urgent need is for such data to be measurable in physical energy units (e.g., g/m^2^) so they can be compared across regions, time periods, and instruments, and input into process-based models at all scales, such as carbon cycle simulations and projections for climate changes.

While the whole-Earth surveillance approach is necessary at higher spatial and temporal scales to better understand the linkages among the many biogeochemical processes on land, sea, and atmosphere, this necessarily yields unprecedented amounts of globally distributed data. Therefore, data management protocols are necessary. These include the development of improved methods for managing: (i) the processes related to data retrieval and archiving; (ii) the computationally efficient tools to locate and extract specific geographic and temporal data; and (iii) the analytical tools that enable efficient visualization and utilization of these data. In addition, free and open international access to the satellite data archives must be ensured. If we fail to provide the funding, training and access to digital resources that enable global utilization of these data, we will lose a critical resource in developing better understanding about spatial and temporal changes over the next decades.

### 1.3. Materials and Methods

All of the technical information in this paper was obtained from public websites and publications by searching Google Scholar and Web of Science, mission websites, searching “OSCAR” the World Meteorological Organization database of existing satellites, searching NASA (eospo.nasa.gov/mission-category/ (accessed on 15 May 2024)), USGS (https://www.usgs.gov/centers/eros/data/ and https://lpdaac.usgs.gov/ (accessed on 15 May 2023)), NOAA (https://www.noaa.gov/satellites/ (accessed on 15 May 2024)), Copernicus (https://www.copernicus.eu/en/ (accessed on 15 May 2024)), EUMETSAT (https://www.eumetsat.int/ (accessed on 15 May 2024)), the EO Portal (https://www.eoportal.org/ accessed on 15 May 2024)), search specific satellites or instruments, and Wikipedia (see individual missions). Often there are multiple versions describing some instruments online at different phases of fabrication, and initially reported characteristics of a satellite or its instruments can change before and during operation, including what orbital altitude, what equatorial crossing time, and what bands and band passes it will have. The published information is not always in agreement, and web pages generally lack dates of when they were released. Sometimes differences among sources are small, e.g., a few nm in a wavelength position, but often information differs significantly among sources. We have tried to present the most current and correct information available.

All information from a satellite’s flight characteristics (e.g., inclination angle, crossing time) and its instruments’ science objectives were obtained from the web or from information publicly available as reported by mission spokespersons. We scoured the websites of NASA, USGS, NOAA, the European Union’s Copernicus program and all of its sub-programs, EUMETSAT, the Committee on Earth Observation Satellites (CEOS), and governmental space agencies in many individual countries, including Canada, France, Germany, and Italy. Often a single instrument is described in multiple websites (some with conflicting information) such as for Germany’s EnMAP satellite program (https://www.enmap.org/ (accessed on 15 May 2024)) or from the Earth Observation Center for the German Space Agency (DLR, Deutsches Zentum fűr Luft-und Raumfahrt) (https://www.dlr.de/eoc/en/desktopdefault.aspx/tabid-5514/20470_read-47899/ (accessed on 15 May 2024)), or for NASA’s missions such as the ECOSTRESS program site (https://ecostress.jpl.nasa.gov/ (accesssed 15 May 2024)). The major agencies provide mission information, such as NASA’s Earth System Observatory (https://science.nasa.gov/earth-science/earth-system-observatory/ (accessed on 15 May 2024)); NOAA provides information on its geostationary and polar-orbiting satellites, as does the U.S. Geological Survey (USGS) for the Landsat program (https://www.usgs.gov/landsat-missions). ESA summarizes information for the Copernicus program (https://www.copernicus.eu/en/ (accessed on 15 May 2024)), and the Copernicus Open Access Hub (https://scihub.copernicus.eu/ (accessed on 15 May 2024)), and ESA’s CEOS EO Database (https://database.eohandbook.com/ (accessed on 15 May 2024)). In addition, useful information is summarized by the World Meteorological Organization’s (WHO) Observing Systems Capability Analysis and Review Tool (OSCAR, https://space.oscar.wmo.int/ (accessed on 15 May 2024)), and the lead programs fabricating them, and their associated science team(s). We looked up the websites for each of the instruments described, e.g., the Sentinel Hub (https://www.sentinel-hub.com/explore/ (accessed on 15 May 2024)) or as in the NASA-ISRO NISAR mission (https://www.jpl.nasa.gov/missions/nasa-isro-synthetic-aperture-radar-nisar/ (accessed on 15 May 2024)) and from various public data sources like Gunter’s Space Page (https://space.skyrocket.de/directories/sat.htm/ (accessed on 15 May 2024) and the Union of Concerned Scientists Satellite Database (https://www.ucsusa.org/resources/satellite-database/ (accessed on 15 May 2024)). We have tried to acknowledge data sources throughout this paper but accept responsibility for our misinterpretations and errors.

## 2. Background: EO Satellites

There are numerous satellites circling the Earth, at all altitudes and for many purposes, to support communications, military operations, science research, and to monitor atmospheric, land and surface water processes for the benefit of society (e.g., management of resources). We have limited our attention here to those satellites designed to collect information about the Earth System that can expand our knowledge about the health of our planet. We also place emphasis on those satellite instruments that passively acquire spectral and thermal images of the Earth–atmosphere system, referred to as “imagers”, but we also survey other types with critical roles, such as active systems measuring lidar, radar, and microwave soundings.

Widespread access to existing remote sensing data has provided researchers the ability to address many new science questions, such as those that require studies of large-scale geographic areas or time series collected over several decades, e.g., since ~1980. In addition, the increases in both spectral and spatial resolutions (i.e., finer bands and smaller pixels) in more recent space-based imagers have enabled more refined interpretations of land cover composition and biogeochemical variables. Other technology advances have facilitated the adoption of remote sensing information, such as advances in GPS technologies that improve both satellite pointing accuracy and geolocation of ground reference coordinates, advances in computer science capacity to handle large data arrays, and the development of sophisticated statistical software packages for analyzing and interpreting these data. Consequently, the availability of calibrated and geolocated space-based “science-quality” data has fundamentally changed how Earth Sciences research is conducted and interpreted [39].

**Why are so many space-based observations needed?** Spaceborne observations are essential to provide synoptic and repeated observations that accurately and consistently quantify the Earth’s current conditions, and to monitor changes. However, spaceborne instruments have a limited lifetime, generally 3–7 years on orbit (although a very few have been operating for 20+ years), requiring replacements at regular intervals. Also, new satellites replace older ones when technology upgrades become possible. 

Satellites carry instruments designed specifically to collect either passively available information or to actively send/receive energy pulses, referred to as “passive” and “active” systems, respectively, or to provide various communication functions. We place emphasis on passive imagers, which are designed to acquire environmental information (e.g., radiance, reflectance) over specified wavelengths in the solar energy regimes: ultraviolet (UV), visible/near-infrared (VIS/NIR or VNIR), shortwave infrared (SWIR), visible through SWIR (VSWIR), and thermal infrared (TIR) wavelengths. We also include active systems, which include radar, lidar, and microwave soundings, from which energy pulses sent/received provide profile information.

Recently, developments in technology have gone far beyond thematic categorial representations of our fragile world and support the requirements for greater scientific rigor. Thus, new missions are striving to acquire more accurate physical measurements by obtaining more frequent views, and/or additional or finer-resolution spectral bands for improved discrimination of various atmospheric constituents (e.g., chemical components and particles) or surface properties, including the spectral identification of plant species, soil types, and geologic minerals. Furthermore, accurate determination of the surface properties requires accurate representation and removal of the overlying atmosphere’s spectral distortions, referred to as “atmospheric correction”.

In addition to carrying different payloads, satellites acquire data collected over a wide range of spatial and temporal resolutions, depending on the specific requirements of the on-board instruments. A satellite’s forward line of direction is its “path” or track, and each observation is associated with a defined footprint or “pixel”. In addition to pixels viewed straight down (referred to as the nadir view, 0°) along its central path, adjacent pixels are also viewed along its “swath”, the area viewed perpendicular to its path, side-to-side. Satellite instruments that acquire frequent (e.g., near-daily), global observations necessarily have large footprints (250 m–4 km); this is possible with varying oblique viewing geometry (e.g., ≤60°) across wide swaths of the atmosphere–surface system. In contrast, satellites that view the surface with narrow swaths acquire local information at scales ≤ 50 m with constrained viewing geometry (e.g., nadir, 0° ± 20°) but require weeks to return to the same location, so views are infrequent.

### 2.1. Primary Types of Orbiting Instruments

The type of technology used in a satellite determines what information is expected to be obtained and how the data will be used. The earliest satellites measured one or a few bands in the visible (400–700 nm) and/or in the near-infrared (700–1500 nm) wavelength region of the electromagnetic spectrum. As technologies evolved, instruments began to include wavelengths in the thermal infrared in the 8–13 μm region, then shortwave infrared (1500–2500 nm), and the midwave infrared (3–6 μm). Active instruments such as radar and microwave collect data in longwave bands (Ka, K, Ku band wavelengths: 0.3–2.4 cm wavelength, ~40–18 GHz), or out to P-band radars (0.3–1.3 m, ~0.216–0.45 GHz). Lidar technology has been a recent addition to space because the lasers that generate the energy pulse have been quick to burn out, thus not justifying their cost. However, today, this concern is rapidly changing and lidar data are becoming available for space research.

#### 2.1.1. Passive Imagers

Passive imaging instruments, from which images are constructed (looking like photographs), record the reflected sunlight from the Earth’s surface, including reflectance from the intervening atmosphere, are of three general types: (1) multispectral systems, (2) hyperspectral imagers and (3) imaging spectrometers. Multispectral systems are comprised of a specific set of a few (<10) relatively wide (≥20 nm) spectral bands, usually non-contiguous, and often limited to the VIS/NIR region (e.g., Landsat’s 4–7). Hyperspectral systems have many more (15–25) usually narrower (10–20 nm) spectral bands, some of which may be contiguous, and may span the UV to SWIR regions (e.g., Visible Infrared Imaging Radiometer Suite (VIIRS). Imaging spectrometers (ISs) often have hundreds of overlapping, narrow (≤15 nm) spectral bands, covering the VIS to SWIR regions, such as Germany’s new Environmental Mapping and Analysis Program, EnMAP [40] and aircraft instruments such as AVIRIS [37,41,42,43]. Some spectrometers in space have very narrow bands (≤1 nm) covering a selected wavelength region, but are not imagers (typically sounders), that have thus far mostly applied to identifying atmospheric constituents, e.g., Global Ozone Monitoring Experiment, GOME [44].

**Figure 1:**
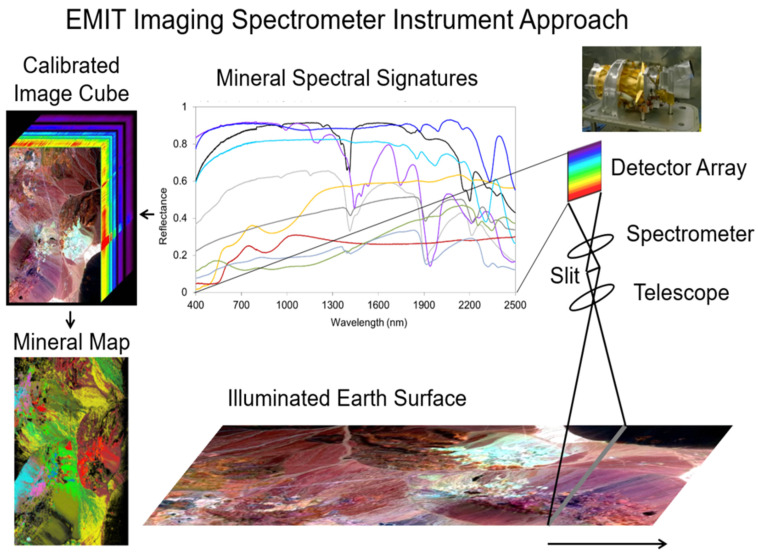
This illustration shows how a spectrometer works, from scanning across a segment of land, to capturing the reflected energy, here in a Dyson telescope-spectrometer system that focuses the collected light on a diffraction grating with a structured blaze that then sends the light into a new three-zone order sorting filter (which allows measuring the entire spectrum with one spectrometer, without noise from second order light). The detector is mercury cadmium telluride that is sensitive over the full spectrum [45]. An image is created from the forward motion of the satellite (or aircraft) and the data are organized in a data cube, one layer for each band. There are 1240 pixels across the 60 m swath. The bands are ordered sequentially, from the shortest wavelength (380 nm) to the longest (2500 nm) for all pixels, visualized across the top and the right side of the figure. Three bands are chosen to make a false color image for the surface of the data cube. The spectrum for each pixel is shown across the top and along the right side, with colors indicating the intensity of the reflectance. Band colors are ordered with highest reflectance values colored red, next is yellow, and colors follow the rainbow to dark blue-purple-black indicating the lowest reflectance values. The different patterns seen in the spectra are different surface materials. The middle of the panel shows reference spectra for typical minerals from arid regions that might be present in the data cube. The analysis of the data cube using spectral signatures, such as those in the graph, produces a map of the area; in this example, a mineral map. Credit: EMIT Overview. EMIT Earth Surface Material Dust Source investigation: Earth.jpl.nasa.gov/emit/ (accessed on 5 January 2024).

With repeated observations, a spectral data cube for a geographic area can be built, where the latitude and longitude provide the X and Y axes and each of the measured wavelength bands provide the 3rd axis, forming the basis of satellite geographic information systems. An example is shown in Figure 1, illustrating the processing stages for the EMIT instrument (Refer to Section 9 below).

As with this example, “full-spectrum ISs” typically measure a complete visible (V or VIS) to shortwave (SWIR) spectrum (VSWIR), between ~380–400 nm and 2500 nm in the solar reflective wavelengths reaching the Earth’s surface, with between 200 and 500 spectral bands, depending on the IS’s spectral resolution. This advanced technology, known as imaging spectroscopy, furnishes sufficiently high spectral resolution across the reflected spectrum to enable identification of specific materials based on their characteristic biogeochemistry and composition, which is facilitated by having contiguous (no wavelength gaps between bands) and increasing the number of bands, while also reducing their band widths, to yield spectra linked to known physical or chemical attributes, when including specific diagnostic wavelengths. Although imaging spectrometers (IS) have had a long history in airborne research, dating back to 1982 [46], they have only recently become available from satellite platforms. The terms imaging spectrometer and hyperspectral imager are often used interchangeably. The term hyperspectral literally means “many bands”, not necessarily contiguous, while “imaging spectrometer” is the technical term for contiguous (no gaps) “bands” for every pixel. “Full-spectrum” spectroscopy refers to analyses made using the VSWIR spectrum (~380–2500 nm), linking the spaceborne observations to the long history of basic laboratory and field spectroscopy research. Additionally important is the computer hardware and processing power required to ingest, process, integrate, and automatically interpret the enormous data volumes generated by these satellite instruments/platforms, especially when collected over time, are finally increasingly possible. These technology enhancements are needed to achieve critical Earth Science research priorities that enhance understanding of key environmental and societal processes [47,48,49,50,51,52,53,54].

#### 2.1.2. Active Instruments

Active instruments have internal energy sources and are not reliant on solar energy for data collection. They emit pulses of photons in particular frequencies (wavelengths) and measure the photons that are [53,54] backscattered to the detector(s), utilizing a wide range of frequencies (from 75 GHz to 0.215 GHz) and long wavelengths (from 0.25 cm to 1.3 m). A major advantage of active instruments is that they can be operated at night, and because the atmosphere is mostly transparent for them, they can be used in cloudy, icy, and stormy weather, and “atmospheric correction” is usually not needed. These instruments can also be flown at most altitudes used for orbiters. The main types of active systems utilized on satellites are lidar and radar imagers, and microwave sounders.

##### LiDAR (Light Detection and Ranging) Imagers

These are active sensors that utilize lasers with internal energy sources to emit pulses of photons in the VIS or NIR wavelengths that best fit specific science questions. For terrestrial scanners (e.g., for mapping forest structure), a NIR wavelength at approximately 1050 nm is used for both emitting and detecting a returned signal, whereas a VIS wavelength in the green region (475–575 nm, often around ~550 nm) is used over aquatic and ice environments, such as bathymetric lidars. Other wavelengths are used in ground-based lidars, and some could potentially be used in spaceborne lidars. Lidar technology has been expanding to other wavelengths for physiological information in addition to 3D information, e.g., [55], although none yet have been flown in space. Lidars give precise information about the height of an object and its distance from the sensor (based on the universal law relating time, energy, and distance: E = mC^2^). However, lidar measurements for distances and heights determined from space depend on accurate knowledge of a satellite’s position and pointing direction, so that by measuring the time interval to receive the reflected (backscattered) signal, the three-dimensional position of an object can accurately be determined. Lidar instruments are restricted to lower orbital altitudes at or below approximately 600 km, due to the signal strengths.

##### Radar and Microwave Imagers

The most widely used active systems are radar and microwave instruments, which have previously been flown on both weather and military satellites but are new to EO applications. Radar data are sensitive to the three-dimensional structure of terrestrial surfaces (ground micro- and macro-topography, vegetation, ice, and snow fields) for height and associated horizontal patterns created by distance and height differences between objects. Radar is secondarily sensitive to changes in the dielectric constant of materials, primarily of water in its liquid and solid forms and mixtures of these phases. Radars are often employed for weather observations but are also dedicated to environmental applications, such as measuring ice sheet thickness, forest structure, geomorphology, urban and built environments, and even agricultural applications. Historically, radar has been more typically flown for weather, atmospheric composition, climate, or oceanographic research and less commonly used in environmental research [56,57,58].

While most EO imagers acquire their information for specific wavelength intervals within the solar spectrum, thus in wavelengths of nanometers (10^−9^ m) or micrometers (10^−6^ m), radar and microwave data are generally described in frequency units, typically GigaHz (GHz) or MegaHz (MHz). But these units are also characterized in terms of an alphabetic naming convention that was established in World War II and still used (Table 1). We also include the corresponding wavelengths for the frequencies since this is the common unit of measurement for VSWIR and TIR measurements. 

K-band radar and microwave instruments are often used for measuring atmospheric precipitation, while X-band is most sensitive to materials or properties whose size match the sensor’s bandwidths (e.g., centimeters) like foliage, soil roughness, and ice surface roughness. With bands at longer wavelengths (e.g., C, S, L), can achieve greater penetration through porous media (e.g., a forest canopy). Thus, C and L bands are mostly used to measure terrestrial observations of vertical and horizontal topographic structure and forest height structures, with C-band returns more likely to describe “within canopy” structure, whereas the L band is more likely to measure backscatter from closer to the ground surface. More recently, there is growing interest in using the S band for topography, vegetation height, and horizontal gap structures, and especially for urban planning and development, and many other applications. The longest wavelength radars are P band, which are of particular interest to describe larger-scale topographic structures, such as penetrating the soil to obtain measurements of deeper soil moisture under forests and agricultural crops and also the full 3D measurement of forest canopy structure. While not yet flown on civilian satellites, a P-band radar instrument will be utilized by the upcoming Biomass mission, ESA’s 7th Earth Explorer, with an expected launch in 2024. 

### 2.2. Types of Satellite Orbits

A wide array of EO data are needed for the comprehensive research conducted by various scientific, application, and monitoring communities. These include investigations of weather and climate changes in the phases of water: ice, liquid and vapor, vegetation types and fractional cover, changes in vegetation/ecosystem condition, soil types and geochemistry, water quality and quantity, volcanic activity, geologic weathering, and a suite of atmospheric constituents and properties (e.g., ozone, carbon dioxide, methane, dust particles), and vegetation properties (biomass, chlorophyll content). To address these purposes, satellites are flown at different altitudes, inclination angles (relative to the equator), and eccentricities (deviation from a perfect circle).

**Figure 2:**
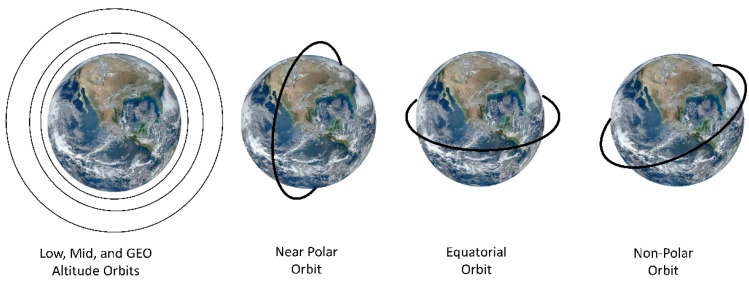
Orbit types for public sector EO satellites. The far-left panel shows designations for relative altitudes above the surface, for low, mid, and GEO satellites. The next panel shows the orientation for a pole-to-pole “near-polar” orbit. The third from the left is the Equatorial orbit that follows the equator around the planet, most frequently used for GEO altitudes. The right-most panel shows a non-polar and usually elliptical orbit, representing any angle away from near vertical. The example shows an orbit like that of the International Space Station, 516.6° N to 51.6° S, which currently hosts several experimental EO instruments.

Public sector satellites tend to fall into a few discrete categories, defined primarily by their orbital altitudes (Figure 2): low Earth orbit (LEO), between the top of the atmosphere at ~180 km up to 2000 km; medium or mid-Earth orbits (MEO) between 2000 and 35,780 km; and geostationary (GEO), high altitude Earth orbits, at ~36,000 km, such as used for weather satellites. [For reference, commercial airlines fly within the atmosphere below ~64 km]. LEO is used for most EO imaging instruments observing Earth surface properties for science and application studies and are most often “sun-synchronous”—meaning their overpasses occur at a fixed time of day. MEOs are near circular or elliptical in shape and used for Global Positioning System (GPS) satellites and other instruments. Satellites in MEO are semi-sun-synchronous if they complete each orbit in 12 h, thus viewing the same spot twice daily; MEOs can also be elliptical in shape. Satellites placed at GEO (or geosynchronous) altitudes are positioned at stationary points above the Equator to acquire relatively large-scale observations on a fixed schedule (e.g., hourly, or even continuous observations) of the same surface areas. Each GEO satellite rotates around the equator once every 24 h at a higher speed than the Earth turns (~11,100 km/h) to maintain its position. This orbit has until recently been used exclusively for weather observations, but the opportunity to make continuous diurnal observations is now of interest for future Earth Science applications.

Most EO satellites in LEO are flown in circular, polar (or near-polar, pole-to-pole) configurations. The actual altitude selected for a LEO polar mission (e.g., within 600–850 km) largely determines its repeat observation frequency interval (or temporal resolution) along the orbit’s central path line (but see swath width influences, below), usually separated by days or weeks. Repeated observations per location made from polar, sun-synchronous LEO satellites always occur at the same time of day. This is chosen based on the mission requirements (e.g., often ~10:30 am or ~1:30 pm local times), such as to avoid high % cloud cover or sun glint off water bodies, and to capture specific phenomena. Since the overpass time varies by as much as a couple of hours as a satellite proceeds along its pole-to-pole transit, and due to different time zones, the overpass time is represented by the local time it passes over the equator (i.e., the equatorial overpass time). LEO satellites flown in near-polar orbits travel at ~27,500 km/h and complete one orbit every 99 min, or 14 orbits per day while surveying semi-vertical curved slices across the whole Earth. During daylight hours with morning equatorial overpass times, each orbit’s descending half pass from the N pole to S pole (NE to SW direction) (e.g., the US/Landsat and ESA/Sentinels), with the corresponding ascending half pass occurring at night while traversing from the S to N poles. The time of day selected for overpasses, such as mid-morning or early afternoon, depends on the mission requirements.

Two other important factors affecting orbital paths are the inclination angle relative to the equator (e.g., 97.5°) and the eccentricity, or degree of deviation from a perfect circle. The orbits themselves can be circular (the most common for scientific applications) or elliptical, such that they are closer and farther from the Earth’s surface in different parts of the orbit. In addition, some satellites are flown in non-polar, elliptical orbits at large displacement angles from the poles (such as the International Space Station (ISS), which is inclined 51.6° away from the poles), for which both the revisit interval and time of day or night for the satellite overpasses vary over a particular location, but in a predictable way. Consequently, this type of orbit is non-sun-synchronous.

In addition to a LEO satellite’s altitude, the repeated observation interval per pixel depends on its position within the swath width (i.e., distance from the orbital path). The swath width is the edge-to-edge dimension of the area viewed (e.g., 30 m, 2 km, 50 km), and is perpendicular to the satellite’s flight path. When images are collected across narrower swaths (e.g., Landsat), a strategy that enables higher spatial resolution, each pixel is viewed with similar straight-down near-nadir view angles, perpendicular to the surface (view angle ≈ 0°; or 0° ± 20°). Since most satellites have square pixels, it is customary to only state one pixel length, such as 30 m, 500 m, and 1000 m. Typically, those satellites collecting data across these narrow swath widths take 14–28 days to return to the same spot at mid-latitudes (e.g., the USA’s Landsats and ESA’s Sentinels), except that more frequent converging overpasses do occur near the poles due to overlaps in adjacent flight paths.

In contrast, satellite instruments with wide swaths can obtain more frequently repeated collections (e.g., twice daily (day and night passes), daily to weekly). This is typical for satellites that view thousands of km on either side of their flight paths by sweeping in a side-to-side (or “whisk-broom”) motion across each swath (e.g., MODIS on Terra and Aqua); even wider synoptic views are used by atmospheric chemistry satellite instruments (e.g., the spectrometer carried by GOME, Global Ozone Monitoring Experiment, 1995–2011). However, there are several disadvantages of this type of mechanical side-to-side whisk-broom sweeping method: (i) it produces increasingly larger variations in view angles and pixel sizes that become largest at the extreme edges of the swath, for which corrective algorithms must be applied to better describe the per-pixel information; (ii) the dwell time over any pixel is short, limiting the number of photons that can be collected during the viewing time interval (thus limiting data quality); and (iii) the imager can incur a risk of mechanical problems from the sweeping motion. 

Newer “push-broom” satellites have the same number of detectors as pixels along the swath, so collecting spectral data perpendicular to the flight path is referred to as “mowing the lawn”. This method is utilized for relatively narrow swaths (e.g., <50 km) although the new Teledyne 2CHROMA-D detectors planned for the NASA SBG mission will enable a 185 km swath at a flight altitude of 705 km [17,59], equivalent to today’s Landsat satellites, thereby allowing simultaneous (near) nadir-viewing of all pixels along the swath. The “dwell-time” for push-broom detectors is longer because no time is lost due to side-to-side sweeping, thus they collect significantly more photons while the satellite is passing over the viewed ground area than is possible with the whisk-broom configuration. A bonus advantage is that greater radiometric sensitivity for the data is achieved. Lastly, since push-broom sensors do not have the mechanical back-and-forth swinging of the detector arrays, the potential for mechanical failures is reduced. For these reasons, all recent EO imagers on LEO satellites have adopted push-broom arrays [1].

Earth observations are also made from another distant position in space, from which the whole Earth is viewed as a disc and is always illuminated. This is possible at the Lagrange Point 1 or L1, the point in space located approximately 1.5 mil km from Earth in the direction of the Sun. Currently, NOAA operates the Deep Space Climate Observatory (DSCOVR, based on an earlier concept—Triana, unofficially known as GoreSat) launched in 2015 for Earth observations, space weather and space climate to warn Earth in the event of solar magnetic storms.

### 2.3. Statistics on Current Satellites in Orbit

The Union of Concerned Scientists (UCS, https://www.ucsusa.org (accessed on 12 January 2024)) maintains the record of operational satellites. As of 1 May 2023, they reported that there were 7560 such satellites. Of these, 6768 are in LEO, 590 in GEO, 143 in MEO, and 59 in elliptical orbits. Most these satellites, approximately two-thirds, are used for communications. Satellites for all other categories comprise about five to seven percent of total satellites, including Earth observation and remote sensing. Approximately 81 countries have launched satellites. The USA has the largest number of satellites in orbit with ~5184 (currently operating): 4741 commercial, 167 government, 30 civil, and 246 military satellites. Russia has 181 active satellites, China 628, and all others 1572.

In this paper, we discuss the current ~90 on-orbit EO satellite missions designed for scientific and/or environmental studies, primary those from the US and Europe with a few from Japan and several others that are making important Earth observations. We place special focus on the emerging new imaging spectrometers and other new technology in space. In addition, we cover the European Union’s Copernicus program and its 20+ new missions, six EO missions now hosted on the ISS, and some important commercial satellites supporting EO monitoring studies. We also highlight the new strategies for upcoming EO satellite missions in 2024/2025 to the early 3030s, and those expected over the next decade.

## 3. High-Impact Pioneering Satellites from the 1970s to the Early 2000s

We start with a look back to the pioneering EO satellites, with a focus on imagers. These include satellites from the USA (e.g., NOAA, USGS, NASA), and relevant European imaging missions, including the Copernicus Sentinels. Our review places special attention on passive EO spectral imagers because they are the most common types in space for providing biological/environmental mapping. We start with a look back to the pioneer satellites now retired from service, both small and large, that demonstrated the value of both passive imagers and active sensors from spaceborne platforms, including the AVHRR/NOAA satellites, the early Landsat series (USA), the relevant European missions such as EnviSat (ESA), the NASA Earth-Observing System (EOS) Flagships, and several others. The imagers carried on some of these satellites were often flown with other instruments, and they may not have been the primary mission focus, but nevertheless revealed potential for environmental payoffs that drove technology improvements. We consider many of the EO missions conducted over the past 50 years (~1970–2020) comprise a set of exploratory test beds for technologies, addressing a range of measurement types, alone or in various combinations, to find out what works and what is feasible, and which technologies deserved further development. Over time, the importance of imagers, especially when coupled with supporting measurements, has grown. Another reason for including some historic satellites is because these are the datasets available for time series studies, providing our best record of what has changed over the Earth in the past half century, related to patterns of land use and climate change.

### 3.1. The AVHRR on the NOAA POES and EUMETSAT METOP Satellites

NOAA’s satellite fleet is primarily focused on weather and climate observations and not on environmental applications. However, the first imager in space to provide global EO data useful for monitoring vegetation, and to cover phenomena like floods and volcanic activity (Table 2), was the Advanced Very High-Resolution Radiometer (AVHRR) which was flown on NOAA’s Polar-Orbiting Environmental Satellite (POES) series beginning with TIROS-N in 1972 [60]. Reprocessed nadir AVHRR data with 1.1 km pixels have been archived continuously since 1981 (https://www.USGS.gov/EROS/Archive/AVHRR, accessed on 13 December 2023), with weekly and biweekly data from 1989 to 2019 acquired by [60] for a series of 13 satellites with two to six wide (100–400 nm) VNIR bands, with a variable afternoon overpass time (13:30 pm ± 4 h). Two of these wide AVHRR bands (VIS and NIR) were used to calculate the Normalized Difference Vegetation Index (NDVI), which provided global seasonal and interannual phenological patterns of plant growth for the first time [61,62,63] and subsequently variations in this index have been applied to data acquired by many other spaceborne imagers. These images were brought into the environmental research portfolio by Dr. C.J. Tucker at NASA Goddard Space Flight Center. He began routinely processing these data to show phenological patterns of vegetation in the late 1970s, and the database was continued to 2019, when the NOAA series was discontinued and the responsibility for this class of observations was transferred to the European EUMETSAT’s METerology Operational Program (METOP, begun in 2006).

**Figure 3:**
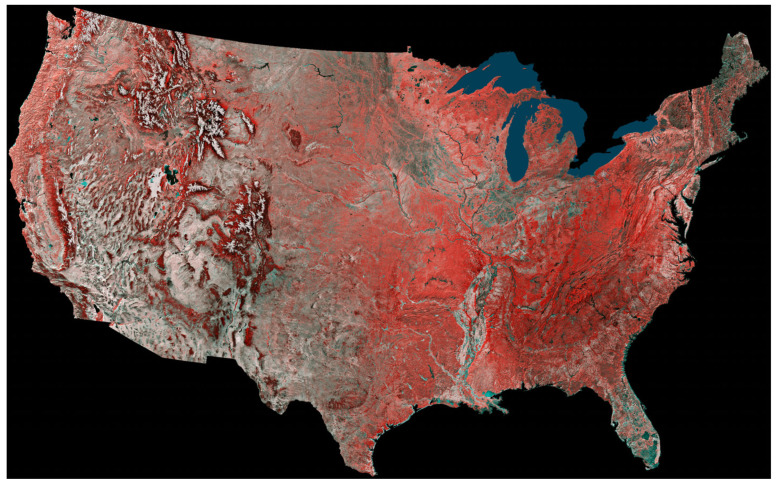
The Advanced Very High-Resolution Radiometer (AVHRR) was unique (from the 1970s to the 2000s) in being able to show continental to global-scale information (density and phenological patterns) about vegetation. This image is a mosaic of the continental U.S., composed of 16 AVHRR images (from NOAA-8 and -9) acquired between 24 May 1984 and 14 May 1996. It is displayed as a False Color Infrared with the NIR band displayed in the red color channel and two other bands (for AVHRR/2 that would be red, MidIR or TIR bands displayed in blue and green colors), Because vegetation has high reflectance in the near-infrared, images created with this color scheme show all vegetated areas as shades of red. Some of the reds are due to the type of vegetation (herbaceous, and broadleaf or conifer), but the more intense the color the higher the vegetation density. The image was created from multiple AVHRR images collected from 24 May 1984 to 14 May 1986. (CREDIT: U.S. Geological Survey (https://solarviews.com/cap/earth/usa.htm/ (accessed on 15 May 2024))).

Figure 3 shows a different vegetation index that goes back to the earliest days of infrared aerial photography. The “false color” combination of the VIS bands in the green and red combined with a near-infrared (NIR) band. The bands are “color shifted” to allow human eyesight to “see” the reflectance patterns in the NIR band. This is typically done by displaying the green band in blue (assuming it is printed or shown on a computer screen), red band in green, the near infrared band is displayed in the red color channel and for AVHRR/2 a second NIR band id is displayed in the blue color channel). The reason this band combination was adopted is because all vegetated pixels will be some shade of red since plants have low reflectance in the visible (about 5% reflectance) due to plant pigments and photosynthesis and have much higher reflectance in the NIR (about 50% or more). Figure 3 was composited at the continental scale for the period 24 May 1984 to 14 May 1986 for the AVHRR imager. In this image, grasslands and crops appear light red to red, conifer forests are dark red to maroon, deserts and urban areas are white to light gray, bedrock appears darker, and lakes and rivers are in shades of blue.

### 3.2. The METOP-A Satellite (2006–2021)

METOP-A was the first of Europe’s EUMETSAT Polar System (EPS) satellites (2006–2021) with an operational meteorology mission but also contributions to ocean and ice monitoring, climate monitoring and atmospheric chemistry (Table 2). METOP-A, -B (2012–2024), and -C (2018–2027) (https://www.eumetsat.int/our-satellites/metop-series (accessed on 17 September 2023)) all carry the AVHRR-3 provided by NOAA to EUMETSAT (well known for the Normalized Difference Vegetation Index, NDVI) and the Global Ozone Monitoring Experiment-2 (GOME-2), UV and NIR spectrometer (Table 3) to obtain concentrations of ozone, NOx, and other trace gases [64,65,66,67]. METOP-A and -B carried 10 other weather instruments and METOP-C carries 8 instruments in addition to AVHRR-3 and GOME-2. METOP-A flew in a descending orbit with an equatorial overpass at 07:50, while METOP’s-B and -C fly in a ~09:30 descending orbit. METOP-C, launched in 2018 carrying the last AVHRR instrument, which with NOAA, has maintained a continuous 40+ year global data record of land surface conditions, the longest satellite-based record of global monitoring [68].

### 3.3. The European Remote-Sensing Satellites: ERS-1 and ERS-2

ESA pioneered the tandem mission concept and imaging spectroscopy from space with several early missions (Table 3). Its first EO satellite program addressed ocean research with the European Remote Sensing (ERS) pair of satellites, ERS-1 (1991–2000) and ERS-2 (1995–2008). ERS-1 was the first of modern European EO satellites flown by the ESA for environmental monitoring, and complex for its time. It carried radar and microwave instruments for imaging and measuring the sea state and ice conditions to increase understanding of the oceans and coastal zones. Using these active “all weather” instruments allowed the satellite to observe areas of the Earth where there was frequent cloud and fog cover. ERS-1 operated for nine years and carried five instruments (Table 3). ERS-2 was launched in 1995 in the same orbit to establish a ~5-year tandem mission, passing one day apart, each with a 35-day repeat cycle. Both satellites carried identical instruments, although those carried on ERS-2 were upgraded versions, including a 13.8 Ku-band radar altimeter, a 4-channel SAR imaging radiometer, a microwave sounder (for sea-surface and cloud top temperatures), a SAR C-band polarization radar for detecting surface height (≤mm precision), an active polarization radar to measure wind speed and direction for 50 km cells, and a non-scanning passive Microwave Radiometer for atmospheric water vapor. ATSAR-2 discontinued the microwave bands but added three VNIR bands optimized for sea-surface temperatures and climate. The ERS-2 also had two new instruments: a simple imager—a 3-band VIS radiometer to detect ocean chlorophyll and vegetation that failed in 2008, and the important trailblazing instrument GOME-2 (Global Ozone Monitoring Experiment), a nadir scanning UV and VIS instrument which was the first in space to monitor atmospheric chemistry, including O_3_, SO_2_, NO_2_, HCO_2_, BrO, and H_2_O (Table 3). GOME pioneered a UV/VIS grating spectrometer for atmospheric chemistry with four UVNIR bands (with 4096 channels) and three additional broad bands over the same wavelength region. GOME-2 was intended to continue until 2011 but its on-board failures of gyroscopes in 2001 and a tape drive in 2003 limited its capability. 

### 3.4. The Project for On-Board Autonomy-1 (PROBA-1)

Another impressive milestone was the launch in 2001 of the Project for On-Board Autonomy-1 (PROBA-1). It was a small (<1 m^3^), agile technology demonstration mission carrying the first high-resolution imaging spectrometer in space. Intended as a one-year mission, it continued for two decades. Although PROBA-1 is still operating today, its primary instrument—CHRIS (Compact High-Resolution Imaging Spectrometer)—was suspended at the end of 2022, but PROBA-1. CHRIS-PROBA (Table 3) flew in a sun-synchronous polar LEO with a narrow swath width but with a 7-day repeat period by pointing the satellite fore and aft. This important early EO imaging spectrometer collected BRDF (bidirectional reflectance distribution function) data across the VNIR spectral range (400–1050 nm) in 150 channels that could provide simultaneous measurements of 62 spectral bands at a GSD of 34 m. Spectral information could be provided in custom bands, as specified by a user. The nominal product, however, was 18 bands at a GSD of 18 m for square images of 13 km^2^. Each target area was viewed at five angles (±55° ± 36°, 0°) with five consecutive push-broom scans made with its detector in a single line array (earth.esa.int/eogateway/instruments/chris (accessed on 23 December 2023)), and [69].

### 3.5. Envisat: ESA’s Pioneering Atmospheric and Land Platform

Envisat was ESA’s large, pioneering 11 instrument atmospheric and land platform, placed in polar-orbiting LEO space in 2002 (Table 4). Envisat was a multi-instrument general purpose platform that was complementary to the NASA Terra platform (Section 4.2.1). It flew during the decade from 2002 to 2012 (earth.esa.int/eogateway/instruments/Envisat (accessed on 23 December 2023)) in a sun-synchronous descending orbit (10:00 equatorial crossing time) at 790 km altitude. Envisat included two radar instruments, a C-band all weather multi-polarization Advanced Synthetic Aperture Radar (ASAR) for ocean land, and ice. And a two frequency (3.2 GHz (S-band), and 13.6 GHz (Ku band) Radar Altimeter-3 for ocean topography, wave height and wind speed. The Advanced Along-Track Scanning Radiometer (AATSR), derived from ATSR on ERS-1 for accurate seas surface temperature and climate data. Three imagers of different types and measurement characteristics (the MERIS a 15 channel 300 m resolution, MERIS (Medium-Resolution Imaging Spectrometer) for monitoring ocean, land, aerosol and cloud properties, and the SCIAMACHY (SCanning Imaging Absorption spectroMeter for Atmospheric CartograpHY), both nadir and limb scanning for measuring the concentrations of atmospheric chemical species. Lastly, it included two radiometers (broad and narrow band), two ranging instruments, and a high-resolution interferometer (Michelson Interferometer for Passive Atmospheric Sounding, MIPAS). Envisat provided complete global coverage in 1–3 days with 8 (out of 10) of its wide-swath instruments. This included its two VSWIR spectrometers, which were of greatest interest for environmental research and applications: MERIS (Medium-Resolution Imaging Spectrometer) and the ground-breaking SCIAMACHY (SCanning Imaging Absorption spectroMeter for Atmospheric CartograpHY), both a limb sounder and a nadir sounder. SCIAMACHY measured 16 tracked chemical species and aerosols [70,71], with heritage from the GOME-2 instrument on ERS-2 (Table 4).

#### 3.5.1. Medium-Resolution Imaging Spectrometer (MERIS)

**Figure 4:**
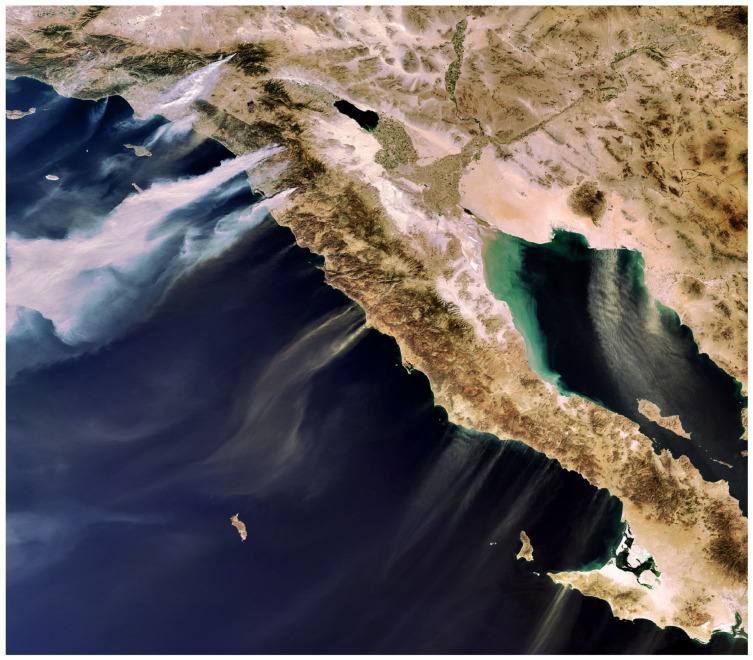
MERIS was a near daily high-resolution wide-swath imaging instrument on the Envisat satellite. This figure captured southern California and northern Mexico, including the Baha peninsula on 22 October 2007 at 17:52 UTC showing smoke plumes from multiple wildfires in southern California and dust plumes in Mexico blowing westward over the Pacific Ocean caused by Santa Ana winds blowing from regions of high pressure in the hot interior desert to low pressure along the coast. The elongated lake east of the smoke plumes is the Salton Sea, California’s largest lake. At the top of the Gulf of California, there is a large algal bloom along the Baha Peninsula and two smaller blooms are seen below the wind-blown dust from the Mexican mainland. [Credit: ESA, File: https://www.esa.int/Applications/Observing_the_Earth/Envisat/Envisat_captures_California_ablaze/ (accessed on 16 May 2024)].

The primary mission for MERIS was to study ocean color, which it did until 2016. A unique feature of MERIS was the programmability of its narrow spectral bandwidths (2.5–30 nm) and wavelength positions within its VNIR range (390–1040 nm). Its swath width was 1150 km, with a relatively large mid-swath nadir spatial resolution (1400 m × 1200 m for oceans, and 260 m × 300 m for land observations) (Table 4). MERIS had five cameras, each equipped with a push-broom spectrometer to acquire 2D images. Although bands were programmable per spectrometer for wavelength position, width and gain, useful data were only obtained/provided in 15 spectral bands. MERIS obtained oceanographic measurements of high-resolution ocean color to derive chlorophyll and suspended sediment concentrations (and later, atmosphere and land surface studies); phytoplankton blooms were easily detected by MERIS (Figure 4). MERIS provided the first space-based evidence for chlorophyll fluorescence from land-based vegetation [72].

#### 3.5.2. Scanning Imaging Absorption Spectrometer for Atmospheric CartograpHY (SCIAMACHY)

**Figure 5:**
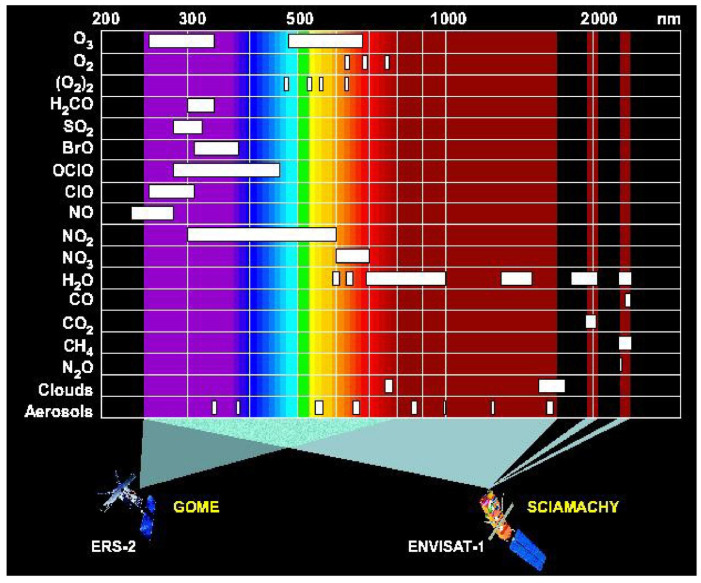
Narrow spectral bands measured over a wide range of wavelengths (from UV to SWIR, measured by the SCIAMACHY instrument (2002–2012 on Envisat-1). The narrow bands made it possible to detect many atmospheric gas molecules, and aerosols, plus cloud properties. This figure shows the wavelength intervals (white boxes) important to uniquely identify these gases. The wavelength scale is shown along the top of the figure. White vertical lines mark 100 nm intervals, between 200 and 1000 nm, after which the scale jumps to 2000 nm and ends at 2500 m). The colors represent regions of the spectrum: purple (UV); blue, green, yellow, orange, and red (visible spectrum); dark red indicates the SWIR region. The comparable shorter-wavelength range is shown for ESA’s GOME (Global Ozone Mapping Experiment) instrument from a decade earlier on ERS-2. Figure from https://www.iup.uni-bremen.de/sciamachy/ (accessed on 12 November 2023).

SCIAMACHY had heritage from the GOME-2 instrument on ERS-2. It is important because it was the first instrument in space to measure 16 atmospheric chemicals, plus detecting aerosols, cloud properties and surface materials (Table 4). SCIAMACHY measured eight wavelengths regions referred to as “bands”, which each were sampled at high spectral resolution (Figure 5) with 2 UV band regions (240–314 nm and 309–405 nm, with ~0.25 nm spectral resolution); a VIS band region (394–620 nm, with 0.44 nm resolution); a VNIR band (604–805 nm, with 0.48 nm resolution); a NIR band (785–1050 nm, with 0.54 resolution), and three bands covering the MIR and SWIR regions: 1000–1750 nm (with 1.49 nm resolution); 1940–2040 nm (with 0.22 nm resolution); and 2365–2390 nm (with 0.26 nm resolution). SCIAMACHY measured aerosols and trace gases in both the troposphere and the stratosphere, including (BrO, CH_4_, CIO, CO, CO_2_, H_2_O, HCHO, N_2_O, NO, NO_2_, NO_3_, O_2_, O_3_, O_4_, OCIO, SO_2_) and aerosols (https://earth.esa.int/eogateway/instruments/sciamachy/description/ (accessed on 13 November 2023)).

SCIAMACHY measurements were made at nadir through the atmosphere, observing relatively small pixels, 30 m wide across the swath and 60 m long. Flight segments were 25 km long. The limb-scanning component measured a ±500 km horizontal sector with 3 km vertical resolution that also measured solar and lunar occultation for self-calibrating. Measurements in daylight hours were separately obtained for the stratosphere and tropospheric zones, to derive the total atmospheric column. At night, SCIAMACHY observed biomass burning, volcanic eruptions, and air glow. For calibrations, measurements were made for limb and solar/lunar occultation geometries. The many applications of SCIAMACHY data include tracking NO_2_ from fossil fuel combustion and CO_2_ emissions [71,73] and methane emissions [74]. SCIAMACHY was a collaboration between the Netherlands Space Office (NSO) and the Belgian Federal Science Policy Office (BELSPO) to improve understanding of the Earth’s radiation budget and was developed at the German Aerospace Center (DLR).

### 3.6. The JAXA Greenhouse Gas Observatory Satellites (GOSAT’s 1 and 2)

GOSAT is also known by its Japanese name, “Ibuki” (breath). It was designed to estimate absorptions of greenhouse gases on a subcontinental scale (thousands of km^2^). Column estimates of CO_2_ and CH_4_ abundances (number of gas molecules/column/surface area) are calculated from the observational data. GOSAT was launched in 2009 and is still operating in early 2024 (Table 4) at ~674 km altitude after nearly fifteen years, carrying two instruments. The primary instrument is the **I**nfrared **F**ourier **T**ransform **S**pectrometer (**TANSO-FTS**) to study greenhouse gases with target species during daylight (clear sky conditions) hours for band 1 (O_2_), band 2 (CO_2_, CH_4_), band 3 (CO_2_, H_2_O) and band 4, which is both a day and night band with 7 channels to measure CO_2_ and CH_4_. The second instrument is a cloud and aerosol sensor, the **T**hermal and **N**ear-**I**nfrared **S**ensor (**TANSO-CAI**). Ibuki obtains spectral observations over the NIR and TIR regions during the day, with clouds and aerosols target species. These data are used to correct effects of aerosols and clouds on the TANSO-FTS measurements. 

GOSAT-2 was launched in 2018 for a five-year mission (Table 4), and carries the upgraded Thermal And Near infrared Sensor for carbon Observation (TANSO-FTS-2), a Fourier Transform push-broom Spectrometer that makes measurements of atmospheric pressure over land and sea surface, measures the ozone mole fraction, the methane mole fraction, the carbon monoxide mole fraction, deuterium water vapor (HDO), and chlorophyll fluorescence from vegetation on land using the Global Climate Observing System (GCOS) definitions. The TANSO-CAI-2 measures aerosol optical depth and cloud cover following GCOS, and the Normalized Difference Vegetation Index (NDVI).

### 3.7. NASA/USGS Landsat-7, the Last of the Thematic Mapper Series

Landsat-7 (1999–2024) carries a whisk-broom scanning imager—the Enhanced Thematic Mapper Plus (ETM+), and is an improved version over the earlier Landsat Thematic Mapper instruments onboard L-4 and L-5 (Landsat-7|U.S. Geological Survey (usgs.gov)) (Table 5). The ETM+ has eight relatively wide spectral bands: six of these collected data from 30 m pixels: four VNIR (blue, green, red, NIR), and SWIR and MIR bands. There is also a thermal band (60 m) with low and high gain options, and a VIS panchromatic band (15 m). Image products are delivered as 8-bit images with 256 grey levels. Onboard calibration capability was added with a Full-Aperture Solar Calibrator (FASC) and a Partial Aperture Solar Calibrator (PASC), in addition to the 2 calibration lamps.

## 4. Pioneering NASA Missions, EOS-1 and the EOS Flagship Satellites from Early 2000s

### 4.1. NASA’s Demonstration Satellite, Earth Observing 1 (EO-1)

This pioneering satellite was launched in November 2000 for a planned one-year technology demonstration under NASA’s New Millennium Program (NMP). EO-1 carried two innovative imaging instruments, the **A**dvanced **L**and **I**mager (**ALI**) and **Hyperion**, both of which collected imagery at the Landsat 30 m spatial scale (Table 5). Because it collected remarkable new image data from both of its instruments, and because ALI provided a back-up to the ailing Landsat-7 in the decade before the launch of Landsat-8, the EO-1 continued for 17 years as an extended mission (2000–2017).

#### 4.1.1. Hyperion on EO-1

The **Hyperion** instrument, the first civilian imaging spectrometer in space, was a full-VSWIR-spectrum (357–2576 nm) instrument with data collected by two side-by-side grating spectrometers to provide 220 contiguous 10 nm spectral bands (Table 5). Because it was a technical demonstration project, the Hyperion had a narrow 7.7 km swath and an along track image length of 42 km (optional 185 km length to match the Landsat scene length).

This technology-demonstration satellite originally flew in a 10:30 morning orbit in a tandem formation one minute behind L-7 (and also with the Flagship Terra). EO-1 images were linked to the Worldwide Reference System. However, due to its limited duty cycle, EO-1 only collected specific images as requested by users for science and application studies. Since these study sites were distributed globally, they have provided a unique archive of VSWIR measurements of various surface types sampled around the globe, based on both multispectral data (ALI) and Hyperion spectra [75,76,77,78,79] (EO-1 served as a key pathfinder that led to technological advances in instruments flown in space today (e.g., Landsat-8 and 9, EMAP, Prisma, and EMIT, all discussed in later sections) and those expected to fly over the next several years.

#### 4.1.2. Advanced Line Imager on EO-1

The **ALI** successfully demonstrated several technology upgrades that were Landsat-7’s ETM+ suffered a Scan Line Corrector failure in May 2003 that affected approximately 25% of each image; however, data processing algorithms enabled science quality data for the unaffected image sectors, and it operated successfully afterwards (Table 5). Until early 2017, its original 705 km orbit and mid-morning equatorial crossing time were maintained. With its onboard fuel running low, a final inclination maneuver was performed in 2017, and L-7 subsequently began drifting in orbit, and was lowered by 8 km to 697 km in 2022. As of March 2024, L-7 has continued to provide limited nominal operations, having added >100,000 images to the USGS Landsat archive despite its equatorial crossing time having shifted earlier (before 8:00 AM MLT) and its images are no longer being directly linked to the Worldwide Reference System. Operations are scheduled to end in 2025 later incorporated into Landsat’s 8 and 9, although its swath width was limited to 37 km [79,80,81,82]. One of those most important technologies was the push-broom design of its multispectral imager which had relatively narrow 10 spectral bands, as well as a VIS panchromatic (PAN) band with 10 m spatial resolution. New ALI advances also included a four-mirror telescope with 12-bit quantization and a 30 km swath.

### 4.2. EOS Flagships: Missions Expected to End in 2025–2027

Drawing on lessons learned from the USA’s earlier Thematic Mapper Landsat’s (Table 5) and AVHRR discoveries (Table 2), the European satellite mission successes, e.g., MERIS on Envisat, and those of other nascent international space agencies, NASA undertook an unprecedented Earth-Observing System (EOS) program in the 1990s decade. This led to the placement of three large satellite Flagship platforms into LEO near-polar sun-synchronous orbits, Terra (1999) for land, Aqua (2002) for oceans, and Aura (2004) for atmosphere. All three platforms are still operating, although some instruments are not, and all are in drifting orbits, no longer maintaining fixed overpass times or altitude. No more inclination maneuvers or drag make-up maneuvers are planned, conserving remaining fuel for collision avoidance events. Passivation/decommissioning for each satellite is currently expected between 2025 and 2027.

#### 4.2.1. Terra

Terra was launched into a descending morning orbit originally at 705 km, with the equatorial crossing time at ~10:30 am local time in formation with Landsat-7 and NASA’s technology demonstration satellite, the Earth Observer (EO-1), all launched in late 1999 and operational in 2000. Terra carried five groundbreaking instruments for earth observation. Two were flown on both Terra and Aqua, MODIS a near daily global imager, with the thermal imager having both day and night coverage, and CERES, measuring the Earth’s radiation budget, continuing the earlier record from Earth Radiation Budget Satellite (ERBS). Terra also carried pioneering instruments: ASTER, a 14 band high spatial resolution imager with five thermal bands, MISR a multiangle spectroradiometer, and MOPITT (Measurement of Pollution in the Troposphere) a gas correlation spectrometerwith 15 channels. Near the end of its operational life, Terra has lost altitude (now ~690 km and shifted to an increasingly earlier crossing-time, now before 09:55), with decommissioning expected between late 2025 and 2027. For more than two decades, instruments carried on these platforms have accrued an incredible archive of environmental information about the Earth’s land, oceans, and atmosphere. 

#### 4.2.2. Aqua Flagship

**Aqua** (2002) originally flew in an ascending sun-synchronous orbit with a 13:30 pm equatorial crossing time, and was part of the “A-Train” formation along with the 3rd NASA Flagship, Aura. Aqua carried six instruments to study the Earth’s oceans, large water bodies, and the atmosphere, but MODIS was its only imager. In addition to MODIS and CERES, it carried AIRS, for temperature and humidity sounding, AMSR-E a multipurpose microwave imager with six frequencies and 12 channels that measured cloud properties, sea-surface temperature, near-surface wind speed, radiative energy flux, surface water, ice and snow, furnished by the National Space Development Agency of Japan. AMSU-A, a microwave sounder for temperature, with 15 channels including the 54 GHz band for all weather measurements. AMSU-A replaces the MSU on the NOAA TIROS-N and NOAA 9–14 (Table 2). The final instrument on Aqua was the Humidity Sounder for Brazil (HSB), a four channel sounder for humidity sensing.

Aqua experienced instrument anomalies with its Solid-State Recorder, which after 2007 could only hold two orbits worth of data. A series of solar array and related electronic anomalies that began in 2010 led to the loss of 23 of its 132 solar cell strings. A 2005 battery short circuit led to a partial loss of capacity, and in 2009 a solar panel thermistor failed. Several other anomalies (e.g., Dual Thruster Module-2 Heater) occurred but corrections and recoveries were mostly successful. Near the end of the mission, only three of its six instruments are still operating, MODIS, AIRS, and CERES, although each had or were experiencing moderate to serious issues. The three failed Aqua instruments were the Advanced Microwave Sounding Unit (AMSU-A) failed in 2016; the HSB from Brazil’s space agency, Instituto Nacional de Pesquisas Espaciais), a very high frequency-band instrument to measure atmospheric humidity), non-operational since early 2003 when its scan mirror motor failed; and the AMSR-E that failed in 2016.

#### 4.2.3. Aura Flagship

Aura (Latin, for breeze), the third NASA EOS Flagship (2004), is a follow-on mission to the Upper Atmosphere Research Satellite (UARS), considered to be the first in NASA’s Mission to Planet Earth series (1991–1999). Aura, a collaboration with the Netherlands, Finland, and the UK, is an atmospheric chemistry and air quality mission to study the Earth’s ozone layer, air quality and climate. Aura carries four instruments, but no imagers. There were two active sounders, the Microwave Limb Sounder (MLS) and the HIRDLS (HIgh-Resolution Dynamics Limb Sounder) to measure O_3_, H_2_O vapor, CFCs, CH_4_, and NOx using backscattered IR. The other two instruments are TES (Tropospheric Emission Spectrometer) to detect O_3_, CO, CH_4_, and NOx with IR wavelengths, and a UV/VIS spectrometer to detect ozone (OMI, Ozone Monitoring Instrument). Two of these instruments were decommissioned, TES in 2018 and HIRDLS in 2008. Only the MLS and OMI are still operating in Aura’s last years, but both have had technical issues. OMI produced high-resolution ozone distribution maps based on UV and VIS data, but it developed a “row anomaly” in 2007, limiting collection of radiance data. The MLS, which sampled over an altitude range of 5–120 km (in 1.5 km intervals), measures emissions from ozone, chlorine and other trace gases, with separate spectrometer modules for THz and GHz, each observing emissions across 20 bands. The THz module measures the OH radical in the stratosphere and mesosphere using heterodyne detection of thermal emission, but two of its bands became inactive, one for HCl and one for N_2_O. Aura suffered other setbacks and anomalies, including the loss of 33 of its 132 solar array strings and a solid-state recorder anomaly, ending housekeeping collections in 2017.

### 4.3. Some Important Instruments Carried on the NASA Flagships

#### 4.3.1. MODIS (MODerate-Resolution Imaging Spectroradiometer)

Perhaps the most important EO imager for science investigations thus far is the MODIS imager on both the Terra and Aqua Flagships (Table 5). Each MODIS is a full-spectrum spectroradiometer for VIS through TIR wavelengths (0.405–14 μm) but with relatively wide spectral bands (≥20 nm) and spacing between bands. The MODIS imaging spectroradiometer has 36 spectral bands, twenty reflective VIS/SWIR bands and sixteen TIR bands. Bands were selected to provide important information about the land, ocean, ice, and atmosphere, and are frequently referred to as “land bands” or an “ocean bands”, etc., in contrast viewing them as a continuous spectrum. The global sampling capabilities pioneered by these MODIS imagers (≥500 m^2^–2 km^2^), much coarser spatial resolution than Landsat but finer than the polar weather satellites, underlie their success providing global environmental monitoring during the first two decades of the 21st century (1999–2024+). 

Both platforms have the “NDVI bands” with 250 m GSD (band 1, 645 ± 25 nm; band 2, at 892 ± 18 nm) and five additional 20–50 nm wide “land” bands (two VIS, bands 3 and 4; three NIR/SWIR, bands 5–7) at 500 m GSD. There are ten narrow (10 nm) VIS bands plus one NIR band, primarily intended for capturing ocean color (bands 8 to 16). All 20 reflective bands were intended to support mapping investigations of vegetation, geology, soils, and/or water color and condition; three additional NIR bands (17–19) were added for quantifying atmospheric water vapor (band centers at 935, 940, and 905 nm) [83,84] that complemented the aerosol optical thickness retrieval over land using VIS wavelengths centered at 470, 560, and 650 nm [85]. The 16 TIR bands (3.660 to 14.485 µm) support atmospheric and cloud property studies at 1 km GSD [86,87,88]. Additional information is available (https://terra.nasa.gov/about/mission (assessed on 13 November 2023); https://aqua.nasa.gov/content/Instruments/ (accessed on 13 November 2023); https://aura.gsfc.nasa.gov/scinst.html/ (accessed on 13 November 2023)) [1]. MODIS level 1 data, geolocation, cloud mask, and atmosphere products are available at: http://ladsweb.modaps.eosdis.nasa.gov/cloud/ (accessed on 13 December 2023); other MODIS websites include land products (https://lpdaac.usgs.gov/ (accessed on 12 December 2023)), cryosphere products (http://nsidc.org/daac/modis/index (accessed on 12 December 2023)), and ocean color and sea-surface temperature products (http://oceancolor.gsfc.nasa.gov/ accessed on 12 December 2023)).

#### 4.3.2. CERES (Clouds and the Earth’s Radiant Energy System)

CERES is also hosted on both the TERRA and AQUA platforms (https://ceres.larc.nasa.gov/instruments/satellite-missions/ (accessed on 12 December 2023)) to measure the Earth’s broadband radiative energy flux (Table 5). The first CERES instrument was on the Tropical Rainfall Measuring Mission (TRMM) satellite that failed 9 months after launch in 1997 but improved upon the Earth radiation budget experiments of the 1980s (ERBS 1984, NOAA-9 1984) and NOAA-10 (1986). With both the Terra and Aqua platforms, CERES measured daily IR and total radiance four times daily and shortwave VNIR twice daily. Given the importance of the long-term radiative budget CERES satellites have been subsequently carried in different configurations on multiple satellites, including the NASA Suomi NPP and NOAA 20 satellites. The polar weather satellite JPSS-4 (to be called NOAA 24 after launch) in the 2027-time frame, will carry an advanced energy budget instrument, NASA’s Earth Venture mission, Libera. It will host a new split shortwave channel measuring radiation between 0.70 and 5.0 μm for the Earth’s radiation budget. CERES data are available from Atmospheric Science Data Center (https://asdc.larc.nasa.gov/project/CERES/ (accessed on 13 December 2023)). AIRS data are available at https://airs.jpl.nasa.gov/ (accessed on 13 December 2023).

#### 4.3.3. Spaceborne Thermal Emission and Reflection Radiometer (ASTER)

ASTER (Table 6), flown on Terra, was specifically designed for mineral identification, and secondarily for mapping other environmental conditions. It was designed to capture high spatial (15–90 m GSD) resolution geologic images for selected land regions with two VIS bands (15 m GSD), two 15 m NIR bands (nadir and backward views), six SWIR bands (30 m GSD), and five TIR bands (90 m GSD). However, the SWIR bands degraded and were terminated in 2009 but the VNIR and TIR bands still operated until September 2022. 

**Figure 6:**
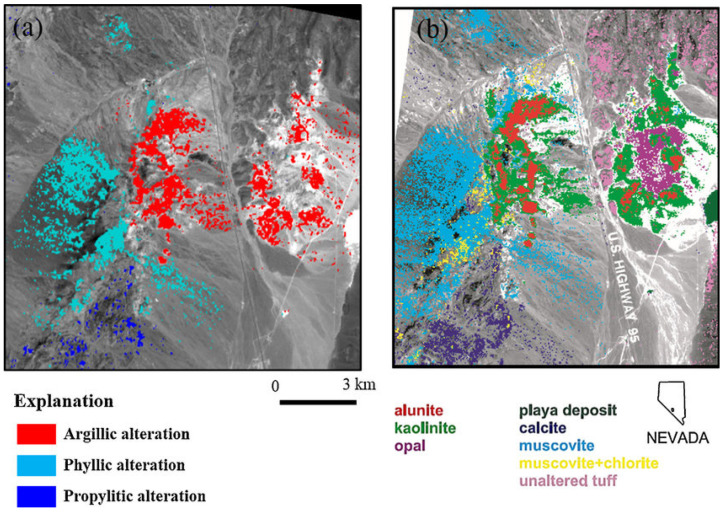
**ASTER** was designed for mapping geologic minerals, although it has many more applications. Its narrow spectral bands and high spatial resolution were a technical advance. This figure shows it is possible to identify many geologic minerals based on their spectral absorptions. This figure is from a complex, mineral-rich site in Cuprite, NV, USA. In this figure, three classes of alteration minerals are apparent in the hydrothermal zone, with alunite and kaolinite dominant in the opalized zone (**a**,**b**). Figure 6 reproduced with permission from Rowan et al. [91].

ASTER was not designed to provide global data/but was innovative because of its high-spatial-resolution spectral data, especially the 15 m VNIR bands, but also the six relatively narrow SWIR bands (40–100 nm wide, a technical advance in the late 1990s), which with the five thermal bands significantly contributed to identifying geologic minerals based on their spectral absorptance wavelengths [89,90]. Cuprite, Nevada is well known for its diverse mineralogy (Figure 6). This figure shows identification of different minerals and clays using ASTER data to identify them based on spectral bands where high absorption of energy can be measured.

ASTER data have been used to map virtually all land areas except the poles. Furthermore, the fore and aft-looking NIR bands were important for creating global digital elevation maps [92]. The ASTER Global Digital Elevation Model V3 (2009) covers from ±83° N and S latitudes, encompassing 99% of the Land surface (asterweb.jpl.nasa.gov/gdem.asp/ (accessed on 4 January 2024)) and the ASTER water body dataset (ASTWBD) is available from https://lpdaac.usgs.gov/products/astwbdv001/ (accessed on 4 January 2024) and from Japan Space Systems (https://www.jspacesystems.or.jp/en/project/observation/aster/ (accessed on 4 January 2024)). Both MODIS and ASTER data are available at the NASA Land Processes DAAC (earthdata.nasa.gov/sensors/modis, or earthdata.nasa.gov/sensors/aster/ (accessed on 4 January 2024)).

#### 4.3.4. Multiangle Imaging Spectroradiometer (MISR)

Terra hosts one of the first multiangle imagers, MISR (Table 6), which monitors trends in atmospheric aerosol concentrations [93], dust [94], and land surface properties [95] with nine separate digital cameras, each acquiring passive reflected radiation in four wavelength bands (blue, green, red and NIR). MISR data can distinguish different types of clouds and cloud motion vectors that are available through LANCE (Land, Atmosphere Near Real-Time Capability for EO), within 90 to 120 min after observation. One camera points directly downward (nadir) while a pair of forward and backward cameras view the Earth System in four directions (26.1°, 45.6°, 60.0°, and 70.5°).

#### 4.3.5. AIRS (Atmospheric Infrared Sounder)

AIRS is located on the afternoon Aqua platform, measured atmospheric temperature and humidity (Table 6), as well as land and sea-surface temperatures. AIRS was a grating spectrometer with 2378 channels in four measurement wavelength groups and five additional bands in the VNIR wavelengths for temperature and humidity sounding, providing daily global coverage. It also provided a vertical ozone profile and total-column greenhouse gases. Aqua’s highly calibrated suite of data products for radiance, reflectance, and actively induced backscatter provided climate-quality measurements collected for more than ~20 years, that in addition to scientific achievements, have been utilized to cross-calibrate other spaceborne sensors.

#### 4.3.6. TES (Tropospheric Emission Spectrometer)

TES both a limb sounder and a nadir spectrometer on the Aura platform, was designed to detect 21 chemical species CFC-11, CFC-12, HCFC-22, CH_4_, CO, CO_2_, C_2_H_2_, C_2_H_6_, H_2_O, HCl, HDO, HNO_3_, N_2_, NO, N_2_O, NH_3_, O_3_, OCS, SO_2_ PAN, SF_6_, and aerosols (Table 6). The TES-nadir spectrometer is composed of an imaging interferometer with four bands, comprising 43,750 channels. In the cross-track mode, it measures 16 detectors (of 0.53 × 0.53 km^2^ GSD) moving across 10 steps to cover a FOV of 5.3 × 8.5 km^2^ area. The array can be pointed within a cone of 45° or cover a swath of 885 km. The cross-track mode is an alternative to the limb mode. The TES-limb is an imaging interferometer with four bands and 162, 162 channels. It has 16 detectors that simultaneously observe altitudes of 37 km over 10 transverse steps from near the ground surface at 2.3 km IFOV and 23 km horizontal. The vertical resolution is 2.3 km and effective horizontal resolution is 300 km.

### 4.4. The International A-Train

**Figure 7:**
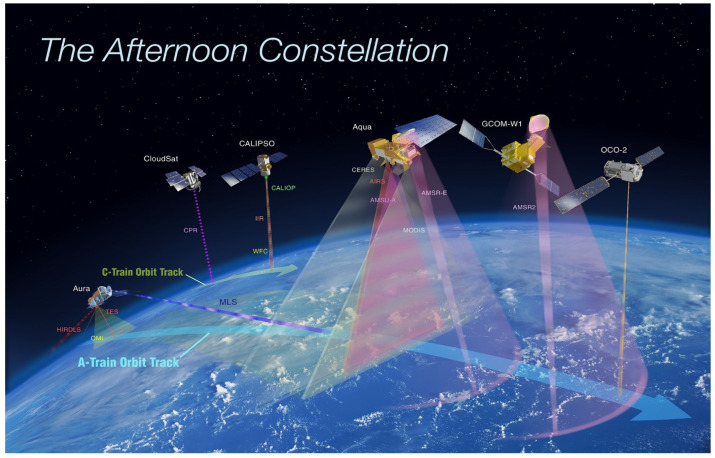
The international “A Train” satellite constellation. This schematic shows the six satellites in the formation from 2014 to 2018. The constellation of these satellites provided a comprehensive set of information about spatial and temporal atmospheric conditions for weather and other applications. The entire constellation passed within approximately 15 min of each other, and this demonstrated the value of including measurements that could leverage information across multiple instruments. The coordination of multiple datasets allowed new atmospheric and ocean parameters to be produced (NASA A-Train (http://hatrain.nasa.gov/ (accessed on 12 May 2024))).

This was a special formation comprised of seven satellites from several countries that flew in an afternoon constellation called the “A-Train”. Six of these seven satellites flew together in the A-Train between 2014 and 2018: Aqua (NASA), Aura (NASA), OCO-2 (Section 5.9), GCOM-W1 (JAXA), CloudSat (NASA), and CALIPSO (**C**loud-**A**erosol **L**idar and Infrared Pathfinder Satellite Observations, NASA and CNES) (Figure 7). The PARASOL (Polarization and Anisotropy of Reflectances for **A**tmospheric **S**ciences coupled with **O**bservations from a **L**idar, CNES) joined Aqua and Aura in 2004, but was lowered 9.5 km in 2011, and exited the formation in 2013. During their participation in this constellation, these satellites flew in close formation along the same ascending orbital track at 13:30 local solar time to provide coordinated and synergistic data for weather and climate. This afternoon satellite constellation was established in 2004 when Aqua (2002) was joined by both Aura and PARASOL (for seven years). CloudSat and Calipso had joined the A-train earlier (in 2006), but both were moved 16.5 km below the A-Train in 2018 into a lower formation, the C-Train. The A-Train was later joined in 2012 by GCOM-W1 (Japan) and in 2014 by OCO-2 (NASA), which became the lead satellite. The sequential order for these satellites was this: OCO-2 was followed by GCOM-W1 (293.5 s later) and Aqua followed next 150 s later, then CALIPSO was approximately 40 s after Aqua, next CloudSat followed 103 s later, [in early years, PARASOL was 1 min later], and Aura was the last satellite at 337 s later. The entire “train” of satellites required 14 min 56.5 s to pass over. Each individual mission had its own science objectives, but together they synergistically improved our understanding of the Earth’s climate, e.g., [96,97,98,99,100]. As of 2024, only OCO-2 and GCOM-W1 remain in orbit.

## 5. Important Research Satellites Launched since 2010

Next, we highlight eight satellites launched since 2010 for research purposes that explored new spaceborne measurement capabilities and technologies, six of which are still operating. We include two additional instruments that represent new technologies first flown in the early 2000s but updated during this period with new satellites. We have kept the instruments together for readers interested in these technologies. Each of the new satellites was designed to study environmental properties previously inaccessible or with enhanced capabilities to those previously obtained. Much of this expansion was made to address concerns about impacts of rapid climate change that for many scientific disciplines require new observations that reduce uncertainties in numerical, energy budget and radiative transfer model predictions. These concerns require expansion of measurements in the thermal infrared to address planetary heating and quantify global losses of polar and glacial ice masses. Better monitoring of soil moisture, especially for agriculture and quantifying changing patterns and losses of groundwater/aquifer storage is another critical need. Better monitoring of atmospheric chemistry, including carbon cycle components and harmful air pollutants that impact the health of the biosphere. These technologies draw upon a new generation of lidars that can survive in the harsh space environment, radar technologies with SAR and polarimeter data, and detectors to enable measuring narrow spectral bands strategically positioned at absorption features to identify atmospheric chemicals, surface water constituents, and vegetation properties.

### 5.1. Gravity Recovery and Climate Experiment (GRACE)

The **GRACE** satellite program is a collaboration between NASA and the German Space Agency (DLR) with a mission to measure the Earth’s gravity fields and gravity field anomalies (Table 7). The revolutionary two satellites in one orbital track provided the accuracy necessary for these measurements. In 2002, the original GRACE mission placed a pair of identical satellites (GRACE-1 and GRACE-2) flown ~220 km apart in tandem formation at 485 km initial altitude, but due to atmospheric drag reached 383.7 km before the end of the mission. It flew in a near-polar orbit with an 89° drifting inclination angle. Each satellite carried a highly precise **M**icro**W**ave ranging **I**nstrument system (MWI) with microwave interferometers (High-Accuracy Inter-satellite Ranging System, HAIRS) a K-band (24 GHz) and a Ka-band (32 GHz) to detect gravity anomalies. As they travel over a gravitational anomaly, an area of greater mass at the Earth’s surface will cause the lead satellite to pull away from the trailing one, while a lower mass will result in slowing the leading satellite, causing it to move slightly closer to the trailing satellite (https://gracefo.jpl.nasa.gov/resources/38/grace-fo-fact-sheet/ (accessed on 2 April 2024)). These small changes in distance can be detected to within 10 microns by the highly precise MicroWave Instrument system. A detailed map of gravity anomalies can be made by measuring the constantly changing distance between the two satellites and combining that data with precise positioning measurements from Global Positioning System (GPS) instruments. Highly accurate 3-axis SuperStar Accelerometers were located at the center of each satellite’s mass to detect accelerations caused by atmospheric drag, to isolate them from the signal due to gravity. The system also measured temperature and humidity sounding with very high vertical resolution with Blackjack GPS receivers that measure the phase delay from refraction during occultation. The GRACE program has produced monthly gravity anomaly maps and related products, primarily detecting changes in the distribution of water across the planet and revolutionized our understanding of how water moves and is stored on Earth. GRACE also made a significant contribution to monitoring groundwater [101] and temperature and humidity sounding, as well as ice sheet thicknesses (Table 7), and other changes to the Earth’s crust [102,103]. The GRACE mission lasted almost three times its expected lifespan but ended in 2017, due to declining battery capacity.

### 5.2. GRACE FOLLOW-ON (GRACE-FO)

**T**he GRACE-FO mission began in 2018, less than a year after GRACE was terminated (Table 7). The HAIRS ranging system and SuperSTAR 3 axis accelerometers were used in GRACE-FO. The GPS was changed to a triple GPS (international) with GPS, Galileo and GLONASS system. GRACE/GRACE-FO data were used to evaluate the 20-year trends in water storage obtained during the two mission phases [104]. A recent paper by [105] demonstrated how new products from GRACE/GRACE-FO could be used to develop more hydrologic parameters for studying water science. [106] described a pattern of changing intensity of hydroclimate extremes using GRACE/GRACE-FO time series data. The 2017 National Academy Decadal Survey [107] study identified mass change as a scientific priority “Designated Observable” to be flown following the GRACE-FO instrument. NASA has identified the Gravity Recovery and Climate Experiment-Continuity (GRACE-C) to be launched in 2028 to measure large-scale mass changes that will improve understanding of the global water cycle [108].

### 5.3. Aquarius/SAC-D Oceanography Pathfinder

**Aquarius** was an Earth System Science Pathfinder Program for oceanography that was launched in 2011 and operated for more than 3.5 years (Table 7). This satellite was an international collaboration between NASA and Argentina’s space agency, Comisión Nacional de Actividades Espaciales (CONAE), with participation from Brazil, Canada, France, and Italy. Aquarius flew on the Argentine SAC-D spacecraft in a sun-synchronous LEO at 657 km altitude. Aquarius’ three radiometers had antenna reflectors 2.5 m in diameter, scanning a 390 km wide swath of the ocean’s surface with 150 km GSD and a weekly repeat cycle [109]. The primary instruments were radiometers that measured microwave thermal emissions frequencies for salinity, specifically using the most accurate L-band Polarimetric Scatterometer (1.413 GHz) developed to examine ocean salinity. Aquarius was also capable of observations for land hydrologic processes, ocean temperature and humidity soundings, and for space weather [110]. Within the first month of operation, Aquarius produced the first scientifically significant salinity maps of the Earth’s oceans, showing large spatial and temporal patterns in surface salinity [111]. Salinity changes are occurring in response to climate changes that influence ocean circulation and impact the global water and energy cycles [112]. This was also the first mission to combine use of passive (scatterometer) and active (polarimetric radiometer) measurements to accurately measure salinity, which proved that variations in ocean salinity could be tracked from space [110]. Aquarius data are available from https://podaac.jpl.nasa.gov/aquarius/ (accessed on 2 February 2024).

### 5.4. NASA’s Soil Moisture Active Passive (SMAP)

**Figure 8:**
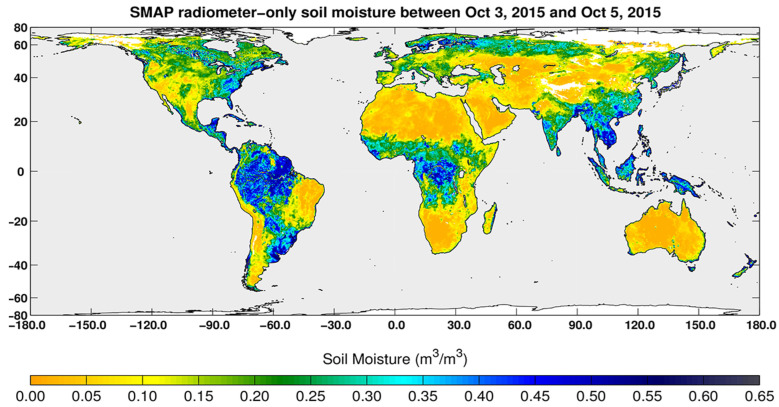
NASA’s Soil Moisture Active Passive (SMAP) satellite is producing global data. Here, a 2-day interval (3–5 October 2015) is shown for early autumn in the northern hemisphere, and late spring in the southern hemisphere). The data show high variability in soil moisture, with desert regions standing out for low values. L-band brightness temperatures obtained from the passive SMAP radiometer are used to produce these global estimates of soil moisture. SMAP data are available from the NASA National and Ice Data Center. Data are beta versions of Level-2 and Level-3 SMAP data (Credit SMAP Science Team, Jet Propulsion Laboratory, released 4 October 2015).

The NASA Soil Moisture Active Passive (**SMAP**) mission has heritage from the SMOS mission (Table 7). SMAP was launched into a polar sun-synchronous LEO orbit in 2014, originally planned as a three-year mission. Despite losing the 1.26 GHz (L band) radar due to a power failure three months after launch, SMAP has been retained in an extended operating phase since 2018 because of the successful passive SMAP 1.41 GHz (L band) radiometer, which has been collecting flawless data (https://smap.jpl.nasa.gov/mission/description/ (3 February 2024)). The radar was the intended to provide a “high spatial resolution” instrument with a ground footprint of 1–3 km, while the passive microwave’s ground footprint is 30 km × 47 km (https://smap.jpl.nasa.gov/ (3 February 2024)). SMAP’s mission was to collect a near surface global ‘snapshot’ of soil moisture and freeze/thaw states to better understand terrestrial water, carbon, and energy cycles [113,114] (Figure 8). The microwave instrument acquires global coverage every two days near the poles and up to three days near the Equator.

The paper by Watts et al. [115] documented 12 years of SMAP data to demonstrate that CO_2_ uptake dominates the carbon budget in high latitude Eurasian ecosystems. [102] used a coupled permafrost hydrology and SMAP microwave emission model to simulate changes in the L-band data during the thaw season over the Alaskan North Slope. Derksen et al. [116] demonstrated soil moisture dynamics could be accurately retrieved from SMAP active and passive radar instruments in agreement with ground data, providing key information on patterns of permafrost thaw and vulnerability to climate warming. Applications of freeze/thaw information guide decisions on when to time agricultural production [117]. Soil moisture directly impacts the fluxes of CO_2_ and methane (CH_4_) in biospheric/atmospheric exchanges in the terrestrial carbon cycle. Better soil moisture estimates improve the coupled carbon–water model estimates of large-scale carbon fluxes and climate feedbacks [118,119]. The study by Zhang et al. [120] shows that directly inserting SMAP data into a coupled carbon–water dynamics terrestrial ecosystem model promises to significantly reduce model errors and improve performance.

### 5.5. Surface Water and Ocean Topography (SWOT)

**SWOT** is a joint mission of the CNES and NASA, with additional contributions from the UK and Canada (Table 7). It is the first global survey to measure the Earth’s surface waters, both land and marine, and observe how they change over time [121]. A second goal is to observe the fine structures of the ocean’s surface topography. This mission was first identified as a scientific priority in the 2007 National Academies of Sciences, Engineering and Medicine’s Decadal Survey of the Earth Science missions [122] NASA should implement within the decade. The mission finally entered the Preliminary Design and Technology Commission in 2015 and the Final Design and Fabrication state in 2016. It was launched in 2022 for a three-year mission that will likely be extended. SWOT (Table 7) has a 21 day repeat cycle in a drifting orbit. The primary instrument is a Ka Radar interferometer (KaRin) built by the Jet Propulsion Laboratory and a GPS receiver; the Canadian Space Agency provided the power assembly. CNES built the Doppler Orbitography and Radiopositioning Integrated by Satellite (DORIS) antenna that will pick up ground-based radio beacons distributed across the Earth. A nadir Jason class altimeter collects data in the gap between KaRin swaths that will determine the sea-level height. A Microwave Radiometer measures water vapor between the SWOT and the surface to correct for slower radar signals. This combination of measurements will allow precise surface height data.

### 5.6. Global Precipitation Mission

**NASA’s** Global Precipitation Measurement (GPM) mission is a joint NASA and JAXA mission (Table 7). It began with the February 2014 launch of its core GPM observatory from the Tanegashima Space Center in Japan into an inclined 65° drifting orbit at a relatively low altitude of 398 km but was later raised above the ISS to ~442 km on 7–8 November 2023 (Table 7). It is planned to operate until 2030 [123,124]. The GPM observatory maps global precipitation every three hours to provide next-generation global observations of rain and snow, including raindrop sizes, and serves as the reference for a consortium of international space agencies. These include CNES, ISRO, NOAA, EUMETSAT, and others. The mission builds upon the success of the earlier **T**ropical **R**ainfall **M**easuring **M**ission (TRMM, 1997–2015), also a NASA-JAXA collaboration. 

The GPM satellite’s two instruments are NASA/Goddard’s passive multichannel GPM Microwave Imager (GMI) and JAXA’s Ku/Ka Dual-frequency Precipitation Radar (DPR), the first placed in space. The conically scanning GMI radiometer covers an 885 km swath with thirteen channels (frequencies, 10 to 183 GHz) optimized to retrieve heavy, moderate, and light precipitation values based on the polarization difference per channel which can be related to cloud optical thickness and water content. The DPR instrument uses precipitation radars with a Ka-band (35.5 GHz, KaPR) and a Ku-band (13.6 GHz, KuPR) to provide three-dimensional precipitation structure measurements. The DPR originally collected radar data over different swath widths of 125 km (Ka band) and 245 km (Ku band), but since 2018 both swaths are 245 km. 

**Figure 9:**
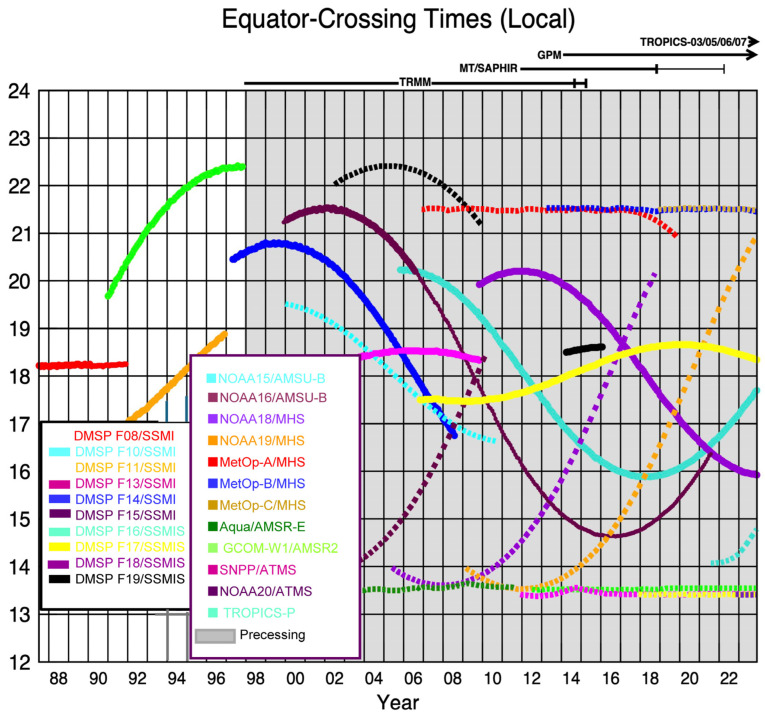
The changing time of equatorial overpasses through the years, shown on the Y axis as the hours between noon and midnight (the afternoon/evening overpasses of the ascending pass of the daily circuit) for environmental monitoring satellites included in the consortium supporting the Global Precipitation Mission (GPM). Left legend column, all DMSP missions, Right column, missions are mixed and identified. Ascending passes shown (F08 and TROPOCS-PF descending); satellites depicted precession throughout the day. Figure adapted from Eric Nelkin (SSAI), 12/20/2023, NASA/GSFC, Greenbelt, MD. The consortium supporting the Global Precipitation Mission (GPM). Left legend column, all DMSP missions, Right column missions are mixed and identified. Ascending passes shown (F08 and TROPOCS-PF descending); satellites depicted precessing throughout the day. Figure adapted from Eric Nelkin (SSAI), 12/20/2023, NASA/GSFC, Greenbelt, MD. https://gpm.nasa.gov/missions/GPM/constellation (accessed on 1 April 2024).

The GPM mission is advancing our understanding of Earth’s global water and energy cycles, which has improved forecasting of extreme events and provides timely precipitation information of direct benefit to society (Figure 9). The GMI mission is designated as the first realization of the CEOS Precipitation Virtual Constellation The constellation of satellites that have contributed to the GPM mission include Suomi NPP (NASA/NOAA), MetOP A/B/C (EuMETSAT), NOAA 18/19/20 (NOAA), GCOM-W1 (JAXA, A-Train), Megha-Tropiques (CNES/ISRO), and DMSP F17/F18 (US DoD). In 2002 the United Nations Program on “Remote Sensing for Substantive Water Management in Arid and Semi-Arid Areas” identified the GPM Mission as an outstanding example of peaceful use of space. Now in its tenth year, the mission is operating normally with some minor issues, and a final updated Mission Operations Plan is underway.

### 5.7. NASA’s First LiDAR Mission: ICESat

ICESat was the first lidar mission in NASA’s Earth-Observing System (EOS). It was designed to measure ice sheet mass balance, clouds, and aerosol heights, as well as land topography and vegetation characteristics (https://icesat.gsfc.nasa.gov/icesat/glas.php/ (accessed on 6 February 2024)). It flew for more than six years from 2003 to 2010 and was the first laser-ranging (lidar) instrument in space (Table 8) making continuous global observations of topography, ice, snow, and land. ICESat provided extensive coverage over the Greenland and Antarctic ice sheets. The mission demonstrated that providing multiyear elevation data are needed to resolve ice sheet mass balance, and to characterize the stratospheric clouds that are common over polar areas.

The only instrument on ICESat was the **G**eoscience **L**aser **A**ltimeter **S**ystem (**GLAS**), which provided three lasers: a precision surface lidar, a sensitive dual-wavelength cloud lidar, and an aerosol lidar. Its three lasers operated at 40 pulses/s to illuminate ground footprints (70 m diameter) spaced at 170-m intervals along its flight path (https://earthobservatory.nasa.gov/features/ICESat/ (accessed on 18 February 2024)), with a 91-day repeat cycle for most of its mission. Distance was measured using a GPS receiver and a star-tracker to determine the attitude (https://icesat.gsfc.nasa.gov/icesat/glas.php/ (accessed on 6 February 2024)). The three laser transmitters on GLAS sent short pulses (4 nanoseconds) of IR light at 1064 nm wavelength, alternating with pulses of visible green light at 532 nm, over water or through clouds. The receiver included sensitive photon counting detectors in nadir or limb operation, probing the vertical distribution of clouds and cloud properties. Photons that are reflected back to the spacecraft from the surface of the Earth and from the atmosphere were collected in a 1 m diameter telescope. Applications for ICESat include mapping vegetation height [125,126], mapping of Arctic sea ice [127] and Antarctic sea ice [128].

### 5.8. Ice, Cloud and Land Elevation Satellite (ICESAT-2)

The NASA ICESAT-2 was launched in 2018 for a three-year mission (Table 8), with fuel for seven years, that, due to its successful operation, was extended in 2022 for three more years, now to EOL ≤ 2026 (space.oscar.wmo.int/satellites/view/icesat_2/ (accessed on 8 February 2024)). Its mission is to continue assessment of polar ice changes and measure vegetation canopy height over land [129]. Vegetation height measurements are intended to enable estimates of biomass and aboveground carbon in forests (https://database.eohandbook.com/database/missionsummary.aspx?missionID=613/ (accessed on 9 February 2024)). It flies in a drifting orbit at ~481–495 km and a 94° inclination angle, higher than the International Space Station.

The Advanced Topographic Laser Altimeter System (ATLAS) instrument is a more capable altimeter than the one used on the first ICESat mission [130]. It can measure ice sheet changes as small as 4 mm/yr. Over land, ATLAS can measure ice height changes of 0.25 m/year for areas as large as 100 km^2^. It can measure vegetation height to 3 m for monthly averages, from observations spaced ~1 km. Backscatter from aerosols and clouds can be measured in a vertical column from 14 km altitude to the surface, with 300 vertical bins resolution; along-track resolution is 280 km.

**Figure 10:**
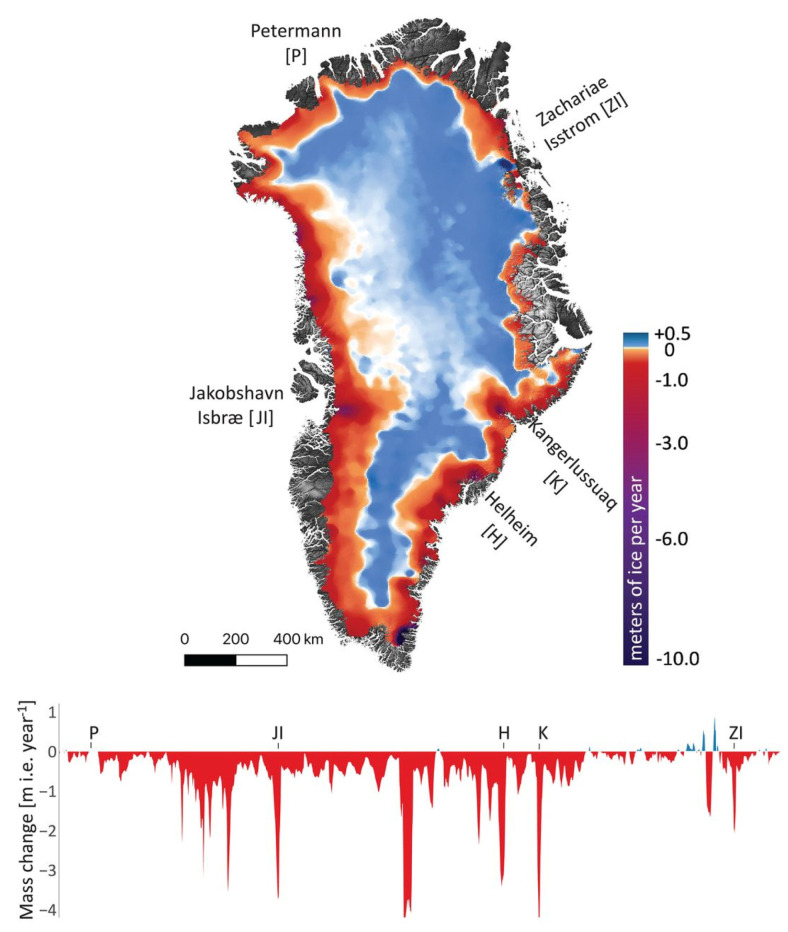
Changes in the Greenland ice sheet between 2003 and 2019, as measured by ATLAS on ICESat-2. Greenland’s interior is still gaining ice mass but its coastal edges are becoming ice free and the width of the retreating ice flow is wider. The color bar on the right shows ice gain in blude and loss in increasingly dark shades of red. The bottom panel shows mostly declining ice mass at different locations around the perimeter. The map has been smoothed with a 35 km filter to improve visualization. Reproduced with permission from Smith et al. [131].

ATLAS is a photon counting lidar that measures the travel times of laser pulses to calculate the distance between the spacecraft and the Earth’s surface. ATLAS hosts two lasers, a primary and a backup; measuring at a 332 nm wavelength, it sends 10,000 pulses/second compared with ICESAT’s rate at 40 pulses/second. ATLAS has a multipulse multibeam design with six beams, arranged in pairs. With this information density, it can estimate the slope of the surface it flies over. It measures enough height data to estimate the annual elevation change in Greenland and Antarctic ice shelves (Figure 10), measuring high sloped areas and crevasses, even when changes are as little as four millimeters, and allowing it to measure every 0.70 m along the ground path. 

ATLAS and GLAS have been used to measure lake levels and estimate water storage [132]. ATLAS has a 0.8 m diameter beryllium telescope that is designed to align with the returning photons. Approximately 20 trillion photons are emitted from the system but only a small fraction (approximately 12) are reflected to the telescope and are captured, but this is many more than was achieved by ICESAT. Once a returning photon is detected, it triggers the detector, and the timer that started when the pulse was emitted is stopped. The distance to the ground object is calculated by half the distance the photon traveled. At the satellite elevation it takes approximately 3.3 milliseconds, +/−1 millisecond to pass to the Earth and back to the satellite (https://www.eoportal.org/satellite-missions/icesat-2#mission-capabilities/ (accessed on 9 February)).

One of the primary missions of ICESat-2 is to measure the height of ice sheets and changes in ice cover and height over time that indicate growth or loss of ice mass, e.g., [133,134]. ICESat-2 has a 532 nm wavelength lidar, which measures a 13 m ground footprint. Among the environmental applications are measurements of forest height and structure, e.g., [135], forest canopy biomass and carbon storage, river and stream course patterns and lake and reservoir surface heights, e.g., [132], sea-level rise for hydrologic studies, and changes in height and 3D shape of volcanos, e.g., [136]. Reported vertical resolution of vegetation canopy height is 3 m at 1 km^2^ spatial resolution. While this may seem fairly coarse, by repeated observations the ATLAS can detect changes in surface elevation as small as 0.25 m/yr over areas of 100 km^2^ (https://www.eoportal.org/satellite-missions/icesat-2#mission-capabilities/ (accessed on 9 February 2024)).

### 5.9. NASA’s Orbiting Carbon Observatory-2 (OCO-2)

The **OCO-2** is NASA’s first EO satellite to study the spatial and temporal patterns of atmospheric carbon dioxide by making direct full-column measurements of CO_2_ concentrations space from TOA [137,138]. The first OCO failed (2009) when it did not reach orbit. OCO-2 (and OCO) were designed to measure the concentrations and variable hemispheric distributions of CO_2_ in the atmosphere (Table 8) and to follow seasonal changes and geographic distributions in the CO_2_ concentrations, allowing better understanding of their regional sources and sinks. The OCO-2 was launched in 2014 for a two-year mission but is still flying today [139]. OCO-2 flies in a polar LEO orbit, with an equatorial crossing time of 13:35 in a descending orbit that repeats at 16-day intervals. This time of day is when the solar zenith angle is highest, maximizing the energy for measurements and when CO_2_ measurements are near their daily average (between late night high values and mid-afternoon minimum CO_2_ concentration values). It is in loose formation with the few remaining A-Train instruments.

**Figure 11:**
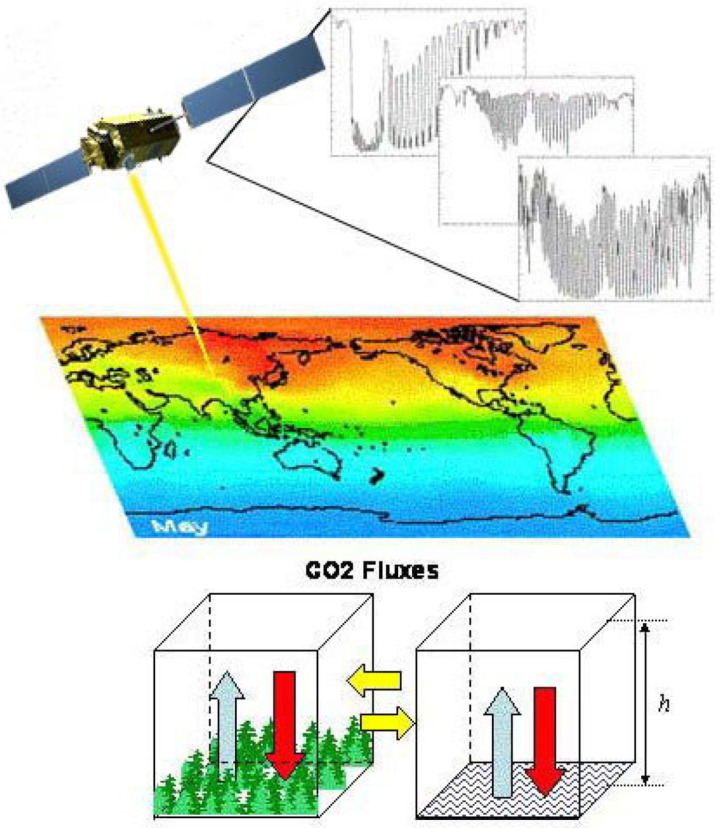
The Orbiting Carbon Observatory-2 (OCO-2) was designed to measure carbon dioxide in the atmosphere, leading to better understanding of global source and sink patterns of carbon molecules. As the satellite flies over the land, its three grating spectrometers, each with 1024 spectral channels measure the wavelength regions related to the weak and strong CO_2_ absorptions determined at the O_2_-absorption bands centered at 0.765 µm (upper panel), 1.61 µm (middle panel) and 2.06 µm (lower panel), making about 72,000 spectral soundings for each measurement to provide a strong signal to estimate the CO_2_ fluxes. The data are validated against measurements made on vegetated and non-vegetated sites (CREDIT: OCO-2 Program site, https://ocov2.jpl.nasa.gov/science/measurement-approach/ (accessed on 10 March 2024)).

The OCO-2 carries a single instrument that has three high-resolution grating spectrometers (Figure 11) that make soundings, each covering a different spectral region (https://ocov2.jpl.nasa.gov/observatory/instrument/ (accessed on 9 February 2024)), chosen to measure different wavelengths where CO_2_ absorbs. Each sample represents an area of ~3 km^2^ in the nadir view and each instrument can gather up to 72,000 soundings on the sunlit descending orbits. With this density of soundings OCO-2 can estimate CO_2_ concentrations even in the presence of clouds and aerosols, and areas of topographic roughness. O_2_ is separately measured at the oxygen-A band (around 0.76 μm) and used to test against CO_2_ measurements in the weak CO_2_ feature (~1.61 μm) and the strong CO_2_ absorption (~2.06 μm), since it is relatively uniformly distributed through the atmosphere. The weak CO_2_ band is most sensitive to the CO_2_ concentrations near the surface and provides a clear signal since other atmospheric gases do not absorb in this wavelength region [140]. The 2.06 μm band is an independent measure of the concentration of CO_2_ in the atmosphere and is very sensitive to the presence of aerosols in the atmosphere, and to variations in pressure and humidity.

### 5.10. Second-Generation Atmospheric Chemistry: Sentinel-5P

Following successes from SCIAMACHY on Envisat, GOSATs-1 and -2 from JAXA, and OCO-2, there has been increased interest in developing satellite instruments to quantify and locate sites for emissions of climate warming atmospheric trace gases, aerosols and other atmospheric chemicals of concern for impacts on the Earth’s radiation budget or for lower tropospheric gases, impacts on planetary health (Table 8). The Copernicus Sentinel-5 Precursor (P) mission Sentinel-5P has direct heritage from SCIAMACHY and was co-funded by ESA and the Netherlands: The Royal Netherlands Meteorological Institute, KNMI; the Space Research Organization Netherlands (SRON); and the Netherlands Organization for Applied Scientific Research (TNO). The Sentinel-5P was launched in 2017 for a seven-year mission [141], currently extended to 2027, to monitor air pollution with an extensive suite of atmospheric trace gases and aerosols, including ozone, methane, carbon monoxide, formaldehyde, nitrous dioxide and sulfur dioxide [142]; these trace gases also contribute to climate change.

The S-5P flies in a sun-synchronous ascending polar orbit at 824 km with equatorial crossing time at 13:30, and carries the TROPOspheric Monitoring Instrument (TROPOMI), a passive grating nadir viewing imaging spectrometer. TROPOMI operates in push-broom mode and has a wide swath of 2600 km (108°) for full daily coverage of radiance and reflectance (https://sentinels.copernicus.eu/web/sentinel/missions/sentinel-5p/satellite-description/ (assessed 8 January 2024)). Spectrometer measurements are made in narrow bands grouped in wavelength intervals of UV1 (270–300 nm), UV2 (300–370 nm), VIS (370–500 nm) NIR1 (685–710 nm) NIR2 (745–773 nm), and SWIR1 (1590–1675 nm) and SWIR2 (2305–2385 nm) spectral regions, described in Sentinel Online at (https://sentinel.esa.int/web/sentinel/missions/sentinel-5#:~:text=The%20Sentinel%2D5%20mission%20consists,3%20(2305%2D2385n/ (8 January 2024)). S-5P has five hyperspectral sections at wavelengths from UV to SWIR that are used to measure atmospheric chemistry and weather observations. It has a cross track spatial resolution of 3.5 km × 7 km for UV2, VIS and NIR bands, with larger pixels for the UV1 band (7 km × 28 km) and SWIR bands (7 km × 7 km) (https://sentinels.copernicus.eu/web/sentinel/missions/sentinel-5p/orbit/ (accessed on 13 January 2024)).

The Sentinel-5P is flying in tandem following the VIIRS instrument on the Suomi NPP mission (see Suomi NPP for more details in Section 7.1.1) by 3.5 min. The wide swaths of the VIIRS on Suomi NPP (3000 km) and the S-5P (2600 km) allow significant overlap between the area viewed by these instruments (Table 8). This provides opportunities to link atmospheric chemistry measurements to the ground surface conditions and physiological processes measured by VIIRS, e.g., [143,144,145]. The configuration of TROMPOMI allows using the VIIRS data to provide a high-resolution cloud mask during routine processing of the TROPOMI methane product. Applications include monitoring volcanic activity [146] and NO_2_ over oil sands [147]. Sentinel-5P has reduced the temporal data gaps that developed between the 2012 termination of the SCIAMACHY mission and the OMI/AURA mission, as well as with the future Sentinel-4 and Sentinel-5 missions [148,149]. Approximately 1 TB of S-5P data are downloaded daily, so this satellite is a precursor for the much larger data volumes coming in this decade.

During the first decade of 2000, the hyperspectral spaceborne observations made by ESA’s CHRIS-PROBA and SCIAMACHY, and those acquired by NASA’s Hyperion imaging spectrometer on the Earth Observer-1 (EO-1) satellite [81,150], were an incredible success for land-based science. S-5P is the mission that comes out of this heritage and demonstrates the merits (as a precursor) for the future launch of the Sentinel-5. The S-5P, when coupled with the Global Ozone Monitoring Experiment (GOME-2) flown on METOP (2006/2026, Table 5) and linked with previous measurements by the Ozone Monitoring Instrument (OMI)/and the SCIAMACHY mission on Envisat (previously described, Section 3.5, Table 4), together provide a several decades-long record of diurnal observations of atmospheric gases and aerosols.

## 6. ESA’s Modern Research Satellites: The Earth Explorer (EE) Program

The Earth Explorer (EE) program instruments are one-off pioneering science missions that are designed to advance critically important new scientific measurements identified by the EO community. These missions may be riskier than operational satellites since they are pioneering new technology, or parts of the mission are pioneering new components or methods that propose to solve complicated challenges in ways not previously tested in space. If the missions are successfully demonstrated and produce valuable data products, they might become future Sentinel missions. Nine EE missions have been approved, with four not yet flown (EEs 6–9). Two Earth Explorers have completed their missions and accurately mapped the Earth’s geoid (EE-1 GOCE and EE-5 Aeolus). The EE-1 GOCE (Gravity field and steady-state Ocean Circulation Explorer, 2009–2013), mapped the deep structure of the Earth’s mantle, probed hazardous volcanic regions (Table 9). The GOCE data, combined with sea-surface height data from other instruments has been used to map geostrophic ocean currents. EE-5 Aeolus was the first satellite to measure global wind profiles that provided important data for weather forecasting (Table 9). It was the first wind-profile observer from space, making novel advances in laser technology, exceeding expectations. It was launched in 2018 and ended in 2023, but its success paved the way for a future mission (Aeolus-2) to measure wind fields.

Three EEs are currently in orbit (Table 9): EE-2 SMOS (2009–2025), EE-3 CryoSat (2010–2025) carrying a SAR interferometric radar altimeter (SIRAL) to measure sea-surface levels, and the EE-4 SWARM, a constellation of four satellites (SWARM A, SWARM B, SWARM C, SWARM D) launched into polar orbit in 2013 and 2018, with extensions to operate through 2025. The objective of SWARM is to measure the Earth’s gravitational field and the electric field of the atmosphere.

The sixth, seventh, and eighth Earth Explorer missions with near-term launches will be discussed in a later section (Section 13.1,Section 13.2,Section 13.3 and Section 13.4): EarthCARE (EE-6), Biomass (EE-7), the Fluorescence Explorer, FLEX (EE-8), and FORUM (the Far-infrared Outgoing Radiation Understanding and Monitoring mission, EE-9), are planned for launches between 2024 and 2027. The tenth Earth Explorer, Harmony is a passive radar and thermal mission, is currently undergoing feasibility studies and has a proposed launch in 2029.

### 6.1. Earth Explorer-5 Atmospheric Dynamics Mission—Aeolus

Aeolus (2018–2023) was an experimental EE-5 mission to map global wind fields from the near-surface up to 30 km altitude using a novel Doppler Wind Lidar method to observe wind profile observations (Table 9). The Atmospheric Laser Doppler Instrument (ALADIN) was a Direct Detection Doppler Lidar [151]. It generated and emitted pulses of UV (355 nm) down its Cassegrain telescope through the atmosphere, oriented 35° from nadir and 90° to the satellite track (away from the sun). This allowed it to transmit and receive light perpendicular to the direction of the satellite, allowing ALADIN to determine the east–west horizontal component of the winds [152]. When the UV pulses interacted with particles in the atmosphere, the light scattered along the line-of-sight path within its narrow field of view. Two receivers (Mei and Rayleigh) on the instrument measure the Doppler shift of the backscattered signal with respect to the frequency of the pulse as a function of time [153]. Scattered small gas molecules become a Rayleigh signal and aerosols, ice crystals and water droplets become the Mei signal. Overall, the mission was highly successful and is leading the way for a second Aeolus-2 mission and others. This atmospheric scattering data it recorded has improved our understanding of atmospheric dynamics and weather forecasting. The data have had its greatest impact on weather prediction in the tropics, southern hemisphere, and polar regions where there are few direct measurements of winds [153,154].

### 6.2. Earth Explorer-2 (EE-2) Soil Moisture and Ocean Salinity (SMOS)

SMOS is a pioneering program to improve understanding of the Earth’s water cycle [155], the continuous exchange of water between the oceans, atmosphere, and land (https://earth.esa.int/eogateway/missions/smos (accessed on 5 January 2024)). And how variations in temperature and salinity affect ocean currents (Table 9). In the Arctic, SMOS measures temperature, total sea ice extent and concentration, ice thickness and volume, and timing of sea ice advance, retreat, accumulation, and melt season lengths (earth.esa.int/eogatewat/missions/smos/description).

The SMOS instrument is the Microwave Imaging Radiometer using Aperture Synthesis (MIRAS) (https://earth.esa.int/eogateway/instruments/miras/ (accessed on 5 January 2024)) a two-dimensional interferometric L-band (21 cm, 1.4 GHz) radiometer in the full polarimetric mode (the radar can transmit and receive pulses in horizontal (H), vertical (V) and in the cross-pole orientations (i.e., HV, sending in H/receiving in V; or sending in V/receiving in H, VH). The HH, VV, HV, VH full polarimetric measurements are the default since 2010. Typically, a stronger signal is returned if the reflecting object is oriented in the same direction as the pulse wave. Thus, the single radar band provides two (dual polarization) or four (full-polarization) types of information about the objects [156,157]. At L-band frequency the atmosphere is mostly transparent and surface emissions are strongly related to soil moisture over the land surfaces and salinity over the ocean surface, and to thickness of sea ice (https://www.ecmwf.int/en/research/projects/smos (accessed on 5 January 2024)). SMOS measures salinity to 0.1 practical salinity units (~0.1 parts per thousand, ppt) averaged over a 10–30-day measurement period for an area of 200 km × 200 km [158]. It uses an **I**nterferometric **S**ynthetic **A**perture Radar (InSAR) technique that superimposes two images acquired from different optical paths and the location differences create interference fringes that can be viewed as an image, termed an interferogram. 

**Figure 12:**
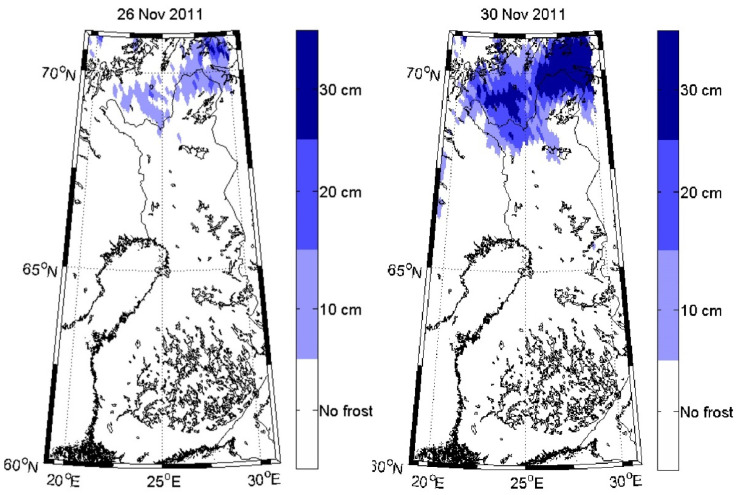
Soil Moisture and Salinity (SMOS) data provide information on the extent and changes in frozen soil at a nearly daily time step. In this pair of imagers, at the start of the annual freeze up period in northern Finland (26 November and 30 November 2011). In just four days, there is a large increase in the cover of frozen soil. Finnish meteorologists developed a method to infer the depth of the frozen layer, based on an increase in brightness temperature that stops increasing when the top 50 cm is frozen (figure credit: https://www.esa.int/Applications/Observing_the_Earth/FutureEO/SMOS/SMOS_detects_freezing_soil_as_winter_takes_grip/ (access 18 May 2024)).

The winter freeze up can happen quickly in northern regions. Figure 12 shows the start of winter freeze-up in northern Finland. Freezing had just started on 26 November 2011 as seen in the image, but the frozen area had significantly expanded four days later on 30 November 2011. The knowledge of the patterns of freeze-up and spring thaw are critical to understanding potential release of methane from polar wetlands. But advance or early warning could be important for safety issues during these transitional seasons, e.g., flooding after rapid ice melt.

The CryoSat-2 mission was designed to measure the thickness of polar and marine sea ice and to monitor dynamic changes in ice sheet thicknesses (Table 9). The mission launched in 2010 and will end in 2025. It is dedicated to monitoring the thinning rate of Arctic sea ice from global warming, but it applies to Greenland, Antarctica, and Antarctic sea ice. These data will provide essential information on fluctuations in the mass of the Earth’s major land and marine ice fields at seasonal and inter-annual time scales and estimate contributions of Antarctic and Greenland ice sheets to global seal level rise. CryoSat-2’s geocentric orbit enables it to observe ice close to the poles, reaching 88° N.

CryoSat-2 has a precision radar altimeter, the SAR Interferometer Radar Altimeter (SIRAL) to measure land surface topography [159,160], sea ice thickness and sea ice sheet topography [161] and the global gravity field [162]. It has heritage from Poseidon-2 on the CNES-sponsored Jason-1 mission (in 2001). It uses a Ku-band SAR with a pulse bandwidth of 320 MHz, operating in the high- and low-spatial resolution modes, the later used in conditions of slow change such as interior ice sheets. The SAR mode is used over ice and smaller ice flows to extract Doppler properties. The interferometric mode (SAR-n) is used along ice sheet margins and small ice caps, mountain glaciers, and along geotrophic ocean currents and major hydrological basins. 

In addition to the SAR measurements, CryoSat-2 obtains precise real-time orbit determination from the Doppler Orbitography and Radio-positioning Integrated by Satellite (DORIS), which measures the Doppler shift in VHF and S-band signals transmitted by ground beacons at 2.03625 GHz and 401.25 GHz. DORIS uplinks the precise orbit information needed to interpret the altimeter data and overall performance. It also has a Laser Retro-Reflector (LRR) a passive optical device that provides precise orbit determination with the aid of the international laser tracking network. The Sentinel Expansion **CRISTAL** mission (**Sentinel-9**) is scheduled to replace CryoSat-2 in 2028 [163].

### 6.3. Earth Explorer-4 SWARM

The Earth’s Magnetic Field and Environment Explorer-A (SWARM) is a constellation of three satellites (SWARM A, SWARM B, SWARM C), comprising ESA’s Earth Explorer-4 (Table 9). Each SWARM was launched into a polar orbit in 2013, with initial mission extensions to operate until 2025 [164,165]. Two Swarm satellites fly side-by-side at 462 km altitude and 87.35° inclination angle, and the third flies at a higher, 511 km altitude at 87.75° inclination to provide a three-dimensional field reconstruction. The differences in altitude and inclination angle result in SWARM B slowing drifting away from SWARM A and C at 24°/year (https://earth.esa.int/eogateway/missions/swarm#instruments-section/ (accessed on 23 February 2024)). The objective of the SWARM mission is to measure the Earth’s geomagnetic and electric fields and contribute to understanding core dynamics and core–mantle interactions, understanding the magnetization of the lithosphere, electrical conductivity of the mantle and currents flowing in the magnetosphere and ionosphere.

The SWARM satellites have a seven instrument package for data measuring solid earth and Space Weather properties, including a core measurement from the Vector Field Magnetometer (VFM) to measure magnetic field vector components; the Absolute Scalar Magnetometer (ASM, from CNES and the French Atomic Energy Commission) to calibrate the VFM; the electric field instrument to measure ion density, drift velocity and electric fields; an accelerometer to measure non-gravitational air-drag; and a Laser Retro-Reflector with reflecting quartz prisms to participate in the laser ranging network. In 2013, the Canadian Space Agency’s CASSIOPE (**C**ascade, **S**mall**S**at and **IO**nospheric **P**olar **E**xplorer) became the fourth satellite as SWARM E, a 3rd-party designation in the SWARM series. CASSIOPE is Canada’s first hybrid satellite, supporting dual missions in scientific research of space weather [166], with an enhanced Polar Outflow Probe (“e-POP”), and high-speed telecommunications, but it was retired in 2021 when it lost it second momentum wheel and the spacecraft could not be controlled (https://epop.phys.ucalgary.ca/routine-cassiope-swarm-echo-science-operations-come-to-an-end/ (assessed 26 February 2024)).

## 7. The Operational Satellites

Until the designation of Landsat-8 as an operational satellite, all operational satellites were weather satellites. Operational missions are implemented from the heritage of well-established measurement types and protocols and often a primary focus is on “data continuity” rather than new types of data. These instruments do change as technology improves (e.g., the AVHRR series in Table 2), the transition from AVHRR and MODIS (Table 4) and to VIIRS (Table 10). A theme of recent advances for operational missions has been to provide higher spatial resolution data and more frequent satellite overpasses, a community desire since the earliest days of Landsat. By the early 2000s, satellites described earlier in this paper were starting to measure new spectral regions and provide higher spatial resolution and temporal resolution. These new satellites use emerging technologies and spectral band inclusion beyond the VNIR imagers of the early satellites, supplemented with one or two bands of TIR. Operational satellite programs are supported by both the USA (NASA, USGS, NOAA) and Europe (ESA, EUMETSAT). While these instruments are operational (producing reliable data from mature technologies), the science applications that these data address is extremely broad and growing as interest in using these satellite instruments to address previously unanswerable questions continues to expand into new scientific territory. In contrast, the experimental research satellites are flown to test new ideas or advance science with more capable instruments. Instead of 5-, 7-, or 10-year missions, the research satellites have 1, 2 or 3 years to demonstrate success in applications of new measurements. If the missions are judged to be collecting important useful data, and they meet their threshold or baseline data requirements, their missions are often extended, especially if that capability is considered for a future operational mission.

### 7.1. Operational USA Polar-Orbiting Satellites

#### 7.1.1. Landsat’s Operational Multispectral Imagers

Landsat-8 and Landsat-9 (NASA/USGS). The Landsat program initiated its operational program with the advent of Landsat-8 (L-8) in 2013, followed by its duplicate, Landsat-9 (L-9) in 2022; these are currently the most widely used moderate-resolution multispectral (MS) satellites (Table 10). L-8 and -9 replaced their earlier instruments with two more advanced, upgraded instruments, the Operational Land Imager (OLI) and the Thermal Infrared Sensor (TIRS). Since 2022, with 180° offset orbits, these two Landsat MS imagers together provide global eight-day coverage. Together, Landsats-5, L-7, L-8, and L-9 provide the longest existing record of (1984–2024) global sub-monthly moderate-resolution (30 m) environmental monitoring (Table 10).

Until L-8, neither complete pole-to-pole strips nor complete continental acquisitions were undertaken due to down-link (and other) limitations. Earlier satellites in the series had evolved to provide more spectral bands and smaller 30 m^2^ pixels. The data archives stored at USGS from Landsats-5 and 7 provide an extended 39 year overlapping data record for time series studies when combined with the current L-8, L-9 that provides changes observed at spatial scales relevant to understanding local changes in vegetation, soils, and water bodies, geology, and topography [167,168].

L-8 began a new Landsat series that utilized several new technologies after successful demonstrations by the Advanced Land Imager (ALI) [169] flown on NASA’s Earth Observer-1 (EO-1) technology demonstration satellite (2000–2017), originally flying in formation one minute behind L-7. New advances included a push-broom sensor and a four-mirror telescope with 12-bit quantization. The new images have the same 185 km × 185 km images as L-5 and L-7, all linked to the World Reference System but they now are contiguous, extending 2752 km from pole to pole (Landsat Data Continuity Mission, NASA Goddard Space Flight Center).

Landsats-8 and -9 include four new bands, compared to L-7, with a total of 11 spectral bands, including a higher spatial resolution VIS panchromatic (pan) band (band 8) at 15 m GSD and a second TIRS band (band 11) at 100 m; they also include two new 30 m calibration bands: band 1 in the blue visible spectrum, useful for detecting aerosols, and band 9 in the NIR for detecting cirrus ice crystal clouds. Additional upgrades include an average acquisition of 725 scenes per day (as compared to 438 scenes/day by L-7) with improved 12 m cartographic accuracy.

In the 10 years of L-8’s operation, its data have been used to advance understanding for a wide array of environmental science needs, for latitudes ± 84°. The overlap of scenes near the poles from adjacent, sequential orbits has increased scene acquisitions near the poles, making it possible to map and track polar ice sheets, e.g., mapping Greenland [170,171], glacial lakes [172,173,174], sea ice [170], glaciers [175,176], tidal glaciers [177,178,179,180], with greater accuracy. Mapping aquatic systems and shallow coastal waters also benefitted from the improvements made to L-8 and 9’s radiometric range.

Today, the Landsat mission’s unique ~50-year record of global observations is highly valued for documenting the extensive environmental changes that occurred since the mid-20th century, rendered by climate change, habitat loss, and urbanization. The Landsat program’s history, accomplishments, and legacy have been described in detail in several recent papers [1,167,168], highlighting its emphasis on mapping applications for agriculture, forestry, fisheries, geology, and many other ecological and/or environmental applications. The Landsat satellites, and other multispectral imagers that became available over the intervening decades with a small number of spectral bands (less than 10) continue to provide workhorse technologies and datasets for achieving priority science and application goals. As of July 2023, Google Scholar listed 814,000 publications that cite Landsat, a record far beyond those of any other EO satellite [181].

#### 7.1.2. VIIRS (Visible Infrared Imaging Radiometer Suite)

The polar-orbiting LEO weather satellites provide global-scale daily or near daily data of Earth conditions (Table 10). Their monitoring capabilities were enhanced with the addition of the VIIRS instrument [182], one of five instruments, to the NASA/NOAA operational bridging mission, the Suomi NPP (National Polar-orbiting Partnership) satellite (Table 10). The Suomi NPP was launched in 2011 into an afternoon orbit ~13:30 at an altitude of 830 km, now extended until 2025. Named for Verner E. Suomi, a pioneering meteorologist from the University of Wisconsin, Madison. Suomi NPP is the first of a new generation of polar-orbiting weather satellites, providing Earth Science data about clouds and the atmosphere with these instruments: CERES (Clouds and Earth’s Radiant Energy System), OMPS (Ozone limb and nadir, Mapping Profiler Suite), ATMS (Advanced Technology Microwave Sounder), and CrIS (Cross-Track Infrared Sounder).

In 2017, the Copernicus Sentinel-5P (precursor) satellite with similar instruments was launched to follow by 3.5 min in loose formation with the Suomi-NPP, with most of the wide swaths of the two instruments overlapping (Table 8). This coordination allows joint products with the two OMPS ozone profilers that include vertical integration of total column carbon monoxide and the UV aerosol index, as well as total column ozone measurements (1 km resolution). These have shown high levels of agreement between the troposphere and the lower mesosphere [143].

The VIIRS is a whisk-broom radiometer with a swath of ~3060 km and nadir spatial (pixel) resolution of 375 m, and daily global coverage (https://space.oscar.wmo.int/instruments/view/viirs/ (accessed on 16 October 2023)). The VIIRS instruments have five Image (I) bands and a day/night band, as well as eight mid-IR and four TIR bands (with one mid-IR and one TIR band with 375 m GSD) primarily for weather observations [183]. The VIIRS has 16 (M, moderate resolution) bands, for which nadir views are collected at 750 m GSD. VIIRS has direct heritage from NASA’s MODIS imagers, but with fewer bands, 22 VSWIR and TIR bands (Table 10), compared to 36 on MODIS (Table 5) (https://modis.gsfc.nasa.gov/ (accessed on 18 October 2023)). Its high-spatial-resolution global measurements have sufficient VSWIR bands to consider data fusion with Landsat-8/-9 and Sentinel-2. VIIRS also shares heritage from pioneering measurements such as the Advanced Very High-Resolution Radiometer (AVHRR) on NOAA’s polar-orbiting Environmental Satellites (POES) (Table 2).

**Figure 13:**
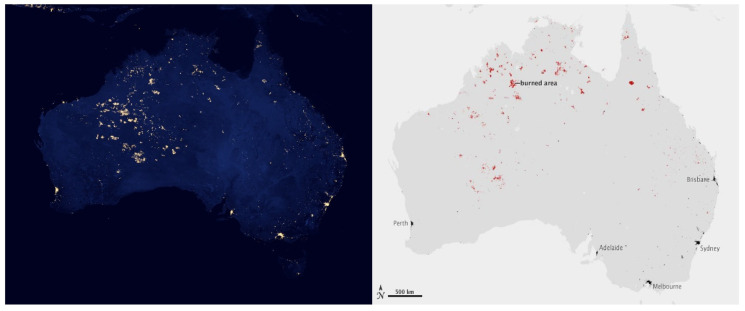
The day/night band of the VIIRS instrument on the Suomi NPP satellite provides information on sites where significant electrical energy is being used or where fires are active. The left panel shows a composite VIIRS day/night image of Australia, acquired over nine dates in April and 13 days in October 2012. Comparing locations using the panel on the right allows you to identify lights along the coastline from Australia’s major cities and from lights in outback regions where no large cities exist. Those lights are from wildfires that occurred during the two periods. While the cities have relatively consistent lighting, the wildfires are temporary and short-lived, but compositing the images you can see the total areas burned during these two periods. Images from NASA Earth Observatory (Robert Simmon) Image of the day (20 December 2012); images provided by Chris Elvidge (NOAA National Geophysical Data Center).

The visible band is designed for sensitivity to low light intensities at night [184]. The day/night band and Operational Line scan System (OLS) has heritage from the Defense Dept. Meteorological Satellite Program (DSMP; www.ospo.noaa.gov/Operations/DMSP/ (accessed on 22 January 2024)) but with better spatial resolution, dynamic range, and onboard calibration [185]. The https://ngdc.noaa.gov/eog/sensors/ols.htmlday/night data have attracted a wide range of applications, including relative economic status of different regions [120,186], population size estimates [184,187], wildfires [188], flaring from gas wells [189,190], and ship tracking [191,192]. VIIRS can detect clouds and high albedo terrain features in zero moonlight [185], essential for monitoring the polar winter. Nightlight data are designed to detect combustion, but also to distinguish thermal sources from persistent electric lighting using band M10 at 1.61 µm [188]. Figure 13 shows night lights in Australia measured by the day/night band on VIIRS. The data are a composite of 9 days in April and 13 days in October 2012. By comparison with the panel on the right it is easy to spot the major cities along the coastline, but there are no large cities in the northwestern area of many night lights. The two periods included many wildfires but by compositing the data into one image it dramatically illustrates how many fires occur there in these spring/fall transition periods. 

VIIRS data serve a wide range of applications in addition to weather and climate research, contributing to better understanding of large-scale land cover changes. These include regional droughts, wildfires, floods, and applications like crop prediction, food security, urban expansion, forest die off, increasing or decreasing agricultural land [193,194,195,196]. VIIRS provides data for agricultural management, fire monitoring [197] gas flaring [198], and maritime forecasting products for navigating sea ice and support fishing [191]. Waigl et al. [199] showed better detection of high and low intensity fires with the 375 m thermal band (I-5) on VIIRS, compared to MODIS. A wide range of land-use scientists, urban geographers, ecologists, carbon modelers, demographers, economists, and social scientists use these data successfully [184].

#### 7.1.3. VIIRS on NOAA 20 and 21

Following its success on the Suomi NPP, VIIRS became the imaging component of the first two new polar-orbiting operational weather satellites, NOAA 20 (2017/2027), which was called the Joint Polar Satellite System (JPSS-1) prior to launch and on the NOAA 21 (2022/2029) (JPSS-2) satellites for weather and climate (Table 10). NOAA 20 and 21 have ascending crossing times at 13:30 and descending crossing times at 01:30 [200,201]. In addition to VIIRS, NOAA 20 and 21 continue to carry the ATMS (Advanced Technology Microwave Sounder), CERES (Clouds and Earth’s Radiant Energy System), CrIS (Cross-Track Infrared Sounder), OMPS (Ozone Mapping Profiler Suite) and the RBI (Radiation Budget Instrument). The VIIRS is also planned to fly on the NOAA 22 (2028/2039) and NOAA 23 (2033/2041), along with a similar suite of instruments (ATMS, CrIS, and OMPS).

### 7.2. The Operational E.U. Polar Orbiting Copernicus Sentinel Satellites

Based on ESA’s successful satellite program in the last decades of the 20th century, the European Union (EU) initiated plans twenty-five years ago for an ambitious operational Earth monitoring program, which would be dependable and upgradable over time, to ensure a suite of satellites that “take the pulse of our planet and transform the way we see the world” (https://dataspace.copernicus.eu/events/2023-6-8-25th-anniversary-copernicus/ (accessed on 19 December 2023)). The previously described ESA/Earth Explorer program remains the primary trajectory to explore new ideas and concepts, with discovery and risks expected, and is complementary to the ambitious (and much larger) operational EO “Copernicus” program (Table 11). However, research components are included in most of these operational Copernicus-sponsored missions. Copernicus is managed by the European Commission, with partner agencies, including the European Space Agency (ESA), the European Organization for the Exploration of Meteorological Satellite program (EUMETSAT), member states, and other agencies. The initial operational Copernicus program consists of six EO instruments/satellite series (Sentinels), each flown in pairs as 2-satellite constellations to increase temporal collections. Replacements are intended every 5–7 years (or as needed) with upgraded capabilities in tune with developing technology and knowledge. Additional Sentinel series are expected in the future. Copernicus supports a data policy for full and open data access.

#### 7.2.1. The Sentinel-1 Satellites

The first Sentinel satellite in the Copernicus series is Sentinel-1, a radar platform launched in 2014 (Torres et al., 2012) to map global landmasses, obtaining three-dimensional surface measurements once every 12 days (Table 11), beginning with Sentinel-1A in 2014 and Sentinel-1B in 2016 (https://sentinel.esa.int/web/sentinel/missions/sentinel-1/ (accessed on 20 December 2023)). The Sentinel-1s fly in sun-synchronous LEOs at ~693 km altitude and have 40 m GSD observed across a 400 km swath. Sentinel-1A (S-1A) hosts a C-band (5.405 GHz) Dual Polarized Synthetic Aperture Radar (SAR) instrument with four different imaging modes (Table 9). S-1B was terminated in 2022 due to instrument failure and will be replaced with S-1C in 2024, as will S-1A which will be replaced by S-1D. The next pair (S-1E, S-1F) are expected to be launched in the mid-2030s.

Sentinel-1’s mission is to improve global estimates of carbon stocks from measurements of forest height and biomass [202,203], ground and ice surface topography [204,205] and monitor environmental disturbances, e.g., [206,207]. These goals are pursued by monitoring vegetation height and density, changes in topographic height (e.g., from volcanic activity, earthquakes, and windstorms), or changes in vegetation height (from growth, harvesting, wildfires, floods, and weather-related disasters) that can be derived from repeated acquisitions.

**Figure 14:**
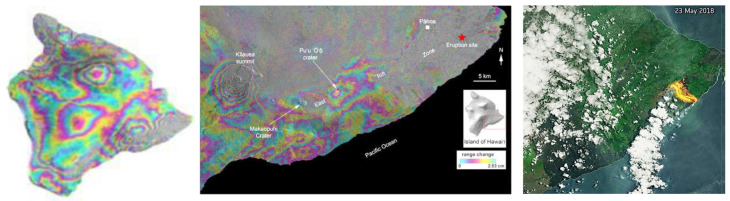
Sentinel-1 images of the months-long eruption of Hawaii’s Kilauea volcano on the Big Island in May 2018. This sequence of the eruption was extraordinary and an accompanying 6.9 magnitude earthquake on 4 May 2018 but it was also one of the most destructive in its history as 1.4 km^3^ of lava destroyed much of the settlement of Leilani Estates. The left image is a Sentinel-1B interferometry image for the 12 days from 23 April to 5 May 2018.The the eastern area shows tightly set fringes that show where the greatest movement occurred. On 30 April the floor of the small Pu‘u ‘Ō‘ō collapsed as magma moved eastward along the East Rift Zone and eventually erupted in the lower East Rift zone, the area of dense fringe pattern is the rift zone from which the fisher erupted. The right image shows the lava eruption on 23 May 2018. Hawaii experienced a prolonged period of extreme rainfall that increased pore pressure up to 3 km depth that triggered the eruption, combined with increased vertical displacement in the rift zone around the area of the fissure (https://www.esa.int/Applications/Observing_the_Earth/Copernicus/Sentinel-1/Can_rain_trigger_a_volcanic_eruption/ (accessed on 15 May 2024)). Data from the ESA Sentinel Online (https://sentinel.esa.int/web/success-stories/-/sentinel-1-interferogram-of-kilauea/ (accessed on 15 May 2024)) and NASA Applied Sciences (http://appliedsciences.nasa.gov/our-impact/news/sentinel-1-interferogram-kilauea-eruption-quake/ (accessed on 15 May 2014)).

The Sentinel-1 constellation provides daily routine coverage of European coasts and sea ice zones, including shipping routes, and bi-weekly radar interferometry coverage to monitor the Earth’s land areas. Each satellite’s radar instrument operates in four modes to acquire data at different spatial resolutions and areal coverage. In the interferometric wide-swath mode, any S-1 satellite pair can map land masses globally every 6 days (individually every 12 days). Data are available from the Copernicus Data Space Ecosystem (https://dataspace.copernicus.eu/ (accessed on 4 December 2023)). User guides are available at Sentinel Online (https://sentinels.copernicus.eu/web/sentinel/user-guides/sentinel-1-sar/definitions/ (accessed on 4 December 2023)). Figure 14 shows a Sentinel-1 SAR interferogram image of eruption of Kilauea volcano on the island of Hawaii in May 2018. 

#### 7.2.2. The Sentinel-2 Multispectral Imagers (MSI)

The next mission series, Sentinel-2, was also initiated in 2014 (https://www.esa.int/Applications/Observing_the_Earth/Copernicus/Sentinel-2, accessed on 13 December 2023). The mission of the Sentinel-2 program is to monitor global vegetation patterns, tracking changes in vegetation distributions (Table 11). and collecting evidence of plant physiological stress responses. Its time series data provide increased accuracy of classification mapping for land and coastal areas, e.g., [208,209,210,211].

Each S-2 satellite carries the Multispectral Instrument (MSI) (Drusch et al., 2012), the first space-based imager designed to measure passive reflected sunlight across the full visible to shortwave-infrared (VSWIR) wavelengths, in thirteen spectral bands (resolution is band dependent, 10–20 nm). The first Sentinel-2 satellite, Sentinel-2A (S-2A), was launched in 2014 followed by S-2B in 2017 (Table 11), and together they provide repeat global coverage from polar LEO every five days. The Sentinel-2s multispectral instruments are comparable to the L-8/9 duo and they have a similar mission, but the S-2s have more spectral bands (13) at higher (10–20 m) spatial resolutions [212]. Also, the LEO at 786 km for the Sentinel-2 satellites is at a higher altitude than Landsat, providing a wider swath of 290 km and a 10-day single satellite revisit, or a five day two-satellite revisit.

The mission of the Sentinel-2 program is to monitor global vegetation and land-use patterns and collect evidence of plant physiological stress responses. Its time series and additional spectral band data provide increased accuracy of classification mapping, e.g., [213,214,215,216].

Sentinel 2A and 2B share similar mid-morning equatorial crossing times (10:00–10:30 local time) with Landsats-8 and 9. With these four satellites in orbit, there is now an opportunity to obtain repeat multitemporal global coverage every 2–3 days, which became possible after L-9 was launched in 2022. This has meant that Europe’s agricultural crop monitoring plans, first envisioned in NASA’s 1975 LACIE project, can now be realized (even in semi-cloudy environments), as well as making it possible to follow phenological development of larger-scale vegetation patterns across the globe, and regional responses to environmental stresses such as drought. The increased spectral bands and higher sptial resolution improves classification accuracy and numbers of classes identified, which again improves when multitemporal data are used in the classifier programs [214,217,218,219].

The Sentinel-2 satellites, with higher spatial resolution and more spectral bands, were poised for quick adoption at launch, given the success of Landsat-8 launched the previous year. The Sentinel-2s have provided unprecedented 5-day repeat coverage at relatively high (10–20 m) spatial resolution with 13 spectral bands and flown in a familiar 10:30 orbit.

The Copernicus program is already preparing for the launches of two more Sentinel-2s, S-2C in early 2024 followed by S-2D in 2025. This will likely mean the retirement of S-2A and provide new pairings for S-2B and S-2C. Later, S-2C and S-2D will pair to ensure continuity with the current Sentinel-2s and with current and future Landsat missions. Together, this strategy will provide an even richer global observation dataset with improved spectral, spatial, and temporal coverage to enhance and extend the satellite record for these advanced products, available from 2013 forward through the 2030 decade.

Demand for Sentinel-2 data products greatly exceed expectations and a search on Google Scholar at the end of 2023 yields approximately 25,900 results. The range of environmental and remote sensing topics spans most of the sweep of Landsat (however, the S-2’s do not have a thermal imager onboard). Publications using Sentinel-2 data parallel the pattern of increased use of Landsat data [220], demonstrating the worldwide demand for data with these attributes for societal applications. A Special Issue on “Science and Applications with Sentinel-2” in Remote Sensing of Environment (June 2021), curated 28 open access papers that demonstrate the range of topics in early papers. The journal Remote Sensing (ISSN 2072-4292) also published 20 papers in a “Sentinel-2: Science and Applications” in 2023 (https://www.sciencedirect.com/journal/remote-sensing-of-environment/special-issue/1045CSHL53V/ (accessed on 3 May 2024)).

Combining the data acquired by the Sentinel-2s and Landsat-8, -9 has increasingly been used to understand dynamic processes, e.g., changing lake level heights [221]. The demand for merged Landsat-8 and -9 data with Sentinel-2A and -2B satellites provides more frequent access, given the combined 2–3 day repeat cycle, of significant importance to the user community [222]. This is addressed below for Harmonized Landsat-8, -9 and Sentinel-2A, B products.

#### 7.2.3. Harmonized Landsat-8 and L-9 and Sentinel-2 (A, B) Data

**Figure 15:**
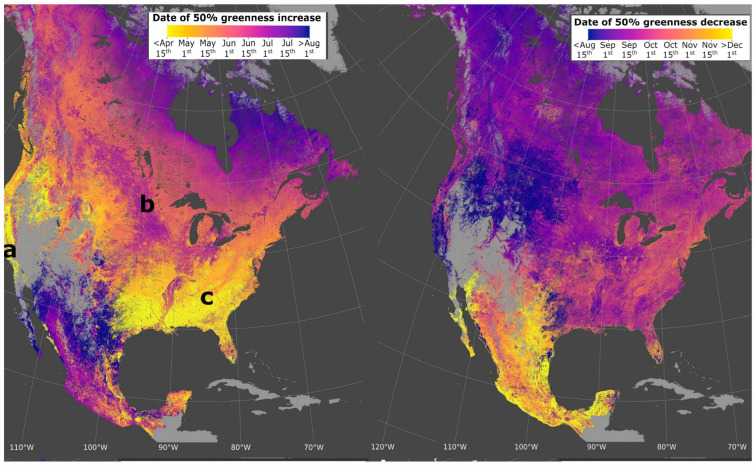
Continental-scale land surface phenology from harmonized Landsat-8 and Sentinel-2 imagery. These high temporal resolution continental-scale maps of North America show (Left) the day of year (DOY) when 50% spring-time green-up occurred, and (Right) the DOY when 50% autumn Green-down occurred. The DOY varies with the location, site condition, and species present, but at this scale, the primary driver seems to be continental climatology. The letters a, b, c are from Bolton et al. [227] and represent three different ecosystems and climates, with (a) the Salinas Valley, California (Coastal shrub and mixed evergreen and deciduous forest in mountains and agriculture in the valley), (b) agricultural fields in North Dakota, and (c) the Great Smoky Mountains with deciduous forest at the border of Tennessee and North Carolina, USA. Figure modified from Bolton et al. [227] and reproduced with permission.

The Landsat program’s interest in improving temporal coverage led to a special NASA-ESA project to combine data from Landsat-8/9 with Sentinel-2A,2B to develop a merged version of the two datasets (Figure 15), yielding “harmonized” Landsat and Sentinel satellite data products [223]. Table 12 compares the orbits and bands of the two datasets. The S-2s have 11 VSWIR bands and 2 SWIR bands with spatial resolutions varying between 10 and 60 m, but no TIR bands. Differences in data characteristics between L-8/9 and the Sentinel-2s were natural consequences of their different orbit orientations, 30 min temporal offsets, spatial resolutions, band widths and wavelength placements, and differences in reflectance products, all of which needed to be resolved to enable co-registration of the datasets [224]. Claverie et al. [223] report three products from the combined data: Multispectral Instrument (MSI) reflectance products at full 10–20 m spatial resolution; a 30 m MSI Nadir Bidirectional Reflectance Distribution Function (BRDF)-adjusted reflectance (NBAR) product, and a 30 m Landsat OLI NBAR product, e.g., [225,226,227,228].

The success of these pairs of Landsat and Sentinel 2 has contributed to plans developed by the Landsat Next project to adopt the constellation concept, as well as to add more and narrower spectral bands to its next imaging spectroradiometer.

The map in Figure 15 was created from the EVI spectral index measurements, using an inclusive procedure for the 24 months, 2016 to 2018. This allowed Bolton et al. [227] to identify the critical points in the annual vegetation growth curves. The DOY varies with location, given varying site conditions and species. The points labeled “a, b, and c” on the original figure indicate examples sites with different climates and species. Continental-scale high spatial (30 m) resolution land surface phenology produced from harmonized Landsat-8 and Sentinel-2 imagery.

### 7.3. Sentinel-3 Hyperspectral Imager Constellation

The Sentinel-3 mission is a two-satellite constellation to monitor surface topography, sea/land surface temperatures, and ocean and land color (Table 11) (https://sentinel.esa.int/web/sentinel/missions/sentinel-3/satellite-description/ (accessed on 13 January 2024)). Sentinel-3 was established to create a European capacity for EO that provides policy makers and public authorities with timely information on managing the environment, climate change, and civil security. Sentinel-3 provides global estimates of surface albedo for the Copernicus Climate Change Service [229]. Global Monitoring for Environment and Security (GMES) is the overall Sentinel-3 mission [230]. The S-3A and S-3B were launched in 2016 and 2018, respectively, and S-3C and S-3D are expected to be launched in 2024 and 2025; the new ESA Earth Explorer #8, FLEX, will fly in tandem with S-3D (Refer to Section 14.3).

#### 7.3.1. The Ocean and Land Color Instrument (OLCI) Imager

OLCI has heritage from MERIS on EnviSat which flew from 2002 to 2012 (https://earth.esa.int/eogateway/instruments/meris/ (accessed on 13 May 2024)) and has precise positioning to within 0.1 pixel. OLCI is a wide-swath (1270 km) moderate-spatial resolution (300 m) multiband imaging spectrometer, with five cameras oriented across an angle of 68.6° perpendicular to the platform direction, optimized to avoid sun-glint. It measures 21 defined spectral VNIR bands (between 400 and 1020 nm, pre-selected from VNIR spectrometer data, which are fixed for operational purposes, but programmable for special purposes). Bandwidths for ten bands are 10 nm, while six bands are narrower (2.5–7.5 nm), and five bands are wider (15–40 nm). These mostly narrow bands were selected to capture information about plant pigments, evapotranspiration, water vapor, and aerosols, etc. [231,232,233,234].

**Figure 16:**
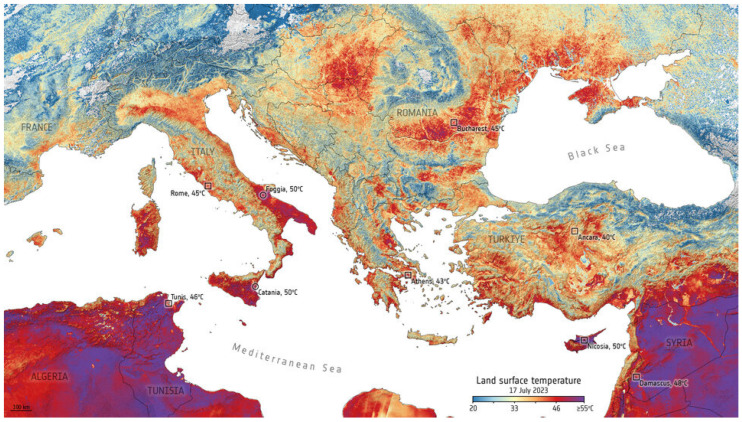
Sentinel-3 SLSTR image of the land surface temperatures at the 11:00 (CEST) morning of 17 July 2023 European heatwave. As this is a morning temperature, maximum temperatures were generally much higher by late afternoon, as shown for various cities in the figure, with most of north Africa will above 50C and European cities up to 50C. High temperatures continued into August as the hottest summer on record/(https://www.esa.int/ESA_Multimedia/Images/2023/07/Heatwave_across_Europe#:~:text=This%20image%20uses%20data%20from,and%2047%C2%B0C%2C%20respectively/ (accessed on 13 May 2024)). The impact of such high temperatures for extended periods an excess number of deaths. The ESA reported a study from the 2022 heatwave that caused up to 60,000 excess deaths (https://www.esa.int/Applications/Observing_the_Earth/Copernicus/Sentinel-3/Europe_braces_for_sweltering_July/ (accessed on 16 May 2023)).

Figure 16 Sentinel-3 SLSTR data shows a large heatwave, called the Cerberius Heatwave that brought the hottest temperatures ever recorded in Europe, especially severe over southern Europe and north Africa in summer 2023. This heatwave started 10 July 2023 from the Cerberus anticyclone (named after the hound of Hades from Greek mythology) with surface temperatures exceeding 50C in many places. They experienced a second heatwave in late August 2023. Excessive heat extended shifted around the region for many days, due to climate warming amplified by El Niño. The heat stress across Europe began early in summer and continued into September.

#### 7.3.2. The Sea and Land Surface Temperature Radiometer (SLSTR)

The SLSTR is S-3’s second optical instrument (Table 11), with heritage from the Along-Track Scanning Radiometers (ATSR) previously flown on the European Remote-sensing Satellites 1 and 2, ERS-1 (1991–2000) and ERS-2 (1995–2011) and on EnviSat (https://earth.esa.int/eogateway/instruments/aatsr/ (accessed on 19 February 2024)). SLSTR’s main objective is to provide global sea-surface temperature measurements with zero bias and uncertainties of ±0.3 K, as well as land surface temperature readings and fire monitoring [221,229,235]. The SLSTR has an along-track dual-view scanning technique, enabling acquisitions at nadir and along-track in the backward direction, including measurements of two blackbody calibration targets and a Visible Calibration Unit for high accuracy.

The SLSTR has a swath of 2420 km and provides 500 m GSD for its 7 VSWIR spectral bands: three in VNIR (~20 nm wide), four in MIR and SWIR (50–60 nm wide); the GSD for its two TIR bands (3.75 μm and 12 μm, each 400–900 nm wide) is twice as large at 1000 m. There are also two fire channels (sensitive to high thermal IR) with expanded dynamic ranges to prevent saturation: F1 at 3.7 μm and F2 at 10.8 μm, also with 1000 m GSD [236]. In the single-view mode, SLSTR has a spatial resolution of 1 km, with less than half a day revisit time and a swath of 1400 km. In the dual-view mode, it reaches 500 m resolution with daily revisits but only covers a swath of 744 km.

There are also active systems onboard: the SRAL (Synthetic Aperture Radar Altimeter) to measure sea-surface topography and height in open ocean and coastal areas, as well as topography on land (with 1/20 Hz Ku/C band waveforms). The SRAL has heritage from Envisat and Jason-2/Poseidon-3 heritage (https://sentinels.copernicus.eu/web/sentinel/technical-guides/sentinel-3-altimetry/ (accessed on 26 February 2024)). The sea-surface topography measurements were intended to meet the quality standard of the altimetry system previously achieved with Envisat. The second instrument is a MicroWave Radiometer (MWR) that supports the SRAL altimeter mission by providing a correction for wet atmospheres, derived from EnviSat MWR heritage. The MWR measures brightness temperature at 23.8 and 36.5 GHz, covering 200 MHz bandwidths in each channel, which are sensitive to atmospheric water vapor, and cloud liquid water, respectively.

#### 7.3.3. Three Instruments That Provide High Precision Onboard Orbit Tracking on Sentinel-3

The Doppler Orbitography and Radiopositioning Integrated by Satellite (DORIS) is a satellite tracking system designed by the French Space Agency, CNES. The S-3 has a new-generation, multichannel and digital receiver that can track up to seven beacons simultaneously. The DORIS monitors the motion of the satellite with respect to the known position of the beacons, which induces a doppler shift that is proportional to the radial speed of the satellite. Knowledge of the location of each beacon allows precise position of the satellite, with real-time accuracy of 5–10 cm (https://sentinels.copernicus.eu/web/sentinel/technical-guides/sentinel-3-altimetry/instrument/ (accessed on 26 February 2024)). The S-3 GNSS receiver captures signals from the GPS constellation, up to eight satellites and two bands in parallel, capable of real-time position accuracy of 1–2 m for position and 2–3 mm/s for velocity. Lastly, the **L**aser **R**etro-**R**eflector (**LRR**) is a passive target for laser tracking measurements from dedicated ground stations. It hosts a hemispherical array of seven corner cubes that reflect incoming laser pulses from the ground stations, with an accuracy of a few millimeters.

### 7.4. Sentinel-4, -5P, and -5 Satellites

These three satellite constellations will work together in different orbits (polar and GEO) to monitor atmospheric chemistry. The first in a polar orbit is the Sentinel-5 Precursor (S-5P) that was introduced in Section 5.10 and has been flying since 2017. It carries the first of the TROPOMI instruments. It is expected to continue to 2027 by which time the Sentinel-5 TROMPOMI instrument will be flown on the polar-orbiting METOP Second-Generation (SG) satellite 1A (Section 14.3) and TROPOMI will also be flown on Sentinel-4, which will be launched with the third-generation geostationary satellite (MTG) I-1A (Section 11.5).

### 7.5. Sentinel-6 Michael Freilich

Sentinel-6 was developed in the Copernicus program by the European Space Administration (ESA), with contributions from EUMETSAT, NASA and NOAA, and NCES (France) (Table 11). Its mission is to provide high-accuracy sea-surface topography that supports ocean forecasting, sea-level rise, and environmental and climate monitoring [237,238]. Sentinel-6 has heritage from the TOPEX/Poseidon and Jason (1, 2, 3) missions, and S-6 is also called the Jason Continuity of Service (Jason-CS) mission on the Sentinel-6 spacecraft because it was originally planned as a follow-on to several generations of satellites monitoring sea-surface topography [238,239].

Jason-CS/Sentinel-6 has two identical satellites, with the first launched 21 November 2020 (formerly designated as S-6A) and renamed in February 2020 to honor Dr. Michael Freilich (as the Sentinel-6 Michael Freilich, oceanographer and former Director of NASA Earth Science Division from 2006 to 2019 who championed oceanographic research, https://www.eumetsat.int/sentinel-6/ (accessed on 26 January 2024)). The second Sentinel-6B/Jason-CS is scheduled for launch in 2026. The instrument system for this series is focused on its high precision Synthetic Aperture Radar Altimeter satellite system (with six instruments on the two platforms). The Sentinel-6 constellation will work with the Sentinel-3 constellation, combining to monitor sea-level changes over the next two decades.

Poseidon-4 is the primary payload on the Sentinel-6 mission, with heritage from Sentinel-3 (Table 11). It consists of a nadir-pointing dual-frequency SAR altimeter (a Ku-band, 13.575 GHz) and a C-band (5.41 GHz), and the Multifrequency Advanced Microwave Radiometer for Climate (AMR-C). Poseidon-4, with heritage from Sentinel-3 simultaneously acquires sea-surface height from the radar measurement and Significant Wave Height and wind speed from the normalized radar cross-section acquired in the conventional low-resolution mode, but with data processing approaches high precision altimetry measurements. These simultaneous measurements produce twice the number of measurements as the Sentinel-3 SRAL instrument. The Advanced Microwave Radiometer for Climate (AMR-C) provides data to correct for signal delays due to atmospheric attenuation by water vapor that introduces large spatial and temporal variability. The AMR-C has heritage from SRAL in earlier Jason-2 and Jason-3 and Sentinel-3 missions. Lastly, the AMR-C includes an experimental system that supports the high-resolution SAR mode that extends the microwave retrievals closer to the shoreline under cloud-free conditions.

With a new instrument onboard, the precipitation sensitive Global Navigation Satellite System Radio Occultation (GNSS-RO), system, Jason-CS/Sentinel-6 also contributes to weather prediction. Observing GNSS satellites as they disappear over the horizon will provide detailed information about the layers in the atmosphere, expected to enhance predictions for weather and forecasting capabilities.

## 8. A New Generation of Imaging Spectrometers: Europe’s Pioneers

Successes with hyperspectral imagers (HSI) and imaging spectrometers (IS) were previously demonstrated in multiple airborne and science missions over a 35-year period of technology development and science evaluation, but previous spaceborne imaging spectrometers in space were not dedicated to land surface monitoring. Today, ISs in space are now a reality with two European satellite missions carrying advanced “full-spectrum” imaging spectroscopy technologies (which are 400–2500 nm, with spectral band resolution of 12 nm or better, moderate spatial resolution <100 m, and improved SNR per band (VNIR; 200–600; SWIR: 100–200). These satellites are flying in morning LEO orbits. Italy’s PRecursore IperSpettraie della Missione Applicativa (PRISMA) was launched in March 2019 [240,241] and the German Space Agency (DLR)’s Environmental Mapping and Analysis Program (EnMAP) was launched in late 2022 (Table 13). Both imaging spectrometers represent “next steps”, drawing on technologies and sampling capabilities that were not available for the first polar-orbiting imaging spectrometer in space—NASA’s EO-1/Hyperion technology demonstration instrument, over two decades ago. Although these two missions have upgraded technologies, neither has the capacity to obtain global collections. The two demonstration satellites have heritage from CHRIS on the PROBA-1 satellite (Section 3.4) and Hyperion on the New Millennium EO-1 satellite (Section 4.2.2), both launched into sun-synchronous LEO orbits with descending mid-morning (10:30 PRISMA, 11:00 EnMAP) local equatorial overpass times. Both acquire spectra across relatively narrow 30 km path widths at 30 m GSD, recording full-range VSWIR spectral collections. While maintaining a similar ability to distinguish geometric characteristics as previous imaging satellites, their additional hyperspectral and spatial sampling capabilities enable better characterization of the chemical-physical composition of the surface materials based on their unique spectral signatures [242]. The PRISMA and EnMAP programs provide steppingstones along the pathway to prepare for the future global operational IS monitoring era, starting with NASA’s Surface Biology and Geology mission, SBG-VSWIR (Section 16.1) and ESA/EU’s CHIME in the early 2030s-time frame (Section 17.2).

### 8.1. PRecursore IperSpettraie Della Missione Applicativa (PRISMA)

The Italian Space Agency (Agenzia Spaziale Italiana, ASI) launched its 5-year hyperspectral mission (https://www.asi.it/en/earth-science/prisma/ (accessed on 12 November 2023)) in 2019 into orbit at an altitude of 614 km with an inclination angle of 98.19°, which provides a nadir repeat measurement frequency of 29 days, and a 10:30 Equatorial crossing time [240,241]. The mission also offers optional off-nadir viewing at ±1000 km. PRISMA data are acquired for areas/sites located between 180° W–180° E and 70° S–70° N, with capacity on each overpass to collect images along the flight paths ≤1000 km (https://www.eoportal.org/satellite-missions/prisma-hyperspectral/ (accessed on 3 December 2023)). Archived and new PRISMA data are available after user registration (https://prismauserregistration.asi.it/ (accessed on 12 November 2023)); documentation and product specifications are available in the same portal for data search and download (https://prisma.asi.it/ (accessed on 12 November 2023)). PRISMA data are available for access (http://www.prisma-i.it/index.php/en/ (accessed on 12 November 2023)).

The PRISMA (Table 13) carries two modules that utilize push-broom scanning: (i) the Hyperspectral Camera (HYC), a prism spectrometer that covers two spectral regions, the VNIR and the NIR/SWIR, with a total of 237 bands; and (ii) a Panchromatic Camera (PAN) with a high-fidelity panchromatic band collecting data at 5 m spatial resolution. Both VNIR and SWIR have an average bandwidth of 10 nm. The term “high-fidelity” applied to spectral bands indicates cross-track spectral uniformity (that is, the full-width at half maximum response, FWHM, is the same in all pixels) and has IFOV uniformity at all wavelengths [43,243]. The PAN and HYC data are co-registered. 

PRISMA’s primary mission is to monitor natural resources for land and coastal zones at relatively high spatial resolution, to develop algorithms for environmental applications, and to evaluate the technologies supporting its hyperspectral payload [244,245]. Because the expected EOL for PRISMA is March 2024, the ASI initiated a feasibility study in 2022 for a second-generation PRISMA. The second-generation PRISMA satellite, PRISMA2GEN, is now planned for launch in 2028 with a 7-year mission.

**Figure 17:**
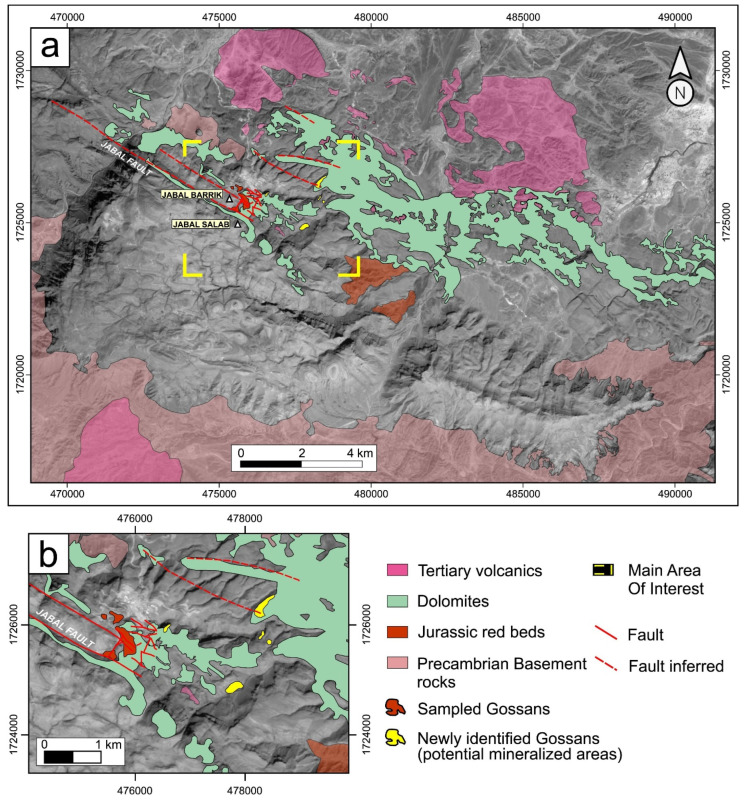
The spectral resolution of PRISMA data allows it to access major absorption features in the solar region of the spectrum, thus providing data to map many geologic minerals with PRISMA data. Panel (**a**) show an area of the Sab’atain basin of the Jabali region of Western Yemen, which has hydrothermal dolomitization and supergene alternation associated with zinc (Zn), lead (Pb) and silver (Ag) deposits. Panel (**b**) shows an enlargement (within yellow brackets in Panel (**a**)) to better see the pattern of the mineralogy. The interpreted mineralogy is shown over a panchromatic image, emphasizing the topography. The main rock types include limestone, dolomite, Precambrian basement granites, and Tertiary volcanic products, all mapped from their spectral differences detected in spectral band depths at wavelengths where absorptions occur. The authors mapped gossans (rust-colored oxides and hydroxide minerals of iron and manganese that cap ore deposits) by detecting the Fe^3+^ absorption band at the 900 nm region and the Zn-Pb mineralized locations were identified from the gossan occurrences in the dolomites (figure from Chirico et al. [246] and reproduced with permission).

An example of PRISMA data is shown in Figure 17 of a hydrothermal alteration zone comprised of carbonate (dolomite) deposits associated with Late Jurassic rifting, in the Jabali district in western Yemen [246]. This area has outcrops of diverse mineralogy, including zinc, lead, and silver. The upper image shows a panchromatic image for the background which is overlayed with a mineral and carbonate map developed from spectroscopy and geochemical analyses, to bring out mineralogical differences based on electronic and vibrational absorptions.

### 8.2. Environmental Mapping and Analysis Program (EnMAP)

Launched in 2022, EnMAP is a high-quality hyperspectral mission of the German Aerospace Center (DLR) to characterize the Earth’s environment (https://enmap.org/ (accessed on 20 November 2023)) [40]. The EnMAP mission’s main objective is to provide high-quality, regional-scale hyperspectral data to improve understanding of coupled environmental processes (e.g., crop growth and drought), thereby assisting in the sustainable management of Earth’s resources. The mission will evaluate spaceborne hyperspectral data for use in future monitoring of societal applications, e.g., agriculture, water conditions and management, land-use changes and environmental health, geology, and urban management.

The EnMAP is a push-broom imager, with an equatorial morning overpass time at 11:00 (±18 min) (Table 13). It flies a full hour later than the Landsat satellites (10:00 ± 15 min), and slightly later than overpass times at 10:45 ± 15 min for ESA’s Sentinel-2 imager, and the two future ISs under development: SBG (NASA) and the ESA’s Copernicus Hyperspectral Imaging Mission for the Environment (CHIME) (Section 16.2). EnMAP has an orbit altitude of 653 km and a 27-day revisit interval with nadir viewing. It collects off-nadir (≤30°) data at four-day intervals with its cross-track pointing capability to capture special events, a critical function previously demonstrated by NASA’s EO-1 demonstration satellite. It collects 1000 km of imagery per orbit and 5000 km per day (e.g., 1000 km for five orbits). The specifications for EnMAP’s spectral bands (e.g., 6.5 nm average spectral sampling for VNIR bands exceed or match those of its contemporaries (https://space.oscar.wmo.int/satellites/view/enmap/ (accessed on 16 November 2023))).

## 9. The International Space Station (ISS) Hosts Experimental Instruments from a Non-Polar Orbit

The International Space Station (ISS) is the largest modular space station in low Earth orbit. It began as an international coalition in 1998, whose primary space agency supporters of programs or operations are NASA (United States), Roscosmos (Russia), JAXA (Japan), ESA (Europe), and CSA (Canada). Since Nov. 2000, astronauts have conducted a mind-boggling suite of more than 3000 studies in 20+ years of operations on board (https://www.nasa.gov/international-space-station/space-station-research-and-technology/space-station-science-101/ (accessed on 5 January 2024)). In addition to serving as an observatory and factory, the ISS serves as a scientific research laboratory in many fields, including astrobiology, astronomy, meteorology, physics, Earth remote sensing, and medicine. The largest single module of the ISS is JAXA’s Japanese Experiment Module (JEM), nicknamed “Kibo” (meaning Hope), with six major components, including the Exposed Facility where payloads are mounted for Earth Science studies. Several are described below (https://iss.jaxa.jp/en/kibo/ (accessed on 7 January 2024)).

The most recent modular components were added in July 2021—Europe’s relocatable Robotic Arm and Russia’s primary “Nauka” research module; Russia subsequently docked its “Prichal” nodal module later in 2021 (https://blogs.nasa.gov/spacestation/tag/prichal/ (accessed on 7 January 2024)). In January 2022, the United States extended authorization and funding for operations until 2030 and announced a planned date of January 2031 to de-orbit the ISS, with any remnants directed into a remote area of the South Pacific Ocean. In September 2022, the Head of Roscosmos, Yuri Borisov, stated that Russia was “highly likely” to continue to participate with the ISS until 2028. NASA has initiated a Commercial LEO Development program, to further the development of private on-orbit capabilities after the ISS era. This will likely replace the current ISS opportunities for testing experimental Earth-observing instruments.

The ECOSTRESS instrument provides heritage for the NASEM recommended “Designated Observable” NASA SBG-Thermal Infrared imager, the EMIT is also heritage for the SBG VSWIR imager, and the GEDI is in consideration for a future vegetation structure mission. The DESIS instrument provided opportunities to test and validate algorithms for EnMAP’s imaging spectrometer currently in orbit (Section 8.2).

The ISS flies at average minimum and maximum altitudes of 370 km and 460 km, respectively. Since atmospheric drag at that relatively low height above Earth (compared with LEO polar-orbiters) reduces the altitude by approximately 2 km a month; its average 400 km altitude is attained by means of re-boost maneuvers using the engines of the Zvezda Service Module, or visiting spacecraft. Maintaining the altitude of the ISS requires ~7.5 tons of chemical fuel each year (costing ~USD 210 million). The ISS circles the Earth in approximately 93 min, completing 15.5 orbits per day, traveling at an average speed of 28,000 km h^−1^. The station is currently maintained in a nearly circular orbit at an inclination angle of 51.5° relative to Earth’s equator, with an eccentricity value of 0.007. This orbit limits Earth observation coverage to between ±52° north and south latitudes. However, the elliptical orbit does provide opportunities to view at different times of day, which can give important (although limited) information about diurnal changes, but it also produces temporal clusters and data gaps.

The relatively low altitudes for the ISS orbits make it vulnerable to a variety of space debris, in addition to natural micro-meteoroids. These include spent rocket stages, defunct satellites, explosion fragments (including materials from weapons tests), paint flakes, slag from solid rocket motors, and coolant released by nuclear-powered satellites (https://www.space.com/international-space-station-space-dodge-debris-how-often/ (accessed on 5 January 2024)). These smaller debris objects are a significant threat, whereas objects large enough to destroy the station can be tracked and avoided, thus they are not as dangerous. Larger space debris is tracked remotely from the ground, and the station crew can be notified. If necessary, thrusters on the Russian Orbital Segment can alter the station’s orbital altitude, avoiding the debris. 

### 9.1. Experimental Missions from a Non-Plar Orbit for Advancing Earth Remote Sensing from the ISS

Remote sensing of the Earth from space has proven to provide invaluable information for several reasons: (i) it makes the collection of repeated, science-quality data possible; (ii) it is essential for viewing dangerous or inaccessible areas; (iii) it replaces costly and slowly acquired ground-based data collections; and (iv) it is non-invasive. Our particular interest in the ISS is because the ISS provides an essential platform to host experimental pathfinder instruments for Earth System Science in a space environment to verify their laboratory and/or aircraft measurement and stability capabilities, and to develop onboard data processing and calibration procedures. Most of these experimental instruments have been placed via Robotics on the external “Exposed Facilities”, primarily Kibo on the Japanese Experimental Module (JEM), which are hosted and serviced by astronauts for various time periods, usually between one to four years. Operational or longer-term Earth-viewing payloads are usually placed on the precision-pointing Multi-User System for Earth Sensing (MUSES) platform developed by Teledyne Brown Engineering, which can accommodate four payloads simultaneously. The first instrument hosted on the MUSES platform is the German Aerospace Center’s (DLR) Earth Sensing Imaging Spectrometer (DESIS), installed in June 2017. Examples of Earth-viewing remote sensing experiments that have previously flown on the ISS are the ISS-RapidScat (https://www.jpl.nasa.gov/missions/international-space-station-rapid-scatterometer-iss-rapidscat/ (accessed on 26 January 2024)) and the Cloud Aerosol Transport System (CATS; https://cats.gsfc.nasa.gov/media/docs/CATS_Data_Products_Catalog.pdf (accessed on 26 January 2024)).

The first spectrometer hosted on the ISS between 2009 and 2014 was the US Navy’s Hyperspectral Imaging for Coastal Ocean (HICO), a VNIR instrument (https://oceancolor.gsfc.nasa.gov/data/hico/(accessed on 8 January 2024)) measuring 128 spectral bands (~5.7 nm) in the UV through NIR (353–1080 nm), wavelengths and SNR (200:1) necessary for ocean penetration and for studying coastal processes (data below 400 nm or above 900 nm were not recommended for research). Smoothing filters were applied to the spectra (10 nm in VIS, 20 nm in NIR). HICO was lost due to a solar flare that irreparably damaged its computer in September 2014. HICO collected >10,000 scenes over five years, level 1b data is available at the NASA Ocean Color website (https://oceancolor.gsfc.nasa.gov/data/hico/ (accessed on 15 January 2024)) in netCDF4 format.

There are currently five Earth Science experimental imaging instruments hosted and operating on the ISS: DESIS, EMIT, HISUI, ECOSTRESS, and OCO-3. These are pathfinder instruments placed on the ISS for limited time periods to confirm their EO capabilities (usually 1–2 years) and to develop the future space-born LEO polar orbiter design systems and the methods for processing and interpreting the data (Table 11). All five instruments are functioning well and are likely to be operatable until the termination of the ISS. Two of these are scheduled to end their terms on the ISS during 2024: DESIS (after >6 y) and HISUI (after ~5 y). The terms for three of these instruments have been extended until 2026: ECOSTRESS (for 8 y), OCO-3 (for 7 y), and EMIT (for 4 y). A sixth instrument—GEDI—was moved in March 2023 (after 4 successful years) to onboard storage and awaits resumption of its mission in fall 2024 when it will resume data collection until it fails or the termination of ISS in 2031. 

### 9.2. DLR’s Earth Sensing Imaging Spectrometer (DESIS)

**DESIS** has been collecting science grade VNIR push-broom imaging spectrometer data from orbit for over six years (Table 14), since 2017 [247,248]. It is a VNIR-only imaging spectrometer measuring wavelengths from 400–1000 nm with 2.55 nm sampling in 235 (no-binning) bands, giving it unprecedented spectral resolution of 3.3 nm per band (https://www.tbe.com/what-we-do/markets/space/geospatial-solutions/desis/instrument/ (accessed on 14 May 2024)). The purpose of this high spectral resolution is to allow DESIS to make advances in characterizing vegetation health and stress, detected by changes in pigment composition and concentrations, especially in response to water quality and pollution stressors, as well as to support mapping the Earth’s mineral resources. DESIS supplements its 63-day nadir repeat frequency with its ±15° fore and aft capability from a Pointing Unit with 3° steps; it can select target measurements as frequently as every three days. Data can be requested, and the satellite can be tasked by registering with the DLRs portal at the EOWEB Geoportal (http://eoweb.dlr.de/egp/ (accessed on 14 May 2024)) and providing a short proposal for your planned use of the data. Data are delivered in 30 m × 30 m blocks, from fully automated processors that work on tiled data (1024 × 1024 pixels) covering ~900 km^2^. It runs on user’s requests and considers processing parameters defined by the users. Access to the 2.55 nm band pass data requires a request and permission. More information about data products is available at Alonso et al. [249]. Three product types can be ordered, which are Level 1B (systematic and radiometric corrected), Level 1C (geometrically corrected) and Level 2A (atmospherically corrected). The spatial resolution is approximately 30 m at the pixel scale. A study by Aneece et al. [250] compared agricultural image classification data from the Central Valley, California in 2020, between DESIS 30 m (hyperspectral) and PlanetScope’s Dove-R (4-band) 3–4 m high-spatial-resolution data. This one-on-one comparison between hyperspectral DESIS vs. high-spatial-resolution Dove data revealed that the hyperspectral images outperformed the high-spatial-resolution data. However, the sub-field measurement capacity of the Doves cannot be replicated by DESIS.

### 9.3. The Hyperspectral Imager Suite (HISUI)

**HISUI** was added to the ISS from the Japanese Space Agency (JAXA) and Japanese Ministry of Economy Trade and Industry (METI) and launched in December 2019 and released for operations after a lengthy checkout in September 2020 [251]. Its spatial resolution is somewhat smaller than DESIS, with 20 m × 30 m pixels, a 20 km swath, a 63-day repeat cycle and a 3-day repeat with pointing capability (Table 14). As with DESIS, one of the main objectives of this program is to obtain practical development and application experience to fly a full-scale mission. 

The HISUI has two spectrometer subsections, one detector covering the VNIR region of 400–900 nm at 10 nm spectral resolution and a SWIR detector covering wavelengths at 12.5 nm resolution between 895 and 2481 nm (https://www.jspacesystems.or.jp/en/project/observation/hisui/ (accessed on 27 October 2023)). It has 185 spectral bands with 12-bit dynamic range and a relatively high SNR of >450 @ 620 nm and >300 @ 2100 nm. HISUI collects data from 200 globally distributed sites that are up to 100 km^2^ in size. The maximum daily data collection is 690 GB/day. Scientific collaborators can request observation coverage and priority downlinks, while other science users can request archived data from the HISUI project. HISUI has closed research applications for data collection priorities. 

The HISUI project will distribute archive data to other science users. HISUI ranks their processing levels consistent with other data providers: Level 0 is raw data; the Level 1A data have radiometric calibration coefficients applied to the raw data, with no spatial resampling. Level 1R product provides Top-of-Atmosphere (TOA) spectral radiances, with no spatial sampling applied. Level 1G includes geometric correction and orthorectified TOA radiance, for which parallax correction and keystone properties are considered. Spectral continuity between the separate VNIR and SWIR collections are also accounted for. The Level 2G product provides atmospherically corrected surface spectral reflectance, generated from L1G data with QA information. Their L-2G product is generated for science research purposes but is not validated by the program.

### 9.4. The Earth Surface Mineral Dust Source Investigation (EMIT)

**Figure 18:**
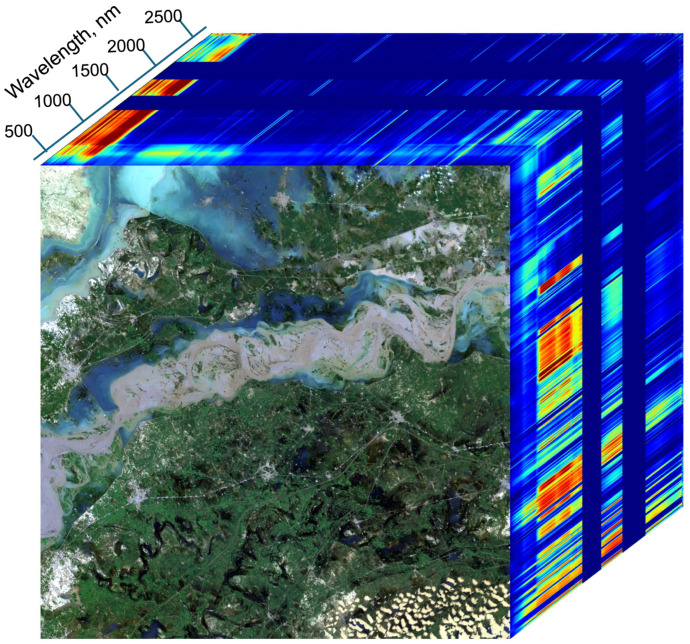
A spectral data cube is a visual representation of the spatial and spectral complexity of imaging spectroscopy data. This data cube is from the flooding that occurred in Pakistan in 2022 that began in mid-June following monsoon rains and melting Himalayan glaciers that began in the north and extended flooding downriver to October. This EMIT example shows a mix of surface materials in “true color”. The front-facing image shows a turbulent river running across the middle, with lots of sediment in the main body but less turbid flooding waters around the edges. A second flooding stream is seen in the upper left of the image and numerous narrow black curving lines of smaller flooded channels. Roads are easily seen as thin lines connected to villages and cities (gray to white colors) and scattered thin clouds (white) in the lower right corner of the image. Much of the vegetated area along the right side of the image shows typical vegetation spectral pattern with low reflectance (% of incident sunlight) in the first bands (dark blue) that are in the visible bands and are low due to plant photosynthesis, followed by a sharp increase in reflectance at the boundary of the visible and near-infrared wavelengths. The two belts in the middle of the spectra are wavelengths that are absorbed by the atmosphere and no light reaches the land surface, so they are removed from the dataset. The sides of the data cube show the reflectance of pixels along the top and right edge of the facing image and the wavelengths are shown on the left corner. The reflectance values are coded following the color pattern of a rainbow, from lowest reflectance in black/dark blue to highest reflectance in red. The wavelengths are shown in the upper left (Z-direction), starting from the shortest wavelengths ~380 nm to the longest at 2500 nm. (Image from EMIT project website at https://earth.jpl.nasa.gov/emit/news-events/news/ (accessed on 14 May 2024)).

**EMIT** was launched in July 2022 and is the third IS currently hosted on the ISS. It is a NASA Earth Ventures Instrument (EVI-4), a Principal Investigator instrument built at the Jet Propulsion laboratory/(https/www.earth.jpl.nasa.gov/emit/science/objectives/ (accessed on 8 December 2023)) and is a prototype for the SBG-VSWIR mission later this decade (Table 14). EMIT has a UV-SWIR spectral range (380–2500 nm) 286 bands with 7.4 nm spectral sampling, for images collected across a swath 75 km wide (https://space.oscar.wmo.int/instruments/view/emit/ (access 14 May 2024)), [45]. EMIT data have 300 spectral channels that are produced as calibrated, geo-rectified, and atmospherically corrected data cubes 60 m^2^ (Figure 18). An initial download of ~20 spectrally diverse datasets were released [252] and the first large, downloaded group of data cubes to the scientific community occurred in the fall 2023; another data release is scheduled for summer 2024. EMIT has successfully mapped many surface minerals and methane sources [252,253,254] and the data is available for researchers from all disciplines of environmental sciences to access and use. 

At the peak of flooding about 84,952 km^2^ were flooded based on a Fact Sheet #8, 30 September 2022 from USAID (http://www.usaid.gov/ (accessed on 15 May 2024)). Flooding killed at least 1739 people with more than 12,867 injured, and more than 2.1 million people left homeless. The estimated cost of reconstruction and economic damage was at least $30 B. EMIT or future imaging spectrometers (and other satellites) in space can provide more detailed information about the conditions about the specific natural hazard e.g., during floods, earthquakes, landslides, volcanic eruptions, and many others. Improved access to rapid knowledge about disasters could guild management options, especially in high-risk areas or for disasters such as this (three months of flooding) to help communities and first responders prepare and alleviate some of the devastation.

Originally approved for a one-year mission, due to its outstanding performance and results, EMIT has been approved for an extension on the ISS until at least mid-2026, and perhaps longer to support the SBG-VSWIR instrument. EMIT design was based on an earlier cubesat, the Snow and Water Imaging Spectrometer (SWIS) [255] and itself also represents a prototype for the future SBG-VSWIR mission. EMIT is a Dyson spectrometer with a 2-mirror telescope that focuses light on the slit and passes through a calcium fluoride crystal refractive element to the concave grating with structured blaze (created by electron beam lithography), optimizing diffraction efficiency over the full spectral range (https://earth.jpl.nasa.gov/emit/instrument/specifications/ (accessed on 8 December 2023)). After being dispersed into the spectrum by the grating, light passes back through the CaF2 block to the order-sorting filter and detector array (see Figure 1 for a schematic). The system’s electronics receive, amplify, and digitize the weak analog signals from the detector array, producing a high data rate, which is compressed and stored on a digital recorder for replay to the ISS and subsequent transmission to the ground. EMIT has a sophisticated thermal control system for measuring the SWIR wavelengths and for opto-mechanical stability. Heat generated within EMIT is radiated to space to maintain thermal balance.

**Figure 19:**
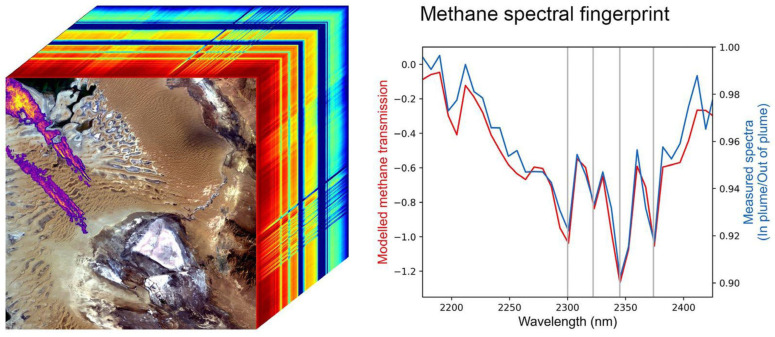
The figure on the left shows an EMIT image cube with two mostly parallel streaks, here mostly purple but with some orange and yellow colors within. These are plumes of methane in the atmosphere. The two methane plumes are colored in order of increasing concentration intensity from purple, orange, and yellow. Data from EMIT acquired over Turkmenistan. The corresponding graph on the right shows a pixel spectrum from the plume measured by EMIT in blue and a reference spectrum of methane in red. (Images and data from EMIT project site at earth.JPL.NASA.gov/EMIT/ (accessed on 3 May 2024)).

EMIT is the only instrument specifically designed and optimized to measure the spectral properties of exposed mineral surfaces in the deserts of the world [256], including sources of wind-blown dust particles that are blown into the atmosphere [256] The complex mineralogy of different source materials, these wind-borne dust particles can have significant impacts on heating or cooling of the atmosphere depending on particle types, which causes uncertainties in the Earth’s radiation budget. Because Earth’s deserts are located within the latitude range it views (52° N to 52° S), EMIT collects data from all desert areas. Because it is a full range IS, all pixels can be analyzed and information about the atmosphere above the pixels. Figure 19 provides an example from EMIT’s detailed methane spectra and the area these materials were extracted from. The two most important greenhouse gases, carbon dioxide and methane have absorption features in the shortwave infrared (1600–2500 nm) that permit mapping them when they are present in the atmosphere. Although this technology is usually collecting spectra of materials on the Earth’s surface, the gases and particles that come between it and the satellite are also observed and can be measured, including carbon dioxide, methane, ozone, aerosols, and water vapor. In the example in Figure 19, two plumes of methane were measured over Turkmenistan. The colors in the plume indicate the relative concentration of methane being measured, with the colors from lowest concentration detectable in purple to orange and yellow as the highest concentration. The identification of methane (or carbon dioxide in other samples) is identified by the spectrum (shown in the right panel) comparing a laboratory sample of methane in red with a spectrum from the plume in blue. It is easy to see that the shape of the satellite measurement follows the pattern of a laboratory sample, including the most important observation that the four absorption features between 2300 and 2375 nm line up by wavelengths, providing confirmation of the spectral signature of this gas.

### 9.5. The Orbiting Carbon Observatory (OCO-3)

The OCO-3 was developed by NASA/JPL to quantify sources and sinks of CO_2_ (https://ocov3.jpl.nasa.gov/ (accessed on 7 November 2023)) of natural and anthropogenic sources from terrestrial and ocean ecosystems (Table 11). OCO-3 weighs 50 kg and was built from spare parts of OCO-2 and draws 600 W of power from the ISS (https://ocov3.jpl.nasa.gov/mission/quick-facts/ (accessed on 15 May 2024)). It was launched in May 2019 for an 8-year mission (through 2026) and was robotically installed on the JEM Exposed Facility Unit 3 (Table 14). It is a non-imaging multiband grating spectrometer that measures light reflected from gasses at three wavelengths: 765 nm (O_2_-A absorption band), 1610 nm (weak CO_2_ absorption), and 2060 nm (strong CO_2_ absorption). At a GSD of 4 m^2^, OCO-3 can acquire measurements at finer spatial scales than the polar-orbiting OCO-2 and collects data simultaneously from eight terrestrial system blocks of 100 km^2^. Given its band location at 765 nm, which is near the ~760 nm O_2_-A feature, OCO-3 also measures far-red fluorescence in the declining tail of the fluorescence spectrum beyond the ~740 nm far-red peak. The ISS, because of its 51.6° orbit, is a good location for this instrument since it covers the most densely habited portion of the globe and provides a focus for improving understanding of trace gas emissions from cities and other sites of human activities globally.

### 9.6. ECOsystem Spaceborne Thermal Radiometer Experiment on the Space Station (ECOTRESS)

With pixel sizes slightly larger than two Landsat images (69 m × 38 m), **ECOSTRESS** acquires the most detailed temperature images of the Earth’s surface currently available from space. It is also the prototype for the SBG-TIR to be launched around 2028 in partnership with the Italian Space Agency, ASI. ECOSTRESS was launched to the ISS in 2017, the primary mission for ECOSTRESS is to evaluate how the terrestrial biosphere is responding to changes in water availability, particularly for agriculture (ecostress.jpl.nasa.gov/news/8nasa-fact-sheet/ (accessed on 3 May 2024)). Its mission has been extended to at least 2026. It has proven to be well suited for examining changes in water budgets at regional scales, for monitoring changes in soil moisture and vegetation moisture content, and for collecting data to estimate evapotranspiration [257,258,259,260]. Fisher et al. [261] were able to separate and map open water evaporation from ET and mixed sites. Doughty et al. [262] used ECOSTRESS to determine tropical forest stress levels and their sites were found to approach critical temperature thresholds. Diurnal patterns per month for evapotranspiration and water stress can be estimated by combining the thermal signals acquired at different times of the day over a period of several weeks. ECOSTRESS is retrieving good estimates of emissivity from its thermal bands, as compared with the ASTER instrument on the Terra satellite [263]. ECOSTRESS takes advantage of the temperature differences in pixels of well-watered agricultural crops versus those of temperatures of dry vegetation or bare soils, to bound temperatures for minimum and maximum evapotranspiration.

**Figure 20:**
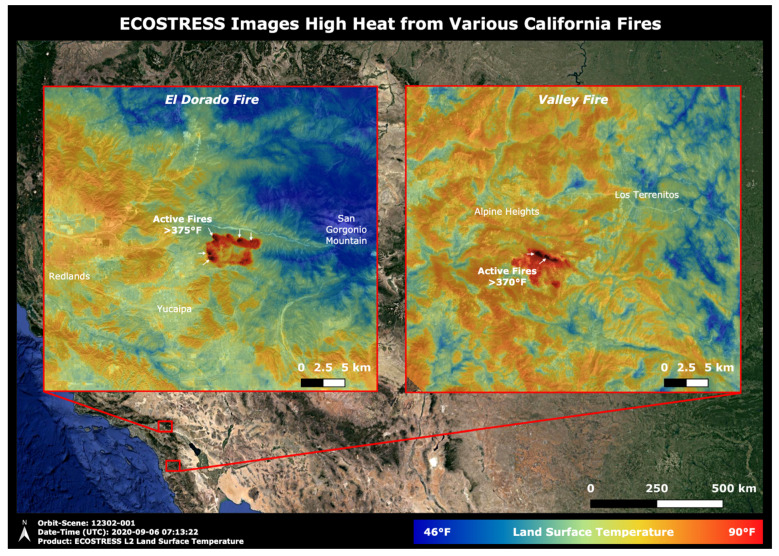
ECOSTRESS data shows high surface temperatures (in Fahrenheit degrees), from two wildfires, the El Dorado (left enlargement) and Valley (right enlargement) in southern California, captured in the evening at 00:13 (local time) on 6 September 2020. The high nighttime surface temperatures ranging from 7.8 °C (46 °F in color legend) to 32.2 °C (90 °F) are abnormally warm due to an ongoing heat wave in the region. The white arrows at points along the edge of the fire boundary show locations of 375 °F (190.6 °C) temperatures (El Dorado fire) and points within the Valley Fire of 370 °F (187.8 °C). Images reveal large temperature variations across the enlarged images due to both topography and vegetation cover (type and amount). Drainage patterns are easily revealed in shades of blue and small roads are near white in color. Image from https://ecostress.jpl.nasa.gov/gallery/wildfire/viewgalleryimage2/ (accessed on 14 May 2024).

Increases in precipitation due to climate warming are predicted by mid-century, with precipitation extremes (both high and low) intensifying in a warmer climate [264,265,266] and amplifying differences at regional scales between dry and wet regions [267,268,269]. Figure 20 illustrates ECOSTRESS’s ability to monitor fire temperatures and background temperatures in two fires in southern California. Since the 2018 launch, Google Scholar identified 20,700 search results on “ECOSTRESS”. listing various subjects including maps and forecasts for drought, thermal pollution, mosquito-borne diseases, agriculture, mangrove, surface water temperature and biodiversity. 

ECOSTRESS is a five-band thermal imaging spectrometer (https://ecostress.jpl.nasa.gov/instrument/ (accessed on 12 November 2023)) with bands in the 8–12.5 μm range, plus one band centered at 1.6 μm for geolocation and cloud detection (Table 14). It was built on a prototype designed for HyspIRI (a recommended instrument by the 2007 NASEM Decadal Survey [122], the space-ready airborne Prototype HyspIRI Thermal Infrared Radiometer (PHyTIR) [270,271]. It has a double-sided scan mirror, rotating at a constant 25.4 rpm to allow the telescope to view a 53°-wide nadir cross-track swath, focusing the optical signal onto the 65 K focal plane, which is cooled by two commercial cryocoolers (Thales), containing a custom 13.2 μm-cutoff mercury–cadmium–telluride (MCT) infrared detector array. Heat rejection for the ECOSTRESS cryocoolers and electronics is provided by the cooling fluid loop on the JEM External Facility, where it is deployed at Site 10 (one of the two end locations).

### 9.7. The Global Ecosystem Dynamics Investigation (GEDI)

The GEDI instrument on the ISS is a NASA and University of Maryland collaboration. GEDI’s scientific goal is to map the biomass of forests globally, to obtain data critical to achieving more accurate quantification of the global carbon budget, and for reducing uncertainties in estimates from carbon budget models [272]. GEDI is a prototype for a possible NASA Explorer mission to monitor 3D vegetation structure. Changes in biomass due to harvest, wildfire, or mortality due to disease or other causes are important uncertainties to the global carbon budget. Carbon emissions from deforestation (2016–2020) are estimated to be ~2.9 Gt CO_2_/year, about the same amount as forests sequester, ~2.5 Gt CO_2_/yr [272] both values are associated with large uncertainties. A global GEDI-like lidar system would greatly reduce these uncertainties. 

The GEDI is a full-waveform geodetic-class light detection and ranging (lidar) instrument (https://gedi.umd.edu/ (accessed on 15 May 2024)) that emits and then measures the reflected light returned in the NIR wavelength of 1064 nm (Table 14). The system is comprised of three lasers that produce eight parallel tracks of observations. Each laser fires 242 times/s and illuminates a 25 m footprint on the Earth’s surface. Each footprint is separated by 60 m along track, with an across-track distance of approximately 600 m between each of the eight parallel tracks. Full-waveform Lidar is essential for quantifying the 3D surface structure, such as the vertical distribution of vegetation, by recording the amount of laser-pulsed energy that is returned from plant material at different heights above the ground. From GEDI waveforms, four types of structure information can be extracted: surface topography, canopy height metrics, canopy cover metrics, and vertical structure metrics. Types of studies include mapping forest height [273], crown dimensions [274,275], forest gap structures [276], and habitat information [277].

**Figure 21:**
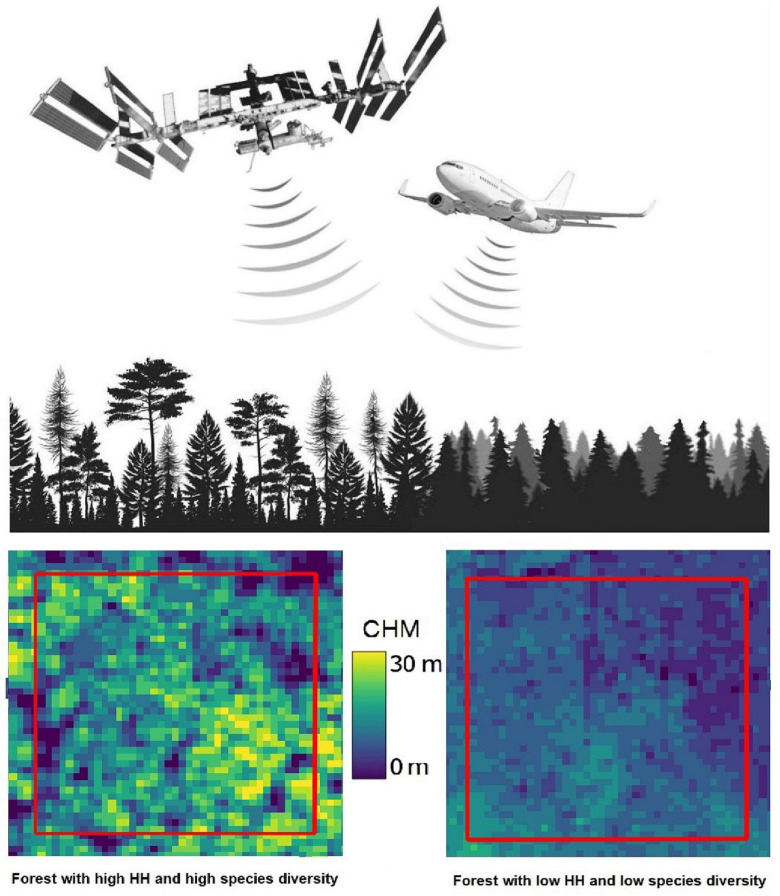
One of the most urgent concerns of biologists is to better identify and map locations with high biodiversity before they are lost. The GEDI lidar imager on the ISS has found that the total height heterogeneity (HH) occurs in areas with high biodiversity. The boxes show the areas analyzed were the same size and the location shows similar patterns inside and outside the analyzed area. Their study acquired GEDI data for two forested sites in Germany with different 3D structures and numbers of plant species. The study supported the hypothesis and is consistent with patterns found in airborne data at higher spatial resolution. Reproduced from Torresani et al. [278] with permission.

One of the most urgent concerns of biologists is to better identify and map locations of high biodiversity before they are lost. The GEDI lidar imager on the ISS has found that the total height heterogeneity (HH) occurs in areas with high biodiversity. A recent study by Torresani et al. [278] tested the hypothesis that the total height heterogeneity (HH) occurs in areas with high biodiversity. The study acquired GEDI data for two forested sites in Germany with different 3D structures and species, with which they evaluated the “Height Variation Hypothesis” that states that higher vertical complexity (HH) should increase the number of sub-habitats and niches, thus increasing species diversity. Figure 21 shows a schematic from their paper with the GEDI lidar data from two types of forests, a forest of diverse three-dimensional structure on the left and a more uniform forest structure on the right, with trees in both plots approximating the same average height. Their figure demonstrates the concept that variation in height diversity between these two sites, while independent data verified their differences in species richness.

The NASEM 2017 Decadal Survey [107] recommended NASA consider an observing priority for a mission focused on the “3D structure of terrestrial ecosystem including forest canopy and aboveground biomass and changes in aboveground carbon stock from processes such as deforestation and forest degradation”. It was grouped with seven other priority missions for further review and consideration as Explorer missions, but as of 2024, NASA has not released a competitive call for proposals.

GEDI was launched on Dec. 2018 and deployed on the JEM Exposed Facility. It was originally slated to remain on the ISS for only two years, but extended to January 2023. After an appeal by forestry experts the mission has been extended, but with a gap of approximately 18 months until fall 2024. The refrigerator-sized instrument is in storage on the ISS while another project completes its mission at that GEDI site on the JEM. GEDI produces approximately 5 billion cloud-free observations per year. Version 2 data products have been released to the public via the ORNL DAAC (https://daac.ornl.gov/cgi-bin/dataset_lister.pl?p=40/ (accessed on 28 January 2024)) and LPDAAC (https://lpdaac.usgs.gov/product_search/?query=GEDI+data&view=cards&sort=title/ (accessed on 28 January 2024)).

The 2017 NASEM Decadal Survey [279] recommended NASA consider an observing priority mission focused on the “3D structures of terrestrial ecosystem including forest canopy and aboveground biomass and changes in aboveground carbon stock from processes such as deforestation and forest degradation”. It was grouped with seven other priority missions for further review and consideration as Explorer missions, but as of 2024, NASA has not re-leased a competitive call for proposals so there is no decision yet on a future GEDI-like mission.

## 10. Commercial Sector: High-Spatial-Resolution Multispectral Imagers and Imaging Spectrometers

Small satellites (aka, smallsats) and drones carrying imagers are typically built and flown by academic institutions or commercial companies for special purposes such as detailed mapping of experimental sites, agricultural fields, or forests. Most high-spatial-resolution satellites, e.g., with 5 m or smaller pixel sizes are commercial. These are often the most interesting to users who have high priority for spatial detail and do not require as many spectral bands. Data from these satellites are often expensive and details about calibration, radiometric resolution, and changes in radiometry over time are not always available. However, high-spatial-resolution data are typically utilized for their spatial detail for which there are minimal requirements for radiometric accuracy. The small images can be aggregated through 2D mosaics of adjacent images. But because of their small swath-widths, available data often have infrequent coverage and conditions on the ground may have changed when mosaicked. One solution for developing larger areal coverage is for commercial companies to fly multiple instruments for better temporal sampling, e.g., as is being performed by Planet’s Flocks of Doves. 

The imagery often has interpretable spatial patterns within the field scale. Analyses are often simple 3-color composite images or statistical methods like NDVI and a focus on GIS tools. These data are frequently used for validating coarser imagery but there is increasing interest to directly address environmental problems. Users are often familiar with GIS programs and use tools in commercial and open-source software to analyze the imagery. The number of satellite systems operating in this category has decreased in recent years as instruments/satellites have aged out of production without new ones replacing them. The main exception to this is the new Planet (formerly Planet Labs) satellites, which have been expanding their constellations.

### 10.1. The Satellite Pour L’Observation de la Terre (SPOT) 6/7

The SPOT 6 and **7** satellites were launched in 2012 and 2014 for 10-year lifetimes (https://eos.com/find-satellite/spot-6-and-7/). These are 4-band multispectral imagers with 2 m GSD and a panchromatic band at 1.5 m (Table 15). The swath is 60 m and the instruments are in a 679 m altitude orbit with a morning crossing time. They were widely used for ecological research, e.g., avalanche mapping, eelgrass habitat, agricultural capabilities, sugar cane yield prediction, groundwater recharge, and both above and belowground biomass regrowth in a reforested site (Google Scholar identifies 156,000 documents). Spot 7 was recently terminated in March 2023 and only SPOT 6 is still collecting data but is near its end-of-life. Archival data are available as are requests for new data collections. ESA Access is freely available to users with submitted and accepted proposals (https://earth.esa.int/eogateway/catalog/spot-6-to-7-full-archive-and-tasking-and-spotmaps-2-5-dataset/ (accessed on 15 February 2024)). Level 1a, level 1b, level 2a, level 3, multiple products are available at: https://earth.esa.int/eogateway/catalog/spot-6-to-7-full-archive-and-tasking/ (accessed on 15 February 2024). Archived data for SPOT 7 are available online and the data catalogue and requests for new data are available for SPOT 6. The ESA Airbus Defense and Space site has data for SPOT.

### 10.2. Pléiades 1A and 1B Satellites

These satellites were managed as a constellation of two, launched in 2011 and 2012 with five-year life expectancies, acquiring high-spatial-resolution images. They have four multispectral VNIR image bands at 2 m GSD and a VIS panchromatic band at 50 cm resolution (Table 15). As with the SPOT data, the ESA Services Portal requests a proposal and registration to access data from the ESA at https://earth.esa.int/eogateway/catalog/pleiades-full-archive-and-tasking/ (accessed on 26 March 2024); Pléiades missions (https://earth.esa.int/eogateway/missions/pleiades#instruments-section/ (accessed on 12 February 2024)).

### 10.3. Pléiades NEO

This is a newer four-satellite constellation acquiring very high-spatial-resolution images (Table 15). These four satellites are oriented in cardinal directions and have unprecedented capability to map at fine detail: 30 cm panchromatic data and 1.2 m multispectral imagery data. Each of the four smallsats in this constellation has six multispectral VNIR bands. The first pair was launched in 2020 and the second pair in 2022, with a 10-year life expectancy, and each has a 26-day repeat cycle. As with the previous examples, ESA requires users to submit a proposal and register to have access to the data at the ESA Services Portal; data are available at https://earth.esa.int/eogateway/catalog/pleiades-neo-full-archive-and-tasking/ (accessed on 12 February 2024). The “OneAtlas” portal provides access (https://oneatlas.airbus.com/home). Pléiades-Neo (https://earth.esa.int/eogateway/missions/pleiades-neo#instruments-section/ (accessed on 12 February 2024)).

### 10.4. TanDEM-X

The TanDEM-X is a commercial radar satellite system from the DLR, with data available from Astrium/Infoterra, part of the European Aeronautic Defense and Space Company (EADS). TanDEM-X is a TerraSAR-X SAR with added capability to produce a digital elevation measurement (Table 15). The system is a series of four all-weather land observing satellites, with X-band (~2 cm, 9.65 GHz) SAR imagers with a design life of five years. Each satellite includes a Tracking, Occultation and Ranging (TOR) limb-scanning system that measures the phase delay due to refraction during occultation between GPS and LEO. Real-time occultation data are from UCAR (https://cdaac-www.cosmic.ucar.edul (accessed on 4 March 2024)) to provide temperature and humidity sounding adjustment for the SAR, with the highest vertical resolution for space weather.

There is a planned advance in capacity in 2028 (Tandem-L Interferometric Radar Mission) to include two units flying in parallel to provide interferometry data [279]. These will be L-band (~25 cm, 1.27 GHZ) SAR imagers. They are expected to be dual-polarization (HH or VV) in the large swath mode, with 350 km swath and 7 m GSD. The second mode is narrow swath, which will have a 175 km swath, also with 7 m GSD and Quad polarization with the HH, HV, VV, and VH modes.

### 10.5. Planet Scope Constellations

One solution for obtaining better temporal sampling is to launch multiple copies of the same satellite instrument, offset in time. This is the model for Planet’s most sophisticated 200+ SuperDove satellites now in orbit (https://www.planet.com/products/monitoring/ (accessed on 4 March 2024)), with a high spatial resolution of 3.7 m. These commercially supported satellites provide eight simple VNIR bands, following a “smaller, cheaper, faster” model, as advocated by NASA in the early 2000s.

Planet Labs was founded in San Francisco, California in 2010. Its name was shortened to “Planet” and it has built and successfully deployed more than 452 smallsats with approximately 130 currently in orbit, collecting over 200 M km^2^ of imagery daily (https://developers.planet.com/docs/data/planetscope/ (accessed on 4 March 2024)), with a global inventory of 1300 images over all of Earth’s land masses (https://earth.esa.int/eogateway/missions/planetscope/ (accessed on 4 March 2024)). The first Doves, “Dove Classic” (Planet PS2) had three wide spectral bands (RGB) and later, Dove R (Planet PS2.SD) had four VNIR bands. First launched in 2014 and each weighing approximately 5 kg—often referred to as “cubesats” though they are somewhat larger, the Doves are launched in batches (termed “flocks”). The oldest Doves were launched in 2015 at an altitude of 450 km and GSD of 3–5 m; individual scenes had 24 × 8 km^2^ footprints, and the next generation had 24 × 16 km^2^ footprints. Approximately 209 Dove R’s have been flown (Table 15), with a three-year life expectancy.

The first 20 SuperDove (Planet SPB.SD) imagers were launched in Flock 4a (in April 2019) at 504 km altitude into a sun-synchronous morning crossing orbit. As of mid-2023, approximately 195 have been launched (Table 15). SuperDoves have eight spectral bands in the VNIR and 3 m GSD; scenes are 62.5 × 19.6 km^2^. Globally distributed data are acquired daily, with some sub-daily coverage. The eight SuperDove bands also match six of Sentinel-2’s bands: Planet’s Blue bands (1&2) match S-2 blue bands 1&2; Planet’s red band (B6) matches S-2’s B4; Planet’s Red Edge B7 matches S-2’s B5; and Planet’s NIR B8 matches S-2 B8a. The Planet bands 3 and 5 (Green 1 and Yellow) have no equivalent S-2 bands. By combining environmental data with Planet and Sentinel-2 data [280] obtained improved wheat yield estimates. After training their model, they found that Planet data had the highest accuracy. Whether this result from Planet was due to its green and yellow bands or to its higher spatial resolution was not resolved.

SkySats are Planet’s third constellation, first flown in 2013 and now numbering 21 high-resolution satellites that image at 50 cm GSD (i.e., pixels). With 21 satellites (Table 15), they can revisit any location on Earth up to 10× daily with a daily collection capacity of 400,000 km^2^/day. SkySat satellites have a propulsion system to retain altitude, and three cameras that measure overlapping strips for scenes approximately 1 × 2.5 km^2^. They also capture stereo and video data for up to 90 s. Sixty SkySat scenes along an imaged flight strip are orthorectified into a flight segment measuring approximately 20 × 5.9 km^2^. 

Planet acquired the RapidEye constellation of five satellites in 2015 and retired them in 2020; each had a multispectral imager (MSI) of five bands and a 5 m GSD (Table 15). The RapidEye constellation was originally owned/operated by BlackBridge, a German satellite imagery and data provider. Launched in 2008, RapidEye was the first fully commercial class EO constellation of high-spatial-resolution imagery (https://earth.esa.int/eogateway/missions/rapideye/ (accessed on 4 March 2024)). When Planet retired that constellation in 2020, it held one of the largest archives of 5 m MSI VNIR imagery. RapidEye image scenes ranged from 75 × 50 km^2^ to 75 × 300 km^2^. The online catalogue Planet Explorer provides options to search and browse for past data over selected areas and times. The ESA has a full archive of RapidEye data (https://earth.esa.int/eogateway/catalog/rapideye-full-archive/ (accessed on 4 March 2024)).

Two imaging spectrometers are scheduled for launch in 2024, one is a non-profit public–private partnership with Planet (possible launchrs in July 2024) and one from the NGO Environmental Defense Fund (launched in March 2024). These are included in the 2024 satellite launches, in Section 13.

## 11. Second and Third-Generation Geostationary Satellites

The GEO orbit is generally flown above the equatorial plane at an altitude of >36,000 km, which provides wide coverage and high-frequency repeat measurements over equatorial areas of approximately 20–25% of the Earth’s surface. These satellites (Table 16) operate in different modes representing different geographic domains, down to regional extent. Consequently, it requires a network of equatorial satellites, generally five or six satellites are sufficient to obtain global coverage across all longitudes and pole to pole. The advantage of this orbit is the ability to make repeat measurements every few minutes (or even faster, depending on their mode of operation, e.g., a major disaster or storm). A disadvantage is the relatively course spatial resolution of the data, measured in kilometers compared to meters.

GEO instruments include both active and passive instruments, both imaging and non-imaging sensors, e.g., sounders, that are principally flown to monitor weather events, including measurements of temperature, water vapor, CO_2,_ and ozone and other atmospheric gases (https://www.nesdis.noaa.gov/current-satellite-missions/currently-flying/geostationary-satellites/ (accessed on 6 March 2024)). The current (second) generation of NOAA platforms includes GOES 16 (GOES East) and GOES 18 (GOES West) launched in 2014 and 2022, respectively (https://www.nesdis.noaa.gov/our-satellites/currently-flying/goes-18-launch (accessed on 6 March 2024)). They carry the Advanced Baseline Imager and the Geostationary Lightning Mapper (GLM), in addition to four instruments for characterizing the space environment, e.g., monitoring solar flares and ejection sources, charged particle dynamics, and monitoring solar and X-ray irradiances in the upper atmosphere.

These measurements are typically flown for weather, atmospheric composition, and climate or for oceanographic research and are less commonly used in environmental research. Each satellite contains several instruments for measuring atmospheric conditions. Of these, the passive GEO imagers, because of their ability to measure diel and diurnal temporal patterns [281] or simply provide daily and sub-daily measurements, are of increasing interest for certain types of environmental research, e.g., monitoring diurnal evapotranspiration, heat islands [282] or wildfire conditions [283,284], and for estimating evapotranspiration [285]. They also provide more detailed daily measurements over a growing season [286] over multiple years at spatial scales that compare to MODIS data. The newest generation of GEO satellites represents a coordinated effort among nations to launch these satellites carrying significantly more capable instruments to monitor and track weather systems as they move around the globe [287]. The satellites in this new generation have spatial resolutions that are at the scale of older “moderate” environmental satellites.

Previous generations of geostationary satellites were of little interest to environmental researchers, land managers, and oceanographers, for many reasons, primarily because of the course resolution of the data, and difficulties with accessing and processing the data, in addition to and the cost of data. However, that has changed with the NOAA Advanced line Imager (AHI) and the JAXA Advanced Himawari Imager (AHI) (Table 16). These satellites have a red band at 500 m GSD, NIR bands at ≤1 km GSD, and TIR bands at 2 km GSD, giving them spatial resolutions like those of global mapping polar satellites. They also share band placement for 15 of 16 bands from visible through TIR. These data are available at 10 min intervals for the full disk (depending on the mode recorded for the disk of the earth (pole-to-pole) or a smaller area. These data, along with standard products and training tools to aid new users in accessing the data, enable them to obtain (for the first time) information about short term environmental changes at routine moderate spatial resolution. These data also provide the possibility of very high temporal resolution for environmental emergencies (fires, floods, toxic plumes, etc.), as frequently as every minute. 

### 11.1. The Advanced Himawari Imager (AHI) and the Advanced Baseline Imager (ABI)

The Advanced Himawari Imager (AHI) instrument on the Japanese Himawari 8 (launched in 2014) and 9 (launched in 2016), are the first of the new series of geostationary satellites (Table 16). The Himawari 8 and 9 only carry the AHI and a communications/data service transmission capability.

The Advanced Baseline Imager (ABI) on the US GOES 16 (launched in 2016) and 18 (launched in 2022). The GOES 17, launched in 2018 but because of a failure in thermal channels it was replaced by GOES 18 in 2022 (Table 13). The AHI has 15 of 16 passive spectral bands (also termed channels) that are shared with the GOES ABI. Each has one unique band (ABI, 1378 nm; and AHI, 510 nm; [288]. Both satellites have a red band (640 nm and 645 nm, respectively) with 500 m GSD. Both also have a 1 km GSD blue band at 470 nm and 455 nm, respectively, NIR bands at 865 nm (ABI) and 860 nm (AHI), and both have a 1 km GSD band at 1610 nm. In the SWIR, ABI has a 2250 nm and AHI has a 2260 m band, both at 2 km GSD. In the mid-IR, ABI has three bands at 3.90, 6.19, and 6.90 µm, while AHI has bands at 3.85, 6.25, and 6.95 µm, all at 2 km GSD. They both have seven bands in the TIR at 2 km GSD: ABI (7.34, 8.5, 9.61, 10.35, 11.2, 12.3, 13.3 µm) and AHI (7.35, 8.60, 9.63, 10.45, 11.20, 12.35, 13.30 µm). The 500 m red bands and 1 km VNIR are near the spatial resolution range of VIIRS bands and other polar satellites, but with the added benefit of a high-density time series with more than a hundred images acquired each daylight period. These GEO satellites operate in different spatial coverage modes. For the NOAA GOES, the full disk of the Earth is available every 10 min, CONUS (for the continental USA area), at a frequency of every 5 min, and a mesoscale or the high-resolution mode, typically used in response to disasters like a wildfire or flood or a major storm, at 30 or 60 s intervals.

With the improved spatial resolution and increased number of bands, the GEO satellites have started to receive attention of environmental scientists [289]. Clearly the standard ~10 min-updated imagery can be used to monitor emerging hazards and natural disasters, e.g., floods, fires, earthquakes, landslides, hurricanes and tornadoes. There are high-resolution modes that image as frequently as a minute or even fractions of a minute. The data also can monitor natural environments to better understand possible early stress conditions from extreme temperatures and droughts. Data can be summarized to obtain daily or hourly information on net radiation, albedo, minimum and maximum temperatures, vegetation indices like the Normalized Difference Vegetation Index (NDVI), vegetation moisture indexes that use NIR and SWIR bands. Temperature and greenness data can be used to estimate carbon sequestration and evapotranspiration. Similarly, there is sufficient data to advance diurnal soil moisture patterns. 

### 11.2. The Lightning Imagers on the GOES 16, 18 and the MTG-I1

This new instrument is likely to be of interest to environmental scientists: the Lightning Imager (LI) on the MTG satellites. The lightning imagers (Geostationary Lightning Mission, GLM) on the two GOES satellites were the first with this technology (Table 16), starting in 2016 [290,291,292]. The Lightning Imager (LI) on the MTG-I will, along with the GOES GLM, monitor lightning intensity above a background radiance per cell (8km x 8km) over a specified time interval (e.g., 5 min). These can be reported as total light intensity or as numbers of strikes/time. While these instruments are new, there is considerable interest in correlating lightning strike intensity or number with ignition sites of wildfires. The full disk of the Earth is imaged at 777.4 nm continuously at 2 ms frame rate resolution and the instruments record the number of flashes and measure intensity of triggered events (90% at 45° and 70% average over full image coverage area (https://space.oscar.wmo.int/instruments/view/li/ (accessed on 5 March 2024)).

**Figure 22:**
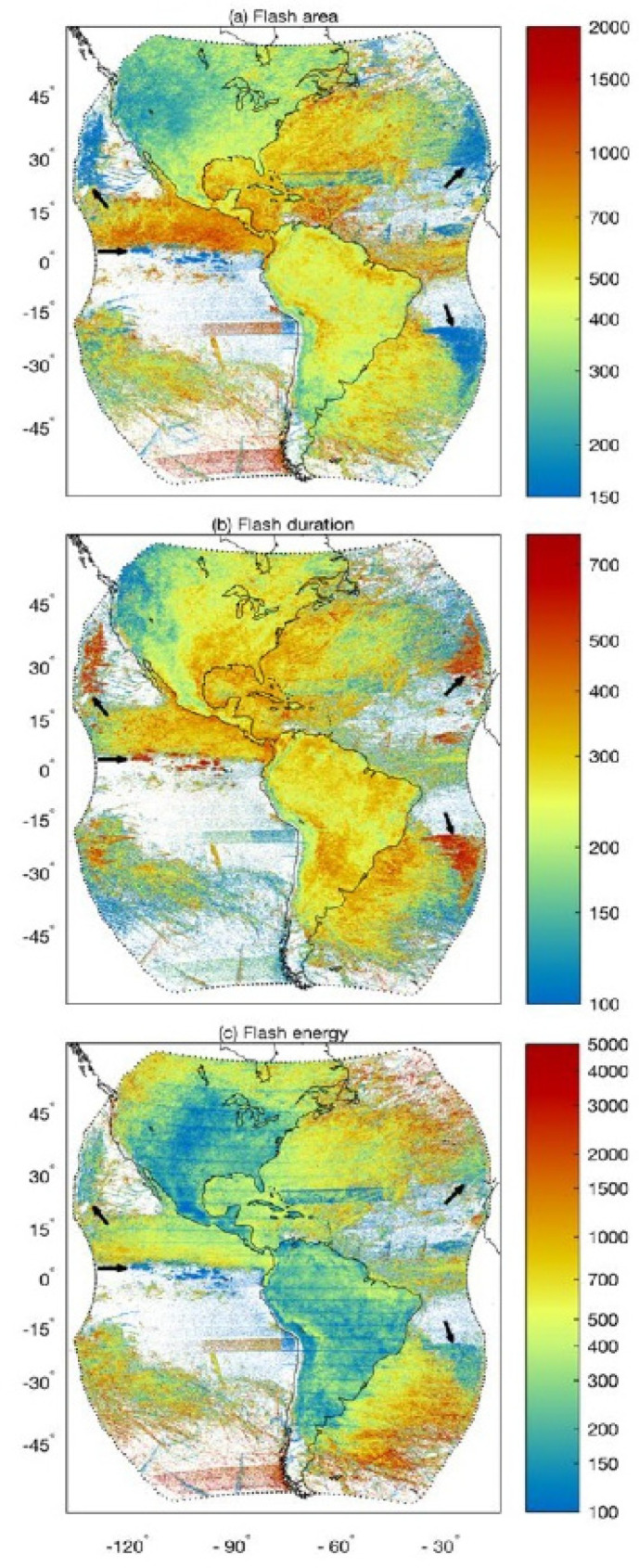
Some of the new capabilities on geostationary satellites (MTG-l1 and on the NOAA GOES 16 and 18) are the lightning strike data. NOAA’s Geostationary Lightning Mapper (GLM) data can be captured at different spatial scales and time periods. This figure shows mean GLM observations for: (**a**) flash area (km^2^), (**b**) flash duration (ms), and (**c**) flash energy (fJ). Arrows indicate data artifacts. Figure from Rudlosky et al. [290], reprinted with permission.

The GLM is first of its kind to continuously observe lightning from a geostationary orbit, throughout an area approximating a hemispheric field of view, and producing lightning data on unprecedented spatial–temporal scales. Figure 22 shows three types of data from the NOAA GLM lighting strike sensor. The panels show flash density per area, flash duration (generally higher over the ocean than the land), and flash energy. Lightning strike data will provide valuable health safety information during lightning storms and possibly lead to earlier wildfire detection.

Unlike the Himawari satellites, the GOES satellites carry multiple additional instruments for space weather and weather/climate monitoring. These are of lessor interest for EO research and applications but include the EXIS Extreme Ultraviolet Sensor, the GEOSAR for Search and Rescue, the SEM/Mag (magnetometer), SUVI Solar Ultraviolet imager, and a Space Environment In Situ Suite (SEISS) for Magnetospheric Particle Sensor (MPS), Energetic Heavy Ion Sensor (EHIS), and the Solar and Galactic Proton Sensor (SGPS).

### 11.3. EUMETSAT METOP Third-Generation Satellite-Imager (MTG-I)

**Figure 23:**
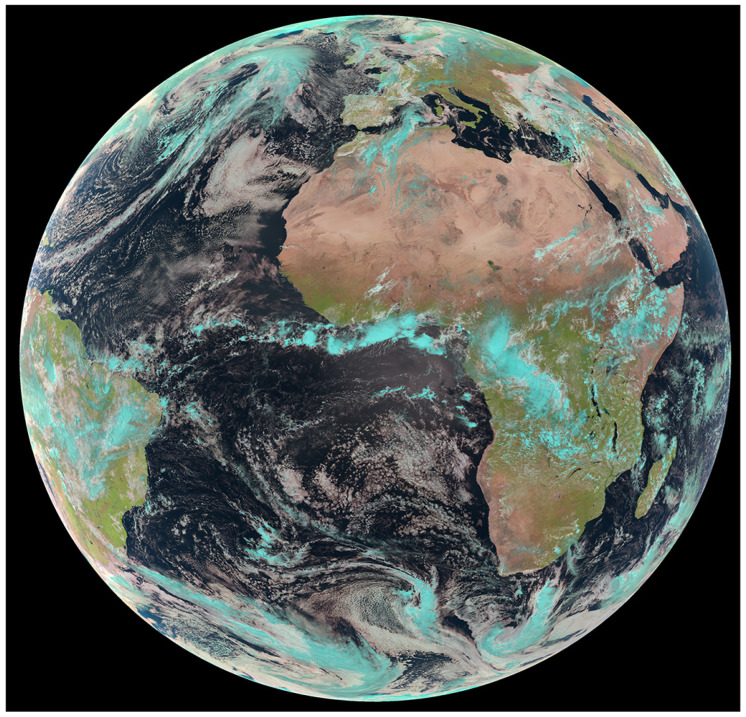
Spectacular first image on 4 May 2023, a full-disc Earth, from the new EUMETSAT geostationary satellite, MTG-I1. The Flexible Combined Imager (FCI) data produce revealing images in “true color” in remarkable detail, here for the Atlantic Ocean stretching from the Brazilian Amazon to Africa and the Middle East. The new system default mode receives a full disk scan every 10 min. Other modes provide more detailed spatial coverage over smaller areas (e.g., Europe) and more frequent coverage (figure from EUMETSAT/ESA (https://www.eumetsat.int/features/discover-first-images-mtg-i1/ (accessed on 15 May 2024)).

The Meteosat Third-Generation (MTG) represents the European Commission’s entry into a new 20-year era of Geostationary weather and climate modeling (Table 16) (https://www.eumetsat.int/meteosat-third-generation/ (accessed on 5 March 2024)). MTG will advance weather forecasting and nowcasting of dangerous storms in real-time for severe weather events. The new data are expected to contribute to greater accuracy in longer range forecasts, and the soundings will provide more detailed information on weather patterns for numerical weather prediction (https://www.eumetsat.int/meteosat-third-generation/ (accessed on 5 March 2024)). MTG is a complex and innovative geostationary satellite system that includes two imagers (designated MTG-I (1 or 2), and a sounder (MTG-S (1). The MTG-I is a new imaging system that can be operated in several spatial coverage modes, including a mode with the full disk of the Earth imaged every 10 min (Figure 23). A second mode is the Continental, a higher-resolution mode (comparable to NOAA’s CONUS coverage of the continental USA area, and comparable to “close looks” from the AHI and MTG) measured at a five-minute frequency, and a third mode is the mesoscale or high-resolution mode that is typically used in response to disasters like wildfires, floods, or major storms, imaged at 30 or 60 s intervals.

The first imager of this new series was launched in 2022 and the second is planned for 2026. The imagers will carry a new Flexible Combined Imager (FCI) on both platforms, to be followed by a second pair (I3 and I4) after 10 years. The second instrument of likely interest to the EO community is the Lightning Imager on the MTG-I1, which will provide information about extreme weather and wildfire hazards. The MTG-I1will also carry a GEO Search and Rescue capability, and a Radiation monitoring Unit (RMU) and a data collection service for transmission of data (also carried on the MTG-S1).

### 11.4. EUMETSAT MetOp Third-Generation Satellite Sounder (MTG-S)

The first imager in this series will launch in 2024 and it will carry the first **I**nfra**r**ed **S**ounder (IRS) placed in GEO, based on the IASI heritage. It will measure profiles in temperature, humidity, and wind profiles, and will measure them across four latitude belts. The northernmost belt (LAC4) includes Europe and is measured at 30-min intervals for a 6-h period. The next belt to the south (LAC3) will be imaged with two sequences of three images in 30-min intervals, separated by two hours. The next two latitudinal belts are LAC 2 and LAC 1 and they are measured in a sequence of three images in 30 min intervals, separated by five hours. Each dwell has a duration of 10 s and consists of 160 × 160 pixels (each 4 km × 4 km), for a total of 25,600 spectra in 1900 channels (spectral bands) per sounding. The IRS is a Fourier transform interferometer that will make these narrow band measurements in two bands, one in the midwave IR between 4.44 and 6.25 μm and the second in the band between 8.26 and 14.70 μm.

### 11.5. Sentinel-4 on the MTG-S-I

The second instrument on the MTG-S1 sounder is the Sentinel-4 (S-4) instrument, which will make measurements of atmospheric chemistry in GEO, complementary to Sentinel-5 (to be flown on the SG-1A polar-orbiting satellite). Its mission is to monitor key air quality trace gases and aerosols over Europe in support of the Copernicus Atmosphere Monitoring Service (CAMS) at high spatial resolution and with a fast revisit time [293]. The S-4 consists of a push-broom scanner (in east/wWest orientation) for imaging instruments that will measure the ultraviolet-visible and near-infrared and was designed to monitor key tropospheric air quality trace gases and aerosols at high spatial resolution at hourly increments (Table 13). The UVN is a 411-band imaging spectrometer that will make measurements in three ranges: UV, 305–400 nm (600 channels); VIS, 400–500 nm (600 channels); and the NIR, 755–775 (300 channels). This will allow frequent observations of air pollutants and greenhouse gases: BrO, CIO, H_2_O, HCHO, NO, NO_2_, O_3_, SO_2_, and total column HCOH, NO_2_, O_3_, and SO_2_ plus aerosols and cloud top heights.

## 12. There Are 90 EO Satellites Still in Orbit at the Beginning of 2024 for Science and Applications

At the start of 2024, we identified 90 named instruments hosted on 61 EO satellites still in orbit. Several of these satellites have already reached their expected lifetimes or will soon do so. Some have persisted for much longer than anticipated according to their design life and are still providing useful Earth System measurements. In this paper, we have focused on platforms/instruments with free and open data policies, and most of those we discuss are in this category. Our study represents a subset of the remaining ~150 satellites for science and applications in orbit (Union of Concerned Scientists (https://www.ucsusa.org/resources/satellite-database/ (accessed on 12 January 2024)). The 90 EO continuing instruments we have described are devoted broadly to environmental research and applications are listed below by their primary instrument type: non-imaging spectrometer, multispectral imagers at various spatial resolutions, VSWIR imaging spectrometers, active systems, operational or semi-operational weather platforms, and/or for other purpose. We provide the launch year and the projected end of life (EOL) year in the tables.

Although the dates for ending missions often change, the published (and expected) termination reveal that 21 of the instruments/satellites in orbit now will be ended over the next three years (2024–2026). The missions of at least twelve are planned to end in 2024 (Sentinel-1A, Sentinels-2A and 2B, METOP B, GOSATs 1 and 2 (nadir and limb), NOAA-15, 18 and 19, PROBA-1, DESIS and HISUI on ISS, and Pléiades 1-A and 1-B). Four more missions str scheduled to end in 2025 including TES nadir, limb on Aura, SMOS, TROPOMI on S-5P, SWARM A, B, C, and Planet’s SkySats. Four more will end in 2026 (Sentinel-3A, OCO-2, SMAP, and ICESat-2).

### 12.1. Satellites Whose Primary Instrument Is a VSWIR Imaging Spectrometer

There are two satellites carrying full-spectrum VSWIR ISs with ≤10 nm bandwidths and ≤30 GSD currently in orbit, the Italian PRISMA and the German EnMAP (Table 13). Additional experimental VSWIR ISs on the International Space Station include the Japanese HISUI, and NASA’s EMIT, plus a VNIR IS, the German DESIS discussed in the next section.

### 12.2. Satellites Whose Primary Instrument Is a Moderate-Spatial-Resolution Multispectral Imager

The LEO satellites carrying MS imagers that are continuing in orbit include two operational programs that survey land, coasts, and inland water surfaces. These are the Operational Land Imager (OLI) and the Thermal Infrared Sensor (TIRS) carried on the NASA/USGS Landsat-8 and Landsat-9 (Table 10); and the ESA/Copernicus Sentinel-2 pairs, each carrying a multispectral imager (MSI, Table 11) on S-2A, and S-2B.

### 12.3. Satellites Whose Primary Instrument Is a Coarse Spatial Resolution Multispectral Imager

These include satellites whose primary instruments are devoted to important environmental monitoring purposes but may also carry multispectral or hyperspectral imagers for scientific studies. The new-generation polar and GEO weather satellites have spatial resolutions that are approaching those in the MODIS era. These are important imaging instruments and include the Copernicus program’s Sentinel-3 A, B pair with the hyperspectral OLCI (Ocean and Land Color Instrument, 21 VNIR bands) and the SLSTR (Sea and Land Surface Temperature Radiometer) a spectroradiometer with nine wide VIS through TIR bands, plus two active TIR fire bands (Table 11). The AVHRR-1 and -2 (before 2007) were the long-term imagers on NOAA satellites 1–14, and AVHRR-3 continues into 2024 on three NOAA satellites (NOAA-15, 18, and 19) (Table 2) and on the EUMETSAT’s METOP B and METOP C. The third is VIIRS (Visible Infrared Imaging Radiometer Suite) which acquires data at moderate spatial resolutions (350–750 m) with 22 VSWIR and TIRS spectral bands, with heritage from MODIS and is carried on three platforms, the NASA Suomi NPP satellite NOAA-20 [JPSS-1] and NOAA -21 [JPSS-2] (Table 10).

### 12.4. Satellites Whose Primary Instrument Is a Non-Imaging Spectrometer

There are three continuing atmospheric chemistry missions with coarse spatial resolutions (≤0.05°) but very high spectral resolution (≤1 nm) covering UV, VIS, NIR and MWIR wavelength bands, the EUMETSAT’s Global Ozone Monitoring Experiment-2 (GOME-2, Table 3) is carried on both METOP B and C. JAXA’s two Greenhouse Gases Observatory Satellites GOSAT and GOSAT-2 (Table 4); and NASA’s Orbiting Carbon Observatory-2 (OCO-2), Table 8. OCO-2 collects near-global data on carbon emissions, atmospheric CO_2_ levels, and estimates of photosynthesis via far-red chlorophyll fluorescence; A fourth instrument is the free-flyer, the Copernicus Sentinel-5 Precursor (S-5P), which has one instrument, the TROPospheric Monitoring Instrument (TROPOMI, Table 8) for atmospheric chemistry, air pollution, and far-red chlorophyll fluorescence, at high spatial resolution (7 km × 7 km). S-5P flies in tandem with the Suomi NPP satellite.

### 12.5. Satellites Whose Primary Instrument Is an Active System

Those in sun-synchronous LEO polar orbits include the NASA’s Soil Moisture Active Passive (SMAP) satellite, but only its L-band passive radiometer is working (Table 7); CryoSat-2 (Table 9) is in a drifting inclined orbit and acquires SAR for ice sheet monitoring and the ESA’s Earth Explorer-4 constellation (Table 9) SWARM A, B, C are in non-polar orbits and flown in specific altitudes and orbit inclinations to maximize the measurements. Each SWARM carries Microwave Radiometers and Vector Field Magnetometers (VFM) to measure the magnetic field. The Canadian satellite CASSIOPE flew as a SWARM-E mission in the SWARM constellation as the fourth satellite until 2021. Lastly, the Copernicus Sentinel-1 series (Table 11), S-1A, a Synthetic Aperture Radar data for surface elevation data that will be joined shortly by and S-1C and S-1D. 

Two others in LEO polar orbits are non-sun-synchronous: NASA’s Ice, Cloud, and Elevation SATellite-2 (ICESat-2, Table 8) carrying the Advanced Topographic Laser Altimeter System (**ATLAS**) for surface elevation and GPS. ESA’s Earth Explorer-2, the Soil Moisture and Ocean Salinity (SMOS) mission (Table 9) with an L-band polarimeter and a passive microwave imager; and Another satellite, the Sentinel-6A Michael Freilich (Table 11), jointly Copernicus and NASA, also flies in an elliptical non-sun-synchronous orbit, but at a higher mid-altitude at 1336 km to measure sea-surface topography with a Synthetic Aperture Radar Altimeter.

### 12.6. Passive Coarse Spatial Resolution Multispectral Observations from GEO

Primarily used for weather and climate but land and ocean imagers are of interest to the Earth Science community to monitor rapidly changing disasters (natural and manmade) and diurnal changes. GOES-16 (East) is stationed over the Atlantic to view eastern North America and South America (Table 16) and GOES-18 (West) is stationed over western North America, South America, and the eastern Pacific (Table 16). These carry the Advanced Baseline Imager (ABI) with 16 VIS through TIR bands, including a red band at 500 m GSD, 3 bands at 1 km, and 12 bands at 2 km. GOES satellites also carry the Geostationary Lightning Mapper **(**GLM). JAXA’s Advanced Himawari Imager (AHI, Table 16) is flown over the western Pacific and Asia on Himawari 8 and Himawari 9. The European Meteosat, Third-Generation (MTG) Imaging-1 (MTG-I1) satellite (Table 16), carries the Flexible Combined Imager (FCI) and Lightning Imager (LI).

### 12.7. Experimental Instruments on the International Space Station

The International Space Station is in an inclined low earth orbit (400 km) from which it observes the most populated portions of the Earth. Six pathfinder instruments (Table 14) are on the ISS for new EO measurements, and three are imaging spectrometers. The DLR’s Earth Sensing Imaging Spectrometer (DESIS) measures the visible and near-infrared from 400 to 970 nm with 2.5 nm high-spectral-resolution bands. Data can be requested from the DLR portal EOWEB. The second imaging spectrometer is the Japanese METI’s Hyperspectral Imager SUIte (HISUI) with two spectrometers, one covering the 400–900 nm range and the other the 895–2481 nm range. Limited archive data are available by request. The third is NASA’ Earth surface Mineral dust source InvesTigation (EMIT), an Earth Venture-4 mission and prototype for the planned Surface Biology and Geology (SBG) VSWIR mission. It has a full-range high-fidelity 400–2500 nm IS with spectral sampling of 7.5 nm and a mission to map surface geologic minerals. The ECOsystem Spaceborne Thermal Radiometer Experiment on the Space Station (ECOSTRESS) is a five-band thermal imager to derive vegetation evapotranspiration and track volcanic activity, urban hot spots, and other thermal events. It is a prototype for the planned NASA SBG TIR mission. The NASA Orbiting Carbon Observatory-3 (OCO-3), a near copy of the OCO-2 free-flyer in polar orbit, is a non-imaging spectrometer with bands that cover the strong and weak CO_2_ absorptions and the O_2_-A band. The sixth experimental sensor is the Global Ecosystem Dynamics Investigation (GEDI), is a full-waveform NIR lidar imager with high-resolution footprints (25 m diameter). In March 2023, it was temporarily moved into storage during another mission’s tenure but is planned to return by fall 2024 and will remain on the ISS until it terminates or the GEDI fails.

### 12.8. High-Spatial-Resolution Commercial Satellites That Are Used for Earth Science

There are five satellite types in polar morning orbits at the start of 2024 (Table 15) with only a few multispectral imaging bands but are widely used for their high spatial resolution (1.2–6.8 m GSD). The CNES Pléiades 1A and 1B are 4-band 2.0 m spatial resolution VNIR imagers. The Pléiades NEO is a constellation of four imagers launched at 90° angles to each other, one pair in 2020 and the second in 2022; they have six VNIR bands and one panchromatic band at 30 cm. The next are three from PlanetScope (now Planet) with short-lived constellations (>200) of Doves, cubesat imagers, which have three or four VNIR bands and 3–4 m GSD. They are launched in groups (“flocks”) with ~3 year life; Super Doves with 8-band VNIR imagers with 3.7 m GSD; and the Planet SkySats, a constellation of 21 small sats whose imagers have 4 VNIR bands, 2 m GSD and a pan band at 0.5 m GSD.

## 13. Starting in 2024, the Next Imaging Spectrometers

### 13.1. The Transition to New Satellites in 2024

The new satellites to be launched in 2024 and beyond will continue ongoing trends. For passive multispectral satellites the primary trends are increasing the numbers of bands, which are generally becoming narrower and targeted for specific EO materials. Multispectral imagers are trending towards hyperspectral imagers, except that their analytical methods remain statistical without having the contiguous bands that allow spectroscopy approaches. A second trend is expansion of the wavelength regions being imaged, with newer passive instruments including some in UV wavelengths and passive thermal instruments that include midwave IR bands and bands at longer Far IR wavelengths, some out to 50 μm and longer. Similarly, SARs are adding multiple frequencies, polarization modes, and interferometry, and extending to longer radar wavelengths, e.g., P-band. In other cases, some instruments include secondary bands (for secondary mission goals) in other wavelength regions, e.g., combining a few VNIR bands with thermal, lidar or microwave/SAR.

We note that most spectral regions not sampled by satellites today are those where most of the incoming solar energy is absorbed by the Earth’s atmosphere, such that few photons reach the land surface and are available to be measured from satellite orbits. There are typically wavelengths with strong atmospheric absorptions, such as water vapor bands. In contrast, there are narrow wavelength features where light does not escape the chemical absorptions in the solar atmosphere. These narrow wavelength regions, termed Fraunhofer lines, are wavelengths that do not contain energy from the sun. Some bands occur in the region of chlorophyll fluorescence at red and far-red wavelengths, e.g., the weak CO_2_ absorption band at 686.72 nm and strong CO_2_ band at 759.37 nm. Photosystem I in higher plants have absorption bands at 675, 695, and 720 (H_2_O absorption) and Photosystem II absorbs at 685 nm, so specific targeted Fraunhofer bands might be useful for studying photosynthetic functioning. ESA’s EE-8, the Fluorescence Explorer (FLEX) mission, will use the full retrieved fluorescence spectrum (640–770 nm) to study photosynthesis, whereas the OCO-2/OCO-3 missions make use of the limited information in the spectrum near 760 nm.

Another theme for EO imagery addresses time and space limitations and conflicts. Data users have two competing and incompatible demands: increased numbers of narrow bands and increased spatial resolution, due to the finite number of photons/unit time that can be measured. The continued emphasis by the user communities on relatively high spatial resolution (e.g., 1–5 m GSD) for EO imagers dictates low LEO satellite orbits that consequently have relatively infrequent repeat observations, because flight swaths become narrower as pixel sizes become smaller. 

One solution for obtaining high-spatial-resolution data and capturing better temporal sampling is to launch multiple copies of the same satellite instrument, offset in time (e.g., Planet’s Doves, SuperDoves and SkySats (Table 15) with 3–8 bands and 2–4 m GSD, and at a global scale, VIIRS flying on three NASA/NOAA platforms, Table 15). This “virtual constellation” approach has been fully demonstrated by the ESA Sentinel satellite series (Table 11) each flying a pair of satellites (S-1, S-2, S-3). NASA and USGS have adopted this model with Landsat-8 and Landsat-9 (Table 10). An alternate method is to coordinate among “compatible” polar-orbiting LEO satellites, e.g., combining data from Landsat-8/9 and Sentinel-2 satellites. NASA has developed a harmonized version to combine these data (Table 12). Combining Landsat and S-2 data significantly increases the likelihood of acquiring cloud-free data to monitor changing conditions, e.g., phenology, crop health and other short-term events.

Imaging Spectroscopy offers new opportunities for obtaining detailed biophysical information about plant species, based on their plant physiological and phenological status and biogeochemistry [54,294,295,296,297]. Spectroscopy is a powerful tool because EO materials can have multiple patterns of absorption features at different wavelengths, identifiable within the same dataset. Thus, ISs enable identification of geologic minerals [298,299], soil types and their composition [300,301] and snow and ice conditions [302,303,304] as well as the existing state of health and conditions of these materials including weathering [305,306,307], ocean and coastal margins [308,309,310], evidence for environmental pollutants [311,312], and for recording and tracking impacts of disasters [313,314]. 

Imaging spectrometers, at least when the full spectrum is the basis of analysis, rely on principles of physics that underly spectroscopy, which can advance ecosystem science by identifying plant functions, photosynthetic processes, and energy transfers between the land and atmosphere. For plants, measurements of traits can include concentrations of foliar pigments (e.g., chlorophyll/m^2^), leaf mass per area (dry m/area), some nutrients, e.g., nitrogen/leaf area (N/m^2^) [315,316]. The shape of the spectra can be used to identify specific minerals [317], soils [318], or plant species [319,320,321,322,323]. Spectra provide important clues for hydrology [302,303,304,324], plant physiology [50,325,326,327], surface biogeochemistry [52,328,329,330,331], ocean and freshwater biology and coastal margins [308,309,332], as well as the type of pollution [312,333,334,335], and type and extent of disasters [311,313,336,337].

### 13.2. The Plankton, Aerosol, Cloud, Ocean, Ecosystem (PACE)

Oceans comprise over 70% of the Earth’s surface compared to 30% for land and ice surface areas. NASA’s Plankton, Aerosol, Cloud, Ocean, Ecosystem (PACE) satellite was successfully launched via SpaceX in Feb. 2024. PACE overlaps with the last mission phase for Aqua, and extends capabilities for monitoring marine and coastal regions, and large freshwater lakes, globally (Table 17). PACE’s primary instrument is the Ocean Color Imager (OCI), a passive UV/VNIR (350–885 nm) imaging spectrometer with narrow (5 nm) spectral bands, plus several SWIR bands (Table 17), designed for the low reflectance of ocean waters and the ability to identify phyla of algae and plankton and optical constituents of seawater at a coarse spatial resolution (1 km^2^). PACE has two additional passive instruments (https://pace.oceansciences.org/ (accessed on 1 May 2024).

The polarimeters on PACE include the Spectro-Polarimeter for Planetary Exploration (SPEXone), a passive UV-NIR polarimeter with 5 view angles that measure the intensity and polarization of light reflected from the surface to accurately characterize aerosols. The Hyper-Angular Rainbow Polarimeter-2 (HARP2) is a wide angle polarimeter measuring microphysical properties of aerosols and clouds and properties of land and water surfaces with four VNIR bands and <60 view angles. It has heritage from the Hyper-Angular Rainbow Polarimeter (HARP), a NASA cubesat deployed on the ISS in 2020.

PACE’s data will primarily address ocean states and processes, including CO_2_ exchange between the ocean and the atmosphere. PACE addresses concerns about the future of the ocean carbon sink related to the abundance and activity of different species and phyla of plankton, due to the gradual acidification of the oceans. Because the total reflectance from ocean waters is especially low, only approximately 2–5% of the total incoming irradiance, large pixels are needed to collect a strong photon signal to extract a stable measurement of relevant properties [338]. In the open marine environment, unlike terrestrial sites, there is far less spatial variation across short distances, which is complex on land due to history and topography. Oceans have other complexity issues due to tides, currents, surface winds, and daily/seasonal changes in light and temperature patterns, all of which contribute to changing marine algae and phytoplankton species and phyla produce spectral differences that can be mapped by their photosynthetic pigments. 

PACE data will also be used to examine the influence of precipitated atmospheric aerosols on phytoplankton growth in the surface waters and will identify the extent and duration of harmful algal blooms. The complex physical, chemical, and biological processes in oceans and coastal waters have both positive and negative impacts on carbon fluxes and biogeochemical cycles. Greater understanding of the complexity of marine interactions and the physiological processes of phytoplankton and zooplankton [339,340] can contribute to improved oceanographic models and greater understanding the functioning of the entire ocean ecosphere. 

### 13.3. The Carbon Mapper

There is an urgent need to accelerate reductions in emissions of methane (CH_4_) and carbon dioxide (CO_2_) to meet climate goals (https://carbonmapper.org/ (accessed on 1 May 2024)), https://carbonmapper.org’carbon_mapper.html/ (accessed on 1 May 2024). However, developing effective policies to mitigate trace gas emissions requires an effective method to monitor the Earth and accurately account for time and location of emissions. The Carbon Mapper is a new two-satellite constellation designed to map methane, carbon dioxide and water vapor using a full-spectrum IS instrument. The Carbon Mapper program plans to launch the first and second Tanager satellites in this series in mid-2024 (Table 17). The Carbon Mapper Program is a public–private non-profit with partners in business, industry, government organizations and universities that include Planet, the Jet Propulsion Laboratory, University of Arizona, Arizona State University (ASU), California Air Resources Board (CARB), the High Tide Foundation, the Rocky Mountain Institute (RMI), and Bloomberg Philanthropies (https://carbonmapper.org/our-mission/technology/ (accessed on 1 May 2024)). Its mission is to use imaging spectrometry to locate, quantify and track CH_4_ and CO_2_ point-source emissions from space. The plans include a two-satellite IS constellation in Phase One to demonstrate the value and methodology of these instrument, which will be followed by a Phase Two buildout of a full-spectrum IS Tanager constellation (appropriately named after a beautiful bird of many colors) to globally map these compounds at high-frequency intervals. The current design is built on updated Next-Generation StarSat platforms with an IS from the Jet Propulsion Laboratory, built first on the PRISM model and later EMIT (Table 14) configuration, but will have a much higher SNR than either the airborne AVIRIS-NG or the Global Airborne Observatory (GAO) from ASU. Airborne pilot projects conducted over the past decade have demonstrated the potential for an operational satellite data service to locate sources of carbon emissions, especially mega-emitters [144,253,337,341,342,343,344].

### 13.4. The MethaneSat Satellite

While Carbon Mapper is a hybrid public–private mission (Table 17) with contributions from industry (Planet), government (the Jet Propulsion Lab, California Air Resources Board), multiple universities, and philanthropy, MethaneSat is purely a non-profit endeavor and is a subsidiary of the Environmental Defense Fund with funding from philanthropic organizations and is being flown in partnership with the New Zealand Space Agency (https://www.methanesat.org/ (accessed on 1 May 2024)). The program is led by Dr. Steven Hamberg from the Environmental Defense Fund. MethaneSat was launched in March 2024, and will provide high spectral and spatial resolution at regional, not global scales, but is capable of monitoring small emitters. 

MethaneSat [345,346] has two passive infrared Littrow spectrometers measuring two regions (1249–1305 nm and 1598–1683 nm), the former for O_2_ measurement with 0.20 nm spectral resolution (0.006 nm spectral sampling), and the latter for measurements of CO_2_ and CH_4_ with 0.25 nm spectral resolution (and 0.008 nm spectral sampling). It will fly at 535 km and have a 200 km swath, yielding spatial resolution of 100 m across track by 400 m along track. It is designed to collect data in approximately 50 regions known for oil and gas production. With MethaneSat combined with the Carbon Mapper, 80% of regions where oil and gas are produced globally can be viewed and monitored. To support the satellite mission and validate the quantitative measurements of methane concentrations, the MethaneSat mission will also underfly their study sites with a Lear jet carrying similar instruments for validation. 

### 13.5. Thermal Infrared Imaging Satellite for High-Resolution Natural Resource Assessment (TRISHNA)

TRISHNA is a joint project of the French CNES and Indian ISRO space agencies (https://www.eoportal.org/satellite-missions/trishna#eop-quick-facts-section/ (accessed on 23 November 2023)). It is a multiband high-spatial-resolution thermal infrared imager, now delayed with a planned launch in 2026 (Table 17) into a sun-synchronous descending orbit with an Equatorial crossing time between 12:30 and 13:30 (https://database.eohandbook.com/database/missionsummary.aspx?missionID=866/ (accessed on 16 March 2024)). Following the recommendations set forth by Global Climate Observing System (GCOS) program recommendations (https://gcos.wmo.int/en/essential-climate-variables/land-temperature/ecv-requirements/ (accessed on 16 March 2024)), TRISHNA will have eight bands measuring at 50 m GSD: there will be four VNIR bands to measure vegetation type and areal cover per land cover type; two SWIR bands (at 1.38 μm and 1.65 μm) to measure cloud type and amount; and four TIR bands to measure temperatures of land surfaces, cloud tops, and fires. Over land, TRISHNA will also measure surface albedo and reflectance, including snow and ice cover. 

The mission has six objectives: (1) to monitor ecosystem stresses and water use, specifically monitoring the surface energy and water budgets of continental biospheres; (2) coastal and continental waters; (3) urban microclimates and heat islands; (4) geology; (5) the cryosphere; and (6) the atmosphere. To be launched in 2024, TRISHNA fills an important TIR data gap between 2024 and the 2027/2028 period, a consequence of postponing the Copernicus LSTM mission (see below) which is now timed to fly in concert with the NASA SBG-TIR mission (2027). It will likely be near its end of life by the time the LSTM is launched.

### 13.6. NASA-Indian Space Research Organization SAR (NISAR)

NASA and the Indian Space Agency (ISRO) are preparing a “first-of-its-kind” dual-frequency radar mission, NISAR (NASA-Indian Space Research Organization SAR, Table 17). Launch is now planned for later in 2024 with an S-band SAR (9 cm) from ISRO and an L-band SAR (24 cm) from NASA [347]. It will have a swath of 240 km reaching 82° N to 78° S, due to its left-looking observation orientation. NISAR will have a 12 day repeat frequency; measured on daily ascending and descending passes (06:00 and 18:00), the actual passing frequency will be every 6 days, with expected coverage of 4–6 times monthly [348]. Ground spatial resolution is between 3 and 10 m, depending on the collection mode. The L-band and S-band SARs will have routine single or dual polarization with the L-band observing targeted quad-pol sites in the USA and India and the S-band will provide quasi-quad pol over select targets. The satellite has a 12 m diameter mesh reflector antenna mounted on a 9 m boom oriented away from the satellite.

NISAR’s mission includes monitoring Earth surfaces for crust movement from slips, slides, and volcanic eruptions. It will be able to track hazards like surface deformation (before catastrophes) and after, including earthquakes, landslides, debris flows, subsidence, and sinkholes (https://nisar.jpl.nasa.gov/mission/mission-concept/ (accessed on 12 May 2024)). A second foci is monitoring water and cryosphere resources and changes, e.g., ice movement and loss (or gain), with a focus on glaciers and ice sheet changes, and consequences of changes in the sea level in coastal areas. The intensification of climate has brought more frequent forms of extreme weather in devastating floods, drought, hurricanes, tornadoes, and its impacts on wildfires and even insect infestations. NISAR will monitor changes in surface water distributions from lakes and rivers, and it will monitor evapotranspiration and irrigation patterns, and groundwater use and recharge. NISAR will provide information on the distribution of vegetation and biomass to better understand changes in the earth’s ecosystems and its responses to climate-related disasters. Land-use changes are intensifying, and these forces are also responsible for global impacts on ecosystems, changes in ecosystem composition and for losses of biodiversity. How human-caused changes enhance or conflict with weather and climate-related disasters is another focus of research on data from this instrument.

NISAR will produce approximately 140 PB data over its three-year mission, approximately 85 TB of data daily [349]. For comparison the entire Earth Science Data and Information System (EODIS) in 2017 was 22 PB. The NISAR data volume is pushing the limits of both efficient storage and delivery of data as file sizes are large and data volume is even larger. NASA is moving data storage from the NASA DAAC to the commercial cloud (https://www.earthdata.nasa.gov/learn/articles/getting-ready-for-nisar/ (accessed on 12 May 2024)).

## 14. Second-Generation EO Polar Weather Satellites: METOP SG-1A and SG-1B

These global-scale “coarse” resolution satellites, now with 375 m pixels compared to older weather satellites (750 m pixels), are therefore of greater interest to environmental scientists. These global monitoring satellites serve a wide range of applications in addition to weather and climate research: understanding large-scale land cover changes like regional droughts, wildfires, and many other applications such as urban expansion, forest die off, and increasing or decreasing agricultural land. They continue the current generation of EO global mapping satellites that obtain global coverage every 1–3 days, with instruments with multiple spectral bands in the solar spectrum, often including several TIR bands.

The EUMETSAT Polar System-Second Generation (EPS-SG) with expected launches in 2025 for 1A and 2026 for 1B will be composed of a series of three instruments for each of the two platforms. METOP-SG 1A and METOP -SG 1B. These two will be flying together in the midmorning orbit for operational weather satellites (Table 18) and will carry 10 complementary payloads, SG-1A for land observations, including the Sentinel-5 and it will carry the Infrared Atmospheric Sounding Interferometer-NG (IASI-NG), Microwave Sounder (MWS), METImage, the Multiviewing Multichannel Multipolarization Imager (3MI), and the Radio Occultation (RO) sounder. The METOP-SG-1B for oceanography and climatology will carry the Ice Cloud Imager (ICI), MicroWave Imager (MWI), SCAtteromter (SCA), Radio Occultation (RO) sounder, and the Advanced Data Collection System Argos-4. The 3MI will measure VIS and NIR channels in 520 × 520 CCD arrays from 14 directions across a 2200 km swath. The SWIR channels will have a 520 × 256 CCD array viewed from 12 directions. METImage, replacing the AVHRR-3 on the METOP -B, -C, is a multipurpose imager with capability for aerosols and cloud microphysics. IASI-NG with a swath of 2000 km, is a temperature and humidity sounder interferometer measuring ozone profiles and total column profiles of greenhouse gases (C_2_H_2_, C_2_H_4_, C_2_H6, CFC-11, CFC-12, CH_3_OH, CH_4_, CO, H_2_CO_2_, HCN, HNO_2_, HNO_3_, N_2_O, NH_3_, PAN, SO_2_). The MWS is a temperature and humidity sounder for all weather conditions, including precipitation. It replaces the AMSU-A and HMH on METOP A-C. The Radio Occultation Sounder measures the phase delay in GPS signal (GPS, Galileo, BeiDou) from ionospheric electron profiles along path. SG-1A replaces the GRAS flown on METOP A-C. SG-1A will provide improved numerical weather prediction, nowcasting weather at high latitudes (where geostationary data are usually not available), climate monitoring, ocean surface wind vectors, sea-surface temperatures, sea ice, and other marine products, and with some instruments of significant interest to environmental scientists and other environmental applications. Imagery (METImage, 3MI and Sentinel-5) collected by SG-1A includes the UV/VIS to the TIR for atmospheric chemistry and on SG-1B imagery for oceanography and climatology. Both platforms carry high-resolution sounders.

All METOP-SG satellites have an expected life of 7.5 years and fly in a sun-synchronous polar LEO at an average altitude of 817 km. This series will fly in descending nodes with an equatorial crossing time crossing of 09:30 mean local solar time, and a repeat cycle of 29 days.

### 14.1. METOP SG-1A

The first two satellites in the METOP-SG-1 series will carry the Sentinel-5A, to be launched in 2025 and Sentinel-5B, in 2032 with 7.5-year life-time (Table 18).

The most widely used instruments for EO studies and monitoring on this platform will be its multispectral imagers and thermal IR sensors (Table 18). Large-scale land surface monitoring will be achieved through the METeteorological Imager (METImage), previously called Visible and Infrared Imager, a passive multispectral radiometer measuring 20 bands from 443 nm (blue visible) to 13.345 μm (TIR) (Table 18) with heritage from AVHRR, VIIRS, POLDAR and PARASOL. It will provide high quality data for regional and global numerical weather prediction, nowcasting, and climate monitoring. It will measure high horizontal cloud products including data for microphysics analysis, sea-surface temperatures, aerosol products, vegetation, snow, fire monitoring products, and polar atmospheric motion vectors. 

The Multiviewing Multichannel Multipolarization (3MI) instrument has heritage from POLDER and provides high quality aerosol imagery for climate records, air quality, surface albedo, cloud microphysics, cloud height and optical depth characterization and data on ocean color. The 3MI is a passive 12 band VSWIR radiometer, nine of which are polarized reflected radiances at three angles (−60°, 0°, +60°) and 14 different viewing geometries. Its primary mission is to improve air quality information, providing high quality imagery of aerosols, e.g., aerosol optical depth, particle size (PM2.5, PM10), refractive index, height, and absorption.

The passive Micro-Wave Sounder (MWS) makes temperature and humidity soundings under nearly all-weather conditions, including precipitation (Table 15). It is the replacement for the older AMSU-A on METOP A, B, and C (Table 2). The MWS has 24 channels with spatial resolutions of 17 km for channels 89–229 GHz, 20 km for channels 50–58 GHz, and 40 km for channels at 23.8 and 31.4 GHz. The MWS has single polarization (H or V, with channel 6 being only H polarized). 

The Radio Occultation Sounder (RO) is the last of the three sounders on SG-1A satellites. It measures temperature and humidity soundings with high vertical resolution of 300 km horizontal and 0.5 km vertical resolution. It measures a phase delay due to refraction by water vapor between the satellite and the GPS and GLONASS satellites, with an average spacing of 680 km (providing global coverage with 300 km spacing) in five days.

### 14.2. MetOp-SG-1B

SIG-1B hosts Ice Cloud Imager (ICI), which is a new instrument measuring cloud ice from differential signatures in 13 water bands with 11 mm and sub mm frequencies at 183, 325.15, 448 GHz, and individual frequencies of 243.2 + 2.5 and 664 + 4.2 GHz. The MicroWave Imager (MWI) is a multi-purpose sounder for precipitation, temperature, and humidity with a conical 53.1° zenith angle scanner (Table 18). The MWI measures 18 frequencies between 18.7 and 183.31 GHz over 26 channels. The SCA (SCAtterometer), replacing ASCAT on MetOp A-C, provides sea-surface wind vectors and large-scale soil moisture. The SCA is a C-band 5.355 GHz side looking (left and right) scatterometer with a 660 km swath separated by 525 km gaps along track. The SCA measures three looks for each pixel at 45°, 90°, 135°azimuth. Additional instruments are onboard for measuring space weather. The MicroWave Imager (MWI) is a multipurpose sounder for precipitation, temperature, and humidity with a conical 53.1° zenith angle scanner.

### 14.3. Sentintel-5

Sentinel-5’s mission is to measure atmospheric concentrations of trace gases, providing improved air quality assessments, atmospheric gas composition and climate interactions (https://sentinel.esa.int/web/sentinel/missions/sentinel-5#:~:text=The%20Sentinel%2D5%20mission%20consists,3%20(2305%2D2385nm/ (accessed on 8 December 2023)). It will operate in the mesoscale push-broom mode, making nadir observations across a wide 108° field-of-view (FoV), covering a 2670 km swath to provide nearly daily global coverage.

The S-5s carry a high-resolution imaging spectrometer measuring from the UV (in five hyperspectral segments) and two hyperspectral segments in the SWIR. The Sentinel-5 TROPOMI instrument builds upon the still-orbiting S-5P precursor mission (Table 18).

Sentinel-5 will measure trace gases, that when combined with data from the 3MI, METImage, and the IASI-NG sensors on SG-1A, will improve air quality forecasts. The main data products retrieved are NO_2_, SO_2_, HCHO, CHOCHO and aerosol concentrations. Additionally, Sentinel-5 will deliver quality parameters for CO, CH_4_, and stratospheric O_3_ with daily global coverage, to be used for climate, air quality, and ozone/surface UV applications. Sentinel-5 flights are coordinated with the Sentinel-4A, which will be flown on the Geostationary Meteosat Third-Generation (MTG) sounder (MTG-S1), which is expected to launch in September 2025, with an expected 8.5-year life. Sentinel-4B is scheduled for launch on MTG-S2 in 2035 for a 10-year mission.

## 15. New ESA Earth Explorer Satellites Expected between 2024 and 2026

Three ESA Earth Explorers will be launched in this period, EarthCARE (Earth EE-6, 2024), Biomass (EE-7, 2024), the Fluorescence Explorer, FLEX (EE-8, 2025) and a new addition, Harmony (EE-10), all are planned for launch between 2024 and 2029. 

### 15.1. ESA’s 6th Earth Explorer Mission: Earth Clouds, Aerosols and Radiation (EarthCARE)

The EarthCARE mission is the 6th mission sponsored by ESA’s Earth Explorer program (Table 19) and is a joint mission between ESA, JAXA (Japanese Aerospace Exploration Agency) and the Japanese National Institute for Communications and Technology (NICT). This satellite will carry both lidar and radar instruments. It is expected to be launched in 2024 (https://www.esa.int/Applications/Observing_the_Earth/FutureEO/EarthCARE/ (accessed on 22 February 2024)) into a sun-synchronous LEO at 394 km altitude, with a repeat frequency of 25 days. Its mission is to measure and monitor clouds, aerosols, and the Earth’s reflected radiation. EarthCARE’s goal is to improve medium range weather forecasts through better understanding of the interactions between clouds and aerosols and how these properties affect reflected energy into space [349]. Through these measurements, it plans to develop a 4D (spatial and temporal) structure of the radiative flux field from the top of the atmosphere to the Earth’s surface.

EarthCARE will acquire measurements from a suite of four instruments: an ATmospheric LIDar (ATLID) measuring at 354.8 nm for vertical profiles of clouds and physical parameters of aerosols and thin clouds (altitude, optical depth, backscatter ratio, depolarization ratio); a Doppler Cloud Profiling Radar (CPR) at 94.05 GHz to measure vertical profiles of clouds and the velocities of cloud particles; a **m**ulti**s**pectral **i**mager (MSI) with seven spectral channels (VSWIR-TIR) providing cross-track (15-km swath) observations of clouds and aerosols at 500 m pixel resolution; and a Broad-Band Radiometer (BBR) measuring top-of-atmosphere radiances and fluxes in two channels, with three fixed view directions (nadir, +50° fore and −50° aft) and covering wavelengths from the UV to the SWIR and into the far infrared (15–50 μm). While not directly aimed at supporting environmental research of the Earth’s seas or land masses, this mission will investigate which data are useful for interpreting the forcing factors that are acting on global ecosystems, creating constant changes.

### 15.2. ESA’s 7th Earth Explorer Mission: Biomass

The Biomass instrument is a P-band Interferometric Synthetic Aperture Radar (SAR) (https://earth.esa.int/eogateway/missions/biomass/ (accessed on 22 February 2024)). Biomass is the seventh in the ESA Earth Explorer Program (Table 19). It is expected to be launched in 2024 and will be the first P-band radar in space [350]. It is dedicated to quantifying the amount and global distribution of aboveground forest biomass and carbon stored in forests (generally estimated to be at approximately half of the biomass value). Because of the long wavelength of P-band radar, at 435 MHz approximately 70 cm, it will have better penetration deep into the canopy of tall forests and to the ground layer, thus it will have greatly improved coverage of the lower canopies and ground vegetation than observed with shorter-wavelength radar satellites. This will reduce uncertainty in estimating carbon stocks and fluxes associated with the terrestrial biosphere and increase understanding of the vertical and horizontal structural patterns in biomass.

**Figure 24:**
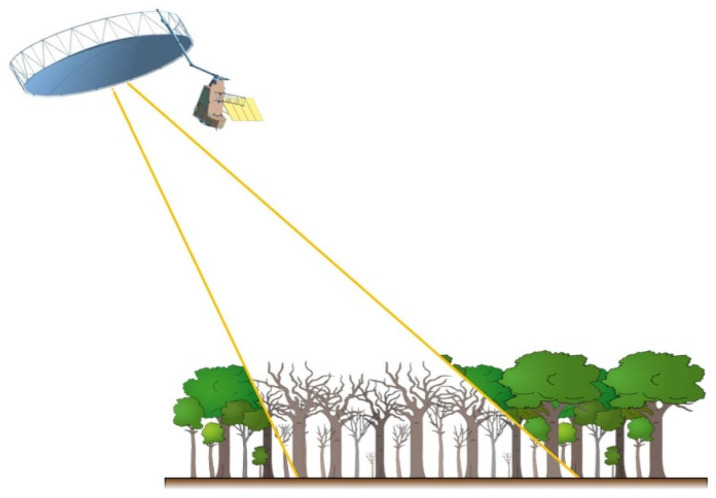
This schematic diagram illustrates the penetration of long-wavelength P-band imagers for measuring the full forest height and structure down to the ground surface. It is expected that Biomass, the first satellite with this SAR technology, will produce more detailed and accurate estimates of total biomass and changes over time than previous missions (figure from ESA showing P-band radar piercing through forest canopy pillars.jpg).

The P-band radar instrument will pioneer the use of a quad polarized (both sending and receiving vertically and horizontally polarized radar beams), for vertical (VV), horizontal (HH), and cross polarized (VH and HV) acquisitions. While VH and HV data are generally similar, their differences illuminate subtle structural patterns. One of the technological challenges of the design of the Biomass platform is that the mission has conquered the challenge of how to transport the large (12 m diameter) umbrella-like antenna into space and unpacked for use. The solution is a wire–mesh antenna that will unfold in space and capture the backscattered signal from the vegetation, and from the top of the canopy to the ground, as illustrated (Figure 24). The stripmap mode of data collection has three potential swaths based on rolling the satellite. The maximum swath is 153 km. The highest spatial resolution is 50 m and will provide more information on the vertical structure and biomass distribution of global forests than is currently available. Furthermore, more accurate estimates of growth and harvest patterns will be obtained with remeasurements (https://database.eohandbook.com/database/missionsummary.aspx?missionID=768/ (accessed on 10 May 2024)). The high-spatial-resolution pixels have application for disturbance mapping, to be determined every six months. remeasurements.

### 15.3. ESA’s 8th Earth Explorer Mission: FLuorescence EXplorer (FLEX)

FLEX is the first EO mission that will address plant physiology from space, and at an ecologically meaningful scale (e.g., ≤300 m), and is specifically designed to study the biochemistry underlying the carbon cycle within ecosystems, with implications for the Earth System (Table 19). From a LEO polar orbit, FLEX will derive steady-state solar-induced chlorophyll fluorescence (SIF) and related plant physiology variables from terrestrial vegetation. Its free-flyer carrying the FLuORorescence Imaging Spectrometer (FLORIS), a push-broom hyperspectral imaging instrument, will fly in tandem with Sentinel-3D (synergistic with OLCI and SLSTR), with a planned launch in 2025 for a 3.5-year lifetime [351] (Table 19). The swath for the FLEX free-flyer is 150 km wide with a ground spatial resolution of 300 m. It will have a 10:00 equatorial crossing time, optimizing the signal linking fluorescence with photosynthetic efficiency. The FLEX satellite is being built by Thales Alenia Space (France). It is a small, 2.16 m^3^ platform weighing ~460 kg (fully loaded with 30 kg propellant), with a mechanical configuration based on Myriade evolution and Copernicus Sentinel-3 avionics. There are two solar array panels on each side of the main body, extending the total width to 5.2 m when deployed in orbit.

Fluorescence is a biproduct of photosynthesis that occurs when plants absorb more solar energy than can be used, it will release some energy through dissipation process to protect the photosystems [352,353,354]. SIF is a flux of emitted energy produced at longer wavelengths than the absorbing wavelengths, which results in a unique and characteristic emittance spectrum across the VNIR wavelengths (~640–780 nm), with maxima (aka “peaks”) at ~685 nm and ~740 nm. Accurate determination of SIF requires very narrow bands (<0.3 nm) around these specific VNIR wavelength features. The derived total SIF value can be converted to an indicator of photosynthetic activity and vegetation health (https://earth.esa.int/eogateway/missions/flex/ (accessed on 3 May 2024)).

Accurate information about photosynthetic performance on the scale of ecosystems is essential to portray and predict carbon exchange due to biogeochemical cycles associated with plant growth and phenology, which affect carbon, water, and nitrogen cycles in Earth’s global system. These are also linked to cycles of nutrients like sulfur, phosphorous, and other trace minerals that are essential for plant growth. Furthermore, the ability to accurately estimate plant photosynthetic production across landscapes as Gross Primary Production (GPP) is a major goal of regional and global monitoring from space [352,353,355,356]. While GPP has been estimated by various satellites throughout much of the EO satellite era, the challenge today is to “close the carbon budget”, which requires more rigor than has been available previously. Many of the processes affecting photosynthesis are non-linear and depend on physical environmental conditions (temperatures, humidity, net radiation, and others) and vary according to the physiological and phenological states of the vegetation. Estimates of carbon sequestration in global models currently do not account for SIF. If SIF can be measured at useful local (ecosystem) scales, it would provide a more direct way to estimate GPP and provide essential data that will help unravel photochemistry on landscape scales.

Only recently has satellite technology improved sufficiently to measure the high spectral resolutions with imagery at band resolutions of 0.1–0.7 nm necessary for detection of fluorescence signals [357]. SIF energy is continuously being passively emitted during daylight periods, especially at high solar inputs during midday (a fluorescence glow ranges from about 0.5% to 2% of the total energy absorbed from photosynthesis). Increased fluorescence, especially sustained over time, can be tied to sources of plant stress, e.g., overall health. FLEX will be the first satellite capable of directly measuring the small amount of fluorescence emitted by plant chloroplasts from space [358]. 

The FLEX mission’s measurements made by FLORIS of reflected and emitted radiation in the 500–780 nm spectral range, will acquire very high (0.1 nm) spectral sampling to attain 0.3 nm resolution in two wavelength regions associated with the O_2_-B and O_2_-A atmospheric oxygen absorption bands (677–686 nm and 759–769 nm) (https://earth.esa.int/eogateway/news/flex-infographic/ (accessed on 2 may 2024)). A second spectrometer with lower 2 nm spectral resolution (0.5 nm sampling) will measure/derive additional atmospheric and vegetation parameters which include chlorophyll; it will also measure reflectance in the green bands (500–600 nm) needed to compute the Photochemical Reflectance Index, PRI [359], which describes another competing biological energy dissipation pathway, e.g., [360]. However, there is another reversible biochemical process occurring in sync with SIF, the light activation of special carotenoid pigments, the xanthophylls, which provide a second mechanism for protective energy dissipation as heat, linked to a commensurate downregulation of photosynthesis. Accurate description of SIF thus requires accounting for this alternate photochemical quenching process, captured via the PRI vegetation index (https://earth.esa.int/eogateway/missions/flex#instruments-section/ (accessed on 2 May 2024)).

FLEX will orbit in tandem with a Copernicus Sentinel-3 satellite to make optimal use of S-3’s relevant, contemporaneous space observations (De Grave et al., 2020). FLEX will fly 100 km ahead (within 6–15 s) of Sentinel-3D from a sun-synchronous LEO orbit at a higher altitude (814 km) than S-3 and have a 27 day repeat cycle. The FLORIS swath falls within the nadir OLCI 1270 km swath and the nadir SLSTR 1400 km swath, as well as the SLSTR backward look, all made at S-3’s 740 km orbit height. This pairing provides the atmospheric correction and vegetation biophysical parameters collected by the S-3 OLCI and SLSTR imagers necessary for processing FLORIS spectral data (https://earth.esa.int/eogateway/missions/flex/description/ (accessed on 2 May 2024)). When FLEX is in orbit with a 10:00 mid-morning equatorial crossing time, it will periodically fly above Landsat’s 8 and -9 and the Sentinel-2 series satellites.

### 15.4. Earth Explorer-10 Harmony

The EE#10 is Harmony (formerly called Steroid), to be launched in 2029 for a 5-year mission. The Harmony EE-10 mission is currently in Phase-A feasibility studies [361]. The measurements will monitor glacier and sea ice motion, crustal motion, and magnetic and geodynamic measurements. The mission concept entails two satellites that would fly in tandem with (or flank) a Copernicus Sentinel-1 radar satellite in the same sun-synchronous orbit: the S-1 flies at 693 km with a 98.2° inclination angle and a twelve day repeat cycle [362]. Each Harmony satellite would carry a “receive-only” passive Synthetic Aperture Radar (SAR) as its main instrument, and would operate in two different formations, stereo and close flight. Each Harmony satellite would also carry a multibeam thermal-infrared instrument, used to determine height-resolved cloud movements. In the absence of clouds, this TIR instrument would measure sea-surface temperature differences. Harmony will be the first mission to provide data to study interactions between the air and the ocean surface by simultaneously measuring wind, waves, currents, sea-surface thermal differences, and cloud movements. It is expected that Harmony will expand understanding about the marine atmospheric boundary layer. It is at the formation stage so instrument and flight details are not yet available.

## 16. Recommended Science Priorities to NASA in the 2017 Decadal Survey

The U.S. National Academies of Sciences, Engineering, and Medicine (NASEM) undertake programmatic reviews of the space programs at decadal intervals, at the request from NASA. The 2017 Earth Sciences Decadal Survey (DS) is only the second review for NSA’s Earth Science Directorate. The Atmospherics, Planetary, and Heliophysics programs have had a long history with this NASA program and taken advantage of the directions the NASEM provides. While NASA requests the study, NOAA and USGS are included as collaborative agencies that manage satellites and provide satellite data. The NASEM’s recommendations to NASA are based on thousands of hours of time donated by the scientific community in the USA and abroad, including hundreds of hours by specialists that provide information to the committees based on their expertise and knowledge. This includes technical information on how design differences were resolved for the different instruments, the tests performed to evaluate performance, and other aspects, including problems solved and any remaining problems. The reports are reviewed by many scientists in the community who are independent of the committees. Thus, the DS represents a broad segment of the scientific communities’ understanding where critical gaps and uncertainties in Earth Science need attention and where there is sufficiently high benefit to society, makes recommendations to NASA.

In 2017, the DS recommended five “Designated Observables” (DOs) viewed as meeting the highest and most immediate satellite needs of the nation across the breadth of Earth Science disciplines [107]. The DS also challenged NASA by calling these instruments an “Earth System Observatory”, with an emphasis on developing the DOs in an intentional, coordinated effort to obtain the benefit of synergistic operations and data. In addition to meeting science goals, the DS chose these five instruments as representing a balanced investment across the diverse interests of Earth Sciences. None of the recommended DOs are currently in orbit although the Surface Biology and Geology Thermal Imager (SGB-TIR) is closest to launch and will likely fly in 2028, near the end of the DS decade. That mission has been greatly helped by the Italian Space Agency (ASI) partnering with NASA on this instrument.

The original recommended SBG mission now consists of two separate instruments and platforms to optimize their orbits and flight altitudes as SBG-TIR and SBG-VSWIR missions. two instruments, the SBG-VSWIR is a global mapping imaging spectrometer and the SBG-TIR is a multiband thermal imager. The SBG-VSWIR, arguably the most “shelf ready” of all the DOs in 2017 (attributable to its having been in the second tier of those recommended in the first Earth Decadal Survey in 2007) [270]. On page 129 of the 2017 DS it was recommended to be the first of the DOs flown and on page 135 it states that SBG “is linked to one or more Most important or Very important science objectives from each of the five panels and feeds into the three DS integrating themes: water and energy cycle, carbon cycle and extreme events”. Nonetheless, it has been delayed to be the last of the five recommended DOs (possibly to 2032), by internal decisions at NASA. Meanwhile, the surface deformation and change (SDC) mission to monitor changes in the 3D structure of the Earth’s surface, from earthquakes and volcanoes and loss of forests to logging and wildfires has been held in planning throughout the decade, mostly to allow time for the NISAR and ROSE-L missions to fly first and obtain lessons learned. The last two missions, mass change (MC), intended to provide continuity for the GRACE-FO with some upgrades. It is now part of a constellation of satellites called MAGIC, a system with two platforms, each with two pairs of MC instruments to measure gravitational anomalies. The last two instruments were combined into a program called Aerosols, Clouds, Convection and Precipitation (ACCP) have been greatly expanded in scope beyond the original recommendations provided by the Decadal Survey, resulting in considerable cost increases. They are now scheduled for flights in 2029 and 2030.

### 16.1. Surface Biology and Geology (SBG) Mission

The SBG-VSWIR and SBG-TIR instruments together will measure the temperature, composition and properties of the land, water, and oceans part of NASA’s Earth System Observatory (ESO) that includes all missions recommended by the 2017 NASEM Decadal Survey. NASA plans to launch the Surface Biology and Geology (SBG) mission satellites (the name will change on launch), between 2028 and 2032 (https://sbg.jpl.nasa.gov/ (accessed on 15 May 2024)). This two-instrument mission called for a full-spectrum VSWIR imaging spectrometer paired with a thermal imager [107] but the instruments were split into different orbits and crossing times after the architecture review due to incompatible differences in mission requirements by the stakeholders.

#### 16.1.1. Surface Biology and Geology: SBG-VSWIR

The SBG imaging spectrometer is a multipurpose imager for natural vegetation, agriculture, and aquatic (near shore) systems [363]. The design will provide measurements about the composition and functioning of terrestrial ecosystems [364,365]. Among the many applications the VSWIR can provide, the most critical is its capability to provide information about ecosystem biodiversity, which is seen as essential to develop a global baseline estimate/survey of biodiversity [297,366] and for future monitoring of biosphere changes. 

The SBG-VSWIR has 10 nm or better spectral resolution across the spectral range of 380–2500 nm (Table 20), with relatively high (7.5 nm) spectral sampling [367], an improvement over existing NASA imagers. This provides continuous VSWIR spectra capable of describing the composition and deriving the functioning of terrestrial and aquatic ecosystems [364,365]. The SBG-VSWIR will have a spectral range of 380–2500 nm, with high spectral fidelity and spectral uniformity (no smile or spectral shift observed across the swath of the detector, or keystone, a spatial distortion across the swath due to expansion of the area measured at the limbs). The planned equatorial crossing time is late morning (11:00), an hour later than most Landsats (10:00 am). 

The concept for this mission has heritage from the two NASEM Decadal Surveys of 2007 and 2017. The single VSWIR instrument will fly in the Landsat-8/9 orbit, now perhaps in 2032, four or five years after the SBG-TIR flies in its early afternoon orbit. Its design and capabilities are based on the Dyson design of the EMIT instrument, currently on the ISS [45,256] and conceptually like HyspIRI (the previous 2007 DS recommendation). In addition to a decade of planning for the HyspIRI mission, the VSWIR has extensive heritage from seven space missions, including the Mapping Reflected-energy Spectrometer (MaRS, 2005) that enabled the Moon Mineralogy Mapper (M^3^) in 2008; the Mapping Imaging Spectrometer for Europa (MISE), the science instrument expected to reach Europa in 2024; and the two Carbon Mapper spectrometers (based on EMIT), the first recently launched in March 2024. It also has heritage from nine airborne programs including the Portable Remote Imaging SpectroMeter (PRISM) in 2012; the NSF NEON’s Airborne Observation Platforms (AOP) in 2013; the Compact Wide-swath Imaging Spectrometer II (CWIS-II) for the University of Zurich, 2022 [368]; and the AVIRIS-3 airborne IS that began flying in 2023 [37]. 

EMIT has recently gained significant attention for its ability to directly observe and monitor point sources for emission of powerful greenhouse gases, (CO, CO_2_, CH_4_), based on measurement of their spectral absorption patterns. However, these instruments have mapped many other materials including geologic minerals and geo-morphology, soil constituents, water in all three phases (vapor, liquid, ice), plant species distributions and habitat, biochemical traits in vegetation, and coral reef mapping.

#### 16.1.2. Surface Biology and Geology: SBG-TIR

The SBG-TIR mission aims to measure surface temperature and emissivity of the Earth (Table 20), measure evapotranspiration (ET) from vegetation and soils, provide identification of substrate composition based on the TIR spectrum, and measure and monitor high-temperature conditions such as fires and volcanic plumes [369]. These are critical Earth Science measurements as the planet warms and extreme heat and cold become more common and widespread. The measurement of ET is a direct way of assessing vegetation health or stress and serves as an indicator of potential plant productivity. It provides an independent measure confirming estimates of plant photosynthesis, because both ET and photosynthesis occur during daylight hours, and both require open stomates for gases to pass into and out of the interior of the leaf.

The SBG-TIR free Flyer (Table 20) (accessed on 13 May 2024)) is planned to include an 8-band mid IR-TIR imager, with bands between 3 and 12 μm, plus a two-band VNIR camera from Agenzia Spaziale Italiana (ASI), a partner with NASA on this mission (https://thermal2023.esa.int/iframe-agenda/files/presentation-235.pdf (accessed on 23 May 2024)). The imager will have two optical bands (VIS and NIR) at 30 m GSD for calculating the NDVI to map vegetation (https://database.eohandbook.com/database/missionsummary.aspx?instrumentID=975/ (accessed on 3 May 2024)). The TIR will fit within the fairing of a European VEGA-C or other light to medium class launch vehicle such as the PLATINO-2 (minisatellite platform) [369]. The VNIR camera will provide geolocation for TIR measurements and produce vegetation information using the NDVI bands.

The TIR is a whisk broom 8-band radiometer, with seven bands between 3 and 12 μm and an additional SWIR band at 1.65 μm (Table 20). The mission heritage is from the ECOSTRESS TIR imager (Section 9.5 [370]) on the International Space Station (Table 11). Eight TIR bands [369], make it possible to accurately estimate the emissivity of Earth’s surface materials, enabling more accurate calibration of the radiance data into temperature. It is currently targeted for launch in late 2028 and flown in a 13:30 (local) descending afternoon crossing time with a repeat frequency of three days. The early afternoon crossing time was chosen to provide better thermal coverage of the Southern Ocean and to optimize observations for active terrestrial wildfires, although at a loss of concurrent data collection with the SBG VSWIR instrument (90 min earlier). Additionally, the SBG-TIR with its 60 m GSD will contribute to high spatial information about other high-temperature conditions, e.g., vulcanology and volcanic eruptions [370], and wildfire risk [371], through higher vegetation temperatures and lower evapotranspiration. The co-hosted VNIR camera will provide geolocation for TIR measurements and vegetation input for evapotranspiration estimates.

### 16.2. Mass Change (MC) and Geosciences International Constellation (MAGIC)

The original DS design was for a pair of satellites, following the design of GRACE-FO [372], including use of parts left from fabrication of GRACE-FO, to keep costs low, with the main goal of ensuring continuity for GRACE-FO while the European’s were developing a subsequent improved satellite but with a likely launch time would leave a one-year or more data gap. Weise et al. [373] describes advances the community study team wanted for this mission (Table 20). The MAGIC mission is a joint venture between NASA and ESA to launch four satellites, operating in pairs to measure fluctuations in the Earth’s gravitational field. This program builds on earlier missions such as ESA’s Gravity field and steady-state Ocean Circulation Explorer (GOCE) and the NASA Gravity Recovery and Climate Experiment (GRACE) missions and its following mission GRACE-FO (see Section 5.1 and Section 5.2).

MAGIC replaces the 2017 NASEM recommendation for a GRACE-FO design with spare-parts, primarily to ensure continuity while keeping costs from substantially increasing. MAGIC will measure changes in the gravitational field, primarily due to changing water mass in the subsurface at much higher temporal and spatial resolution than GRACE-FO. In addition, this mission plans to provide information about sea-surface temperature, sea-surface heat flux, surface soil moisture, geoid shape, sea-level height, glacial motion, land crust motion, ice sheet topography, and soil moisture in the rootzone. The main instrument of MAGIC is the MicroSTAR, a much improved three-axis accelerometer, compared to those on GRACE-FO.

### 16.3. Surface Deformation and Change (SDC)

The goal of this mission is to better understand the dynamics of earthquakes, volcanoes, landslides, glaciers, groundwater storage and changes, and the deep interior. Its aim is to quantify the rates and driving processes of sea-level change and landscape change that could be used to provide support for better hazard forecasts and disaster impact assessments. Over the years since the DS in 2017, the SDC team and the science community developed a study plan that included an architecture review for trade space and assessed the international SARs of record (deformation measurements are typically made by Synthetic Aperture Radar Interferometry, InSAR). The InSAR technology is substantially more expensive than the original DS budget, although the instrument would be also much more capable. The study team worked to build community support and acceptance of decisions and priorities. Currently, however, the mission is on hold, waiting for lessons-learned from NISAR (Section 13.6) and the Copernicus Sentinel ROSE-L (Section 17.4).

### 16.4. The Atmosphere-Observing System (AOS)

**Figure 25:**
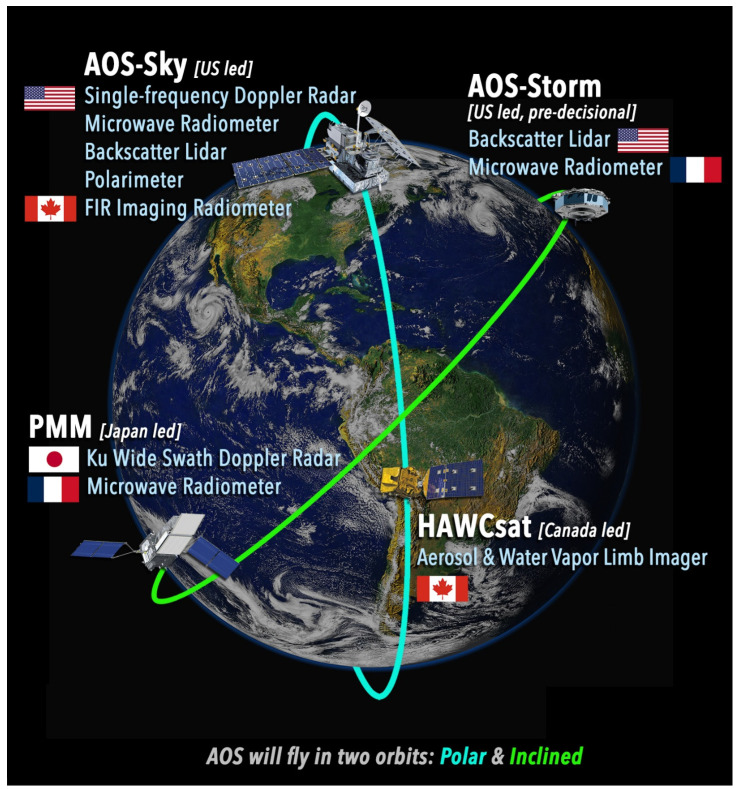
Artist’s rendition of the orbits and instruments, including country of origin, for the Atmospheric Observatory System (AOS) a constellation of four platforms in two orbits for precipitation, clouds, aerosols and dynamics, a NASA program implementing part of the NASEM DS instruments (figure from NASA Earth Sciences, AOS constellation image).

The original AOS mission goal from the 2017 NASEM DS was to improve understanding of aerosols, clouds, convection, and precipitation. The DS plan expected a single platform, not four satellites in two orbits, two instruments in sun-synchronous orbits and two in drifting orbits (Table 20). The current design is far beyond what was envisioned by the DS (Figure 25). The plans for AOS include contributions from collaborators among NASA, Canadian (CSA), and Japanese (JAXA) partners and contributions from other nations. The current estimate for launch of the first satellite is 2029.

The AOS SKY polar-orbiting sun-synchronous orbit at 450 km altitude would have a platform at an equatorial crossing at 13:30 with a likely lifetime from 2031 to 2036. It will host five instruments, a single-frequency Doppler Radar at either 94 GHz (W-band) or at 35 GHz (TBD), a Ka band (Sky-Radar) for characterizing precipitating clouds and related vertical air masses, and a Microwave Radiometer (Sky-MWR). The MWR is a passive Microwave Radiometer with GHz channels at 5 frequency ranges: 89–113, 186 (3 channels), 325 (3 channels), and 640–700 (2 channels). It also has an AOS-Lidar (nadir viewing: 532 nm and 1064 nm, both polarization sensitive), and an AOS multiangle polarimeter (AOS-Sky-polarimeter) to measure aerosol optical depth, aerosol absorption optical depth, and aerosol fine-mode effective radius. It is a polarimeter with nine bands in the 380–1570 nm range for aerosols. Bands are UV (350–390 nm), VIS (410–750 nm) for aerosols and bi-spectral clouds (in two channels), and hyper-angle (670–870 nm, 900–960 nm) for water vapor, SWIR (1350–1400 nm) for cirrus, and (870–1570 nm) for three bispectral cloud channels. Lastly, it would include a Thin Ice Cloud in the Far InfraREd region (TICFIRE), a nadir viewing 6 band radiometer from 7.5 to 50 μm and an accompanying imager to measure limb view and provide vertical structure.

In the same sun-synchronous orbit, altitude, and crossing time is the AOS HAWCSat platform, with the high-altitude aerosols, water vapor, and clouds satellite. It has two instruments, the Aerosol Limb Imager (ALI) and the Spatial Heterodyne Observation of Water (SHOW), a limb-viewing imager for the upper layers of the Troposphere measuring between 1362 and 1368.32 nm with a 64 km swath and a vertical resolution of 63 km.

The AOS-STORM platform is in a drifting 55° orbit at an altitude of 430 km and has two instruments: the AOS-Lidar (same as on Sky) and an AOS Microwave Radiometer (MWR, also called SAPHIRE-NG). This is a passive Microwave Radiometer with channels in 89–113 GHz, 183 GHz (6 channels), 325 GHz (3 channels) are similar to Sky MWR but it lacks high-frequency channels. The MWR will measure ice cloud properties and ice water path. It will measure 532 nm and 1064 nm, both with a polarization sensitive nadir lidar, the same as the Sky-MWR. This mission has a meteorology focus and measures precipitation with a Doppler (AOS Doppler) with a Ku band at 13.6 GHz that simulates convective vertical motions in tandem with AOS Storm. It is expected to provide the time rate of change and a vertical mass flux. Lastly, is an AOS MWR (SAPHIRE-NG)

## 17. The Copernicus Expansion Missions

**Figure 26:**
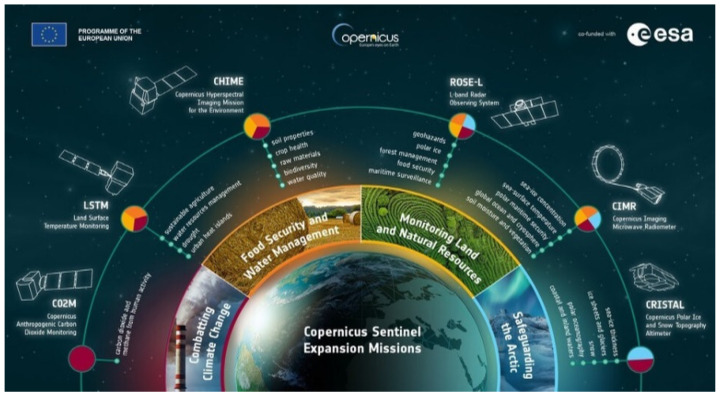
An artist’s rendition of the six Copernicus Expansion missions. The innermost ring identifies the general mission objectives of these missions. The outside ring identifies the main objectives of each instrument, and color-coded circles indicate the main mission functions: deep red for identifying carbon cycle compounds; blue for ice and snow; orange for soil moisture and crop production; and yellow for drought and high temperatures. Schematics on the outside display the prototype platform designs (Figure from the European Union Copernicus program’s expansion missions).

The original six Copernicus missions were developed by the European Space Agency (ESA) to address challenges of climate change and increasing populations that have stressed ecosystem services (Table 18). Following the launch of the first three of the six Sentinels, additional priority candidate missions were identified to address gaps in Copernicus user needs and to expand current capabilities. In 2021 six new “Expansion Missions” were identified (Figure 26) are: (1) the Copernicus Hyperspectral Imaging Mission for the Environment (CHIME), complementing Sentinel-2 with hyperspectral imaging observations, to support new and enhanced services for identifying and monitoring land and ocean properties; (2) the Copernicus Land Surface Temperature Monitoring (LSTM) mission, to obtain TIR imaging measurements to monitor vegetation evapotranspiration, predict droughts, and manage water resources for agricultural production, in the face of climate warming and variability; additional emphasis is placed on thermal conditions of coastal and inland waters, urban heat islands, land degradation, and detection/monitoring for natural hazards such as fires and volcanoes; (3) the Copernicus Anthropogenic Carbon Dioxide Monitoring (CO_2_M) mission to measure atmospheric carbon dioxide produced by human activity with a near-infrared and shortwave infrared spectrometer, to reduce current uncertainties in estimates of CO_2_ emissions from fossil fuels and track effectiveness of national decarbonization policies; (4) L-band Synthetic Aperture Radar (ROSE-L) providing surface information not accessible from the C-band Sentinel-1 data, with a longer-wavelength L-band signal that can penetrate through many natural materials (e.g., vegetation, and dry snow and ice) to support monitoring of soil moisture and subsidence, to support forest management, and to discriminate crop types for precision farming and food security; (5) Copernicus Imaging Microwave Radiometer (CIMR), a wide-swath conically scanning multifrequency Microwave Radiometer to provide observations of sea-surface temperature, sea ice concentrations, sea-surface salinity, and other sea ice parameters; and (6) Copernicus PolaR Ice and Snow Topography ALtimeter (CRISTAL), a dual-frequency radar altimeter and Microwave Radiometer to measure and monitor sea ice thickness and overlying snow depth, changes in ice sheets and glacier heights. Thickness of sea ice would support maritime operations in polar ocean regions.

### 17.1. The Copernicus Carbon Dioxide Monitoring Mission (CO_2_M)

The CO_2_M is the first of the Copernicus Expansion Missions (Table 21). CO_2_M will be the first mission to specifically measure CO_2_ emitted to the atmosphere by human activity. It is a three-satellite constellation in near-polar sun-synchronous descending LEO with estimated 11:30 Equatorial crossing times (https://space.oscar.wmo.int/satellites/view/co2m_a/ (accessed on 10 May 2024)). The three satellites will measure atmospheric constituents and properties, and be renamed Sentinel-7A (2026), S-7B (2027), and S-7C (2029) after their launches (https://www.esa.int/ESA_Multimedia/Images/2021/02/CO2M/ (accessed on 10 May 2024)). Together, these three will ensure accurate measurements of CO_2_ and other emissions, from major pollution emitters (e.g., cities, power plants, large industrial facilities). The program will use a mass-balance approach to estimate CO_2_ and NO_2_ emissions. Instruments aboard will measure Top-of-Atmosphere (TOA) radiances for CO_2_ and tropospheric NO_2_ using blue (405–490 nm) and VIS wavelengths (which also covers CHOCHO, glycoxal), NIR (743–773 nm), and water vapor with SWIR bands between 1590 and 1675 nm and 1990 and 2095 nm [374]. Related objectives are to accurately quantify annual emissions of gas plumes (CO_2_, CH_4_, NO_2_), with point-source CO_2_ emissions estimated with an uncertainty of ≤30% or less. Also to be measured are solar-induced chlorophyll fluorescence (SIF), aerosol optical depths (AOD), layer height, and water/ice cirrus clouds [375].

The CO_2_M has three instrument subcomponents. The first is a narrow-band hyperspectral NIR/SWIR imaging push-broom cross-track integrated CO_2_ and NO_2_ imaging spectrometer (CO_2_I and NO_2_I) (https://space.oscar.wmo.int/satellites/view/co2m_a/ (accessed on 10 May 2024)) for estimates of anthropogenic emissions (https://space.oscar.wmo.int/instruments/view/co2i_and_no2i/ (accessed on 10 May 2024)). It will measure total column CO_2_ with precision of 0.7 ppm; CH_4_ with precision of 10 ppb, NO_2_ with precision of 1.5 × 10^15^ mole fraction/cm^2^, and TOA Solar-Induced Fluorescence (SIF) at 0.6 m W m^−2^ sr^−1^ nm^−1^, all at a spatial resolution of 4 km × 4 km (https://www.eoportal.org/satellite-missions/co2m/ (accessed on 6 may 2024)).

There will be two other instruments on board these satellites. The three-band CLoud IMager (CLIM) is a push-broom multipurpose imager with GSD <0.4 km × 0.4 km and a swath of 465 km (https://space.oscar.wmo.int/instruments/view/clim/ (accessed on 6 May 2024)). The CLIM will detect low and high clouds in the spatial sample measured by the CO2I, to allow removal of cloud effects in the trace gas retrievals. The third instrument is the Multiangular Multi-band Polarimeter (MAP) with seven narrow-spectral VNIR bands that support the CO_2_ and CH_4_ retrievals by accurately estimating the effective light path length effects of the aerosols. MAP is composed of four cameras with 12 FOV each, defined by detector pixels imaged onto the ground scene in different directions; each viewing direction will measure spectral radiance in seven wavelength windows and three polarization angles (https://space.oscar.wmo.int/instruments/view/map/ (accessed on 6 May 2024)).

### 17.2. The Copernicus Hyperspectral Imaging Mission for the Environment (CHIME)

The **C**opernicus **H**yperspectral **I**maging **M**ission for the **E**nvironment (**CHIME**) will launch a constellation of two IS satellites, CHIME-A and CHIME-B, which will have design lives of six years. CHIME will routinely provide hyperspectral observations to complement the Sentinel-2’s land-cover mapping priorities and to provide observations that support biodiversity, ecosystem and forest management, sustainable agriculture, water quality, and other essential applications. Each CHIME satellite (https://www.esa.int/Applications/Observing_the_Earth/Copernicus/Going_hyperspectral_for_CHIME (accessed on 11 My 2024)) will fly in the Sentinel-2 orbit carrying a unique push-broom VSWIR grating spectrometer that has a high signal-to-noise ratio across wavelengths and maintains data uniformity across the satellite’s 130 km swath. The two CHIME imaging spectrometers will have ≥200 narrow (≤10 nm) VSWIR spectral bands between 400 and 2500 nm (Table 21). CHIME-A was originally planned for launch in 2029 (Schimel et al. 2022, but launch date is under review) into the polar LEO Sentinel-2 orbit [376,377].

CHIME will have a similar capacity as the SBG-VSWIR spectrometer to measure the reflected solar spectrum, collecting spectra at 30 m or better GSDs (some wavelengths may have higher spatial resolution (≤30 m) but final decisions have not been announced). Original plans were to overlap the mission with Landsat’s-8/-9, and to fly in coordination with the NASA SBG-VSWIR mission (Table 11) and/or Landsat Next (Table 19), but plans are currently uncertain (https://database.eohandbook.com/database/missionsummary.aspx?missionID=1047/ (accessed on 12 May 2024)). NASA and the European Union have been coordinating their respective plans for these high-priority global mapping IS missions, with the goal of ensuring these instruments create an integrated Earth System measurement capability. Uncertainty developed when NASA recently delayed the SBG-VSWIR mission (until end 2032), which had been planned for launch into the Landsat orbit in 2028, followed in 2029 by CHIME-A (and CHIME-B in 2031) into the Sentinel-2 orbit. This approach would create a virtual constellation operating in the 10:00–11:00 time frame, comprised of CHIME and SBG-VWIR, along with Landsats-8 and-9 and a Sentinel-2 pair, to build a dense Earth archive for environmental and climate-impact investigations.

### 17.3. The Copernicus Land Surface Temperature Monitoring Mission (LSTM)

The Copernicus Land Surface Temperature Monitoring (LSTM) satellite is a recent addition to the Sentinel Expansion Missions (Table 21) with a mission to map surface temperatures and evapotranspiration rates every 1–3 days at 400-fold finer resolution than currently possible. The LSTM consists of a two-satellite constellation (LSTM-A and LSTM-B) to fly in near-polar sun-synchronous LEO at 651 km between −56° and +84° latitudes with a 13:30 crossing time (https://space.oscar.wmo.int/satellites/view/lstm/ (accessed on 4 May 2024)); (https://thermal2023.esa.int/iframe-agenda/files/presentation-231.pdf/ (accessed on 4 May 2024)). The wide 670 km swath of these thermal imagers, designed to increase repeat crossing frequency, prevents a push-broom approach so a whisk-broom scanning scheme was adopted with an average 50 m GSD. Launch of LSTM-A is expected to occur in the same time frame as the NASA SBG-TIR mission, which currently is scheduled for 2028.

The LSTM imaging instrument has eleven bands. The five TIR bands are the primary bands, with precision of 0.3 °C (8.6 µm, 8.9 µm, 9.2 µm, 10.9 µm, and 12.0 µm) to support relevant societal application investigations, specifically water management, sustainable agriculture, and land use within a temperature range between −20 °C and +30 °C. Secondary image bands include five VNIR bands (490 nm, 665 nm, 865 nm, and 945 nm) and two SWIR bands (1.38 µm and 1.61 µm). These secondary image bands support the global monitoring and mapping of soil properties, which are essential land surface characteristics linked to the primary mission goal of supporting sustainable agricultural soil management.

Soil fertility is an important component of sustainable land management, with measurement of properties like grain size distribution, mineral composition, soil organic carbon content. The TIR spectrum has diagnostic spectral features for rock and soil forming minerals, including quartz, feldspars, olivines, and pyroxenes, which are measurable with these secondary mission bands, and should enable the monitoring of changes in soil properties that indicate soil degradation. The secondary TIR bands that enable detection of soil properties includes absorptions at these bands: S1 (8.2 μm for Quartz); band S7 (9.0 μm for Kaolinite); band S11 (12.3 μm for Goethite); band S12 (9.3 μm for Montmorillonite); and band S13 (0.53 μm for Feldspar). TIR emissivity observations include soil geochemical properties (silica, Al_2_O_3_, Fe_2_O_3_) for clay minerals (2:1 minerals) and iron oxides (goethite) with soil texture (sand content).

Surface urban heat island (SUHI) monitoring is another secondary goal of the LSTM mission, which requires land surface temperature (LST) maps at approximately the 50 m GSD scale measured immediately before sunrise. The LSTM mission has the potential to make nighttime measurements on the ascending track around 01:30. Permafrost monitoring also requires high-spatial-resolution data due to surface heterogeneity. These measurements require LST data to be combined with digital elevation map (DEM) and meteorological data (e.g., reanalysis data, Westermann et al. 2015). Locations with ground movements, such as frost heaving, could also use InSAR data to reveal surface activity of some landforms such as rock glaciers that are typical of permafrost and related processes.

Combining LSTM data with Landsat-8/-9 TIR and SBG-TIR datasets will provide TIR measurements at five different midday times: two at 10:00 (L-8&9), one at 12:30 (SB-TIR), and two at 13:00 (LSTM-A, -B), offset in a 1–3 day repeat pattern. This LSTM image collection will complement and support the Sentinel-2 mission and the two upcoming full-spectrum VSWIR imaging spectrometer missions (CHIME and SBG-VSWIR), none of which have TIR capability.

### 17.4. The Copernicus Observation System for Europe, ROSE-L

Rose-L (A, B) is a high-priority mission for land surface monitoring (Table 22). It is an R&D L-band SAR with multipolarization and interferometric capability and off -nadir views to complement the Sentinel-1 C and 1D satellites. Rose-L-A (2030) and Rose-L-B (2032) will carry identical L-band SAR (1.2675 GHz) instruments that can penetrate deeper into plant canopies, snow, and ice fields than the C-band flown on Sentinel-1. Rose-L -A and -B will each operate for seven years, with a primary mission to support forest management, discriminate crop types and to monitor soil moisture and subsidence. This mission is complementary to the Copernicus CIMR mission, contributing to the monitoring of polar ice sheets, ice caps, and sea ice extent, maritime surveillance and geohazards. This mission will have the largest planar antenna (11 m × 3.6 m) of any satellite.

### 17.5. The Copernicus Imaging Microwave Radiometer (CIMR)

The CIMR will fly two satellite missions (2029, 2031), each carrying a wide-swath conical-scanning total-power 5-band (L, C, X, Ku, Ka) multifrequency passive Microwave Radiometer (Table 22). The CIMR imager is a high-priority mission for twice daily ocean observations, including sea-surface temperature (SST), sea-surface salinity (SSS) and sea ice concentrations (SIC) in polar regions, leading to improved understanding of changing arctic conditions [378]. This mission follows the CryoSat SIRAL mission, with IRIS, a dual-frequency (Ku and Ka) altimeter and a 3-frequency Advanced Microwave Radiometer (AMR)-CR, with heritage to Jason -2 and -3. It also includes three additional instruments: a high-frequency HRMR, a Laser Reto-Reflector (LRR), and GPS for accurate location. CIMR will provide high-spatial resolution microwave imaging radiometry measurements and derived products with continuous global coverage and sub-daily revisit in the polar regions and adjacent seas [379]. Additional products include sea ice thickness, sea ice drift, sea ice surface temperature, and snow depth on sea ice [380]. CIMR will have the capability to identify ice types, wind speed over the ocean and on land, terrestrial snow extent, and vegetation indices that contribute to measuring soil moisture properties, along with Rose-L.

### 17.6. The Copernicus Polar Ice and Snow Topography Altimeter (CRISTAL)

CRISTAL-A (2028) and CRISTAL-B (2031) will each have two instruments. The first is the IRIS (**I**nterferometric **R**adar **A**ltimeter for **I**ce and **S**now) with a Ku band (12.5 to 18 GHz) plus a Ka band (26.5 to 40 GHz) for snow depth retrieval (Table 22). The IRIS will support measurements for sea ice cover, edge and thickness, ocean surface winds, ocean wave height and spectrum, and ice sheet topography [163]. The second instrument is the AMR-C (Advanced Microwave Radiometer for Climate), a non-scanning MW radiometer with three channels. The IRIS is intended to produce multipurpose imagery for land, and both IRIS and AMR-C will measure atmospheric humidity fields, ocean topography and currents (Table 22). Together, both instruments will provide ice and snow classifications, wet troposphere correction for atmospheric humidity fields and ocean topography, and currents. This mission has heritage from Cryosat-2, with enhanced performance for monitoring sea ice thickness and land ice elevations.

## 18. The Satellites Coming in 2025 and into the 2030s

Five more instruments have been announced and are planned to fly in the early 2030s. Of these, only the WildfireSat Canadian satellite does not have preceding heritage sensors. Most of these new instruments/platforms do not have all their technical specifications fully released at this time. They are included here because the announced launch dates are within the coming decade. Sources include instrument sites, OSCAR, Copernicus, and the EO Portal.

We include the JAXA GOSAT-GW (expected launch 2025), an R&D Tanso-2 instrument measuring greenhouse gases and water cycle parameters, the Advanced Microwave Scanning Radiometer (AMSR3). The Canadian WildfireSat (expected launch ~2029) covers an urgent need for more information about wildfire conditions. It is not fully described (likely multiband TIR with some VNIR bands, and high spatial resolution, flown in a late afternoon, 15:00–18:00 period). The choice for polar or GEO has not been resolved. The Canadian space agency is working with vendors to develop a mature design. Landsat Next to be called Landsat-10 after launch. Landsat-8 and L-9 will be replaced by three platforms flying at 120° apart in the Landsat orbit to provide 6-day repeat coverage and crossing time at 10:00. The LandIS imager will have 26 spectral bands, with 5 bands 10 m pixels and 10 bands at 20 m GSD, and 3 SWIR bands at 30 m GSD, 5 TIR bands at 60 m GSD and plus 3 bands for calibration at 60 m pixels. In the 2029–2030 expected launch, Landsat-9 will still be in orbit, the Sentinel-2’s will be in orbit, the NASA-SBG VSWIR and the Sentinel CHIME (A and B) will likely be flying [381]. TRISHNA, LSTM, and SBG-TIR will be in the 13:00 orbit. The next two new programs are in GEO orbit, the Himawari-10, and GOES-XO. Plans for the Himawari 10 (~2099 launch) are mature and they plan to expand their imaging system, the Geostationary Himawari Imager (GHMI) to 18 bands (from 16), two VNIR bands at 0.5 km and four at 1 km GSD, a cirrus cloud band, and 12 bands in the SWIR, MWIR, and TIR. The second instrument is the GEO HIMawari (hyperspectral) Sounder measuring vertical distributions of moisture, winds, and temperature. The NOAA GOES-XO (~2033 launch) proposes a three-satellite system (east, west, and continental). NOAA has announced that the next ABI will have two additional bands at 910 nm and 5.15 μm for better measures of water vapor in the lower troposphere.

### 18.1. Japanese GOSAT-GW

The plans for the next Japanese GOSAT (GOSAT-GW), scheduled for a 2025 launch (Table 23). It will be in a polar sun-synchronous orbit, at 666 km altitude, and have selected swath widths (with low and high-resolution options). It is expected to have a 3-day repeat at the lower resolution and a 13:30 descending passing time. Plans are advanced in terms of the orbit but show the characteristics of the instrument’s measurements are not yet decided despite a 2025 launch date. Because the plans are not finalized, we have kept it with other instruments scheduled for later launches and whose descriptions are also not fully finalized.

### 18.2. WildFireSat (Canada/Planning Phase)

WildFireSat is a new program designed to improve management of forest and forest fires in Canada [382]. Tiaga and tundra forests cover 4 M km^2^ in Canada and are subject to large and intense wildfires (megafires), burning approximately 2.5 M Ha of forest annually [383].

The Canadian WildFireSat program (Table 23) will consist of one or more satellites equipped with infrared sensors that will measure the energy emitted by wildfires and detect smoke air pollution, and give accurate data on carbon emissions from wildfires, with a launch possibly as early as 2029. This energy emitted by fires is referred to as Fire Radiative Power (FRP). With FRP information, essential characteristics of wildfires such as fire intensity and rate of spread can be derived.

The goal of WildFireSat is to support Canada’s wildfire management and provide more precise information on smoke and air quality, while at the same time, accurately measuring carbon emitted by the fires. After launch it will become part of a global network of satellites used to monitor wildfire conditions [384,385]. More accurate measurements of fire temperatures will contribute to improved carbon emission monitoring. We include it in this paper, given the timing of the proposed launch 2029 date but note that the configuration of the satellite(s) is/are not yet finalized.

### 18.3. The Fourth-Generation HIMAWARI-10 Geostationary Satellite

The Japan Meteorological Agency (JIMA) has identified a fourth-generation multipurpose geostationary satellite to improve nowcasting, Numerical Weather Prediction (NWP) assimilation, and environmental monitoring. The new system will improve wind derivation by tracking clouds and water vapor features (Table 23). This new system will host the most advanced instruments in geostationary orbit, improving upon the Himawari-8 and -9 satellites. The Himawari-10 is planned to be launched in 2028 and become operational in 2029 with a 15^+^-year expected lifetime. It will host the Geostationary HiMawari Imager (GHMI) which will have 18 bands across the VSWIR, MWIR, and TIR (Table 22), replacing the AHI on Himawari-8 and -9 (https://space.oscar.wmo.int/satellites/view/himawari-10/ (accessed on 26 April 2024)). It will also have a new sounder, the Geostationary HiMawari Sounder (GHMS). The GHMS will measure high-spectral-resolution infrared radiances to collect vertical information on atmospheric temperatures and water vapor, which will improve weather forecasting when assimilated into numerical weather prediction models (https://global.jaxa.jp/projects/sat/gms/ (accessed on 26 April 2024)).

The GHMI design is based on the same concept that NOAA is developing for an improved Advanced Baseline Imager (ABI) now operating on GOES 16 and 18 satellites. The collaboration between JIMA and NOAA continues to support the parallel development of current imagers on the Himawari 8 and 9 and the GOES 16 and 18.

### 18.4. The Fourth-Generation NOAA GeoXO Imager (GXI)

NOAA is developing plans for GeoXO, its next-generation satellite that will produce a real-time, high-resolution visible and infrared imagery in real time for monitoring Earth’s weather, ocean, and environment in the next set of Geostationary satellites (Table 22). The GeoXO Imager (GXI) will be an 18 multichannel passive imaging radiometer that will observe the Western Hemisphere and continue to provide variable area imagery and radiometric information about Earth’s surface, atmosphere, and cloud cover. It will have two bands more than the current ABI (at 910 nm and 5.15 μm, adding information on water vapor in the lower Troposphere, near the surface). These bands are close to the 860 nm band on the GHMI and match their MWIR band at 5.15 µm. Adding these channels helps to fill in measurements across the electromagnetic spectrum from the visible through thermal infrared.

The new GXI will improve spatial resolution of two VIS channels, three NIR channels and two MWIR channels. The 640 nm band will have 250 m GSD, compared to 500 m on the current ABI. The GXI will also reduce the spatial resolution of the 3.9 µm band from 2 km to 1 km. That band is important for fire detection, and the improved spatial resolution will increase detection of fires 4-fold smaller than is currently possible. The new imager is planned to provide broad improvements in cloud detection, and retrievals of cloud properties, water vapor, aerosols, and dust in the atmosphere. It will improve measurements for vegetation and land surface conditions due to higher spatial resolution of its key bands. It will improve measurements made of land and sea-surface temperatures, including volcanic ash, fires, snow, and ice cover.

The GeoXO East (2032–2047) and GeoXO West (2035–2050) will carry—in addition to the GXI’s, a new Lightning Mapper (LMX) and an Ocean Color Instrument (OCX), but also a data collection and integration system (DCIS). There will be a new third geostationary satellite, GeoXO Central, that is also planned for launch in 2035, carrying the GXS, an interferometer measuring two bands at approximately 0.5 cm^−1^ (or better) and has two proposed bands from 670 to 1100 cm^−1^ and 1700 to 225 cm^−1^ for more accurate measurements of atmospheric temperature and humidity. The GXS is expected to have higher spatial resolution than current sounders, at 4 km. It will also carry the Atmospheric Composition Instrument (ACX) for observation of atmospheric pollutants (gases, aerosols, dust) from different point sources.

The GXS will follow the TEMPO (Tropospheric Emissions: Monitoring of Pollution), a NASA Earth Venture Instrument (https://science.nasa.gov/mission/tempo/; https://tempo.si.edu/ (accessed on 29 April 2024)), launched in GEO in 2023. TEMPO is a passive grating spectrometer measuring the UV (293–494 nm) and VIS (538–741 nm) wavelength regions with 0.2 nm spectral sampling. It has 2.0 × 4.75 m spatial resolution and will measure in different modes at intervals from minutes to hours. It will also make frequent observations of O_3_, NO_2_, SO_2_, HCHO, C_2_H_2_O_2_, aerosols and cloud UV-B. Earth Venture instrument, TEMPO promises to enhance diurnal observations of carbon, water, and heat processes from GOES orbits [281].

### 18.5. The Next Landsat: “Landsat Next” (the Future Landsat-10)

The next Landsat in the U.S. Sustainable Land Imaging Program will be “Landsat Next” (becoming L-10 after launch). Although Landsat Next’s launch is currently positioned near the end of the queue for new satellites (2030+), its importance to the continuity of the Landsat program cannot be understated. In addition, it will have an essential role in the virtual NASA/ESA EO constellation for moderate spatial-scale global images [386].

The Landsat Next program (to be renamed Landsat-10 at launch) will have a three-satellite constellation, each located 120° apart (Table 23), with a mission design life of five years on orbit, with a launch anticipated for late 2030 (https://landsat.gsfc.nasa/gov/satellites/landsat-next (accessed on 29 April 2024)). Landsat-10 will occupy a lower orbit at ~653 km than previous and current Landsats at 705 km. With a 98^o^ inclination angle, Landsat Next will provide somewhat smaller scenes (164 km × 168 km) with 18-day individual repeat observations, and 6-day repeats with all three instruments. In contrast to previous Landsats it will have an equatorial crossing time of 10:10 ± 5 min. Given the successes of several current (and future) hyperspectral imaging spectrometer satellite missions, the Landsat program is pivoting from its largely thematic land cover approach to embrace more discoveries with narrower and more spectral bands. The Landsat program [39,181,220,387] described enhancements needed to advance the next generation of science and applications for Landsat, including additional narrower bandwidths coupled with higher GSD of 10–20 m pixels for some bands. These spectral/spatial advances will allow more specific interpretations of land cover composition.

**Figure 27:**
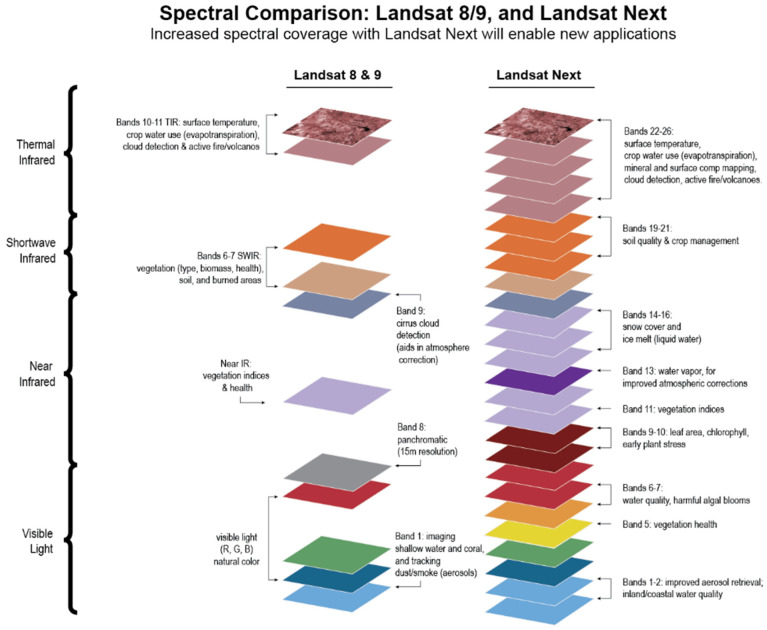
Comparison of Landsat-8 and -9 bands and proposed Landsat Next bands. The proposed system increases the number of bands to 26 from the current eleven. Bands 104 for Landsat 8 and 9 and 1–10 (for Landsat-Next) are colored to represent the color of the band in the visible spectrum. Band numbers higher than this are in the near infrared or thermal infrared and cannot be seen with the human eye and so the colors are arbitrary; bands with wavelengths close together are represented in different shades of a color. This figure shows which bands are introduced in Lsandsat -next and which have changed from Landsats -8 and -9 (figure from NASA/USGS Landsat missions 11 January 2023).

The Landsat Next will measure 20 bands in the reflected solar spectrum (between 410 and 2250 nm), compared to nine in L-8 and -9, and with five bands in the TIR spectrum compared to two bands for L-8 and -9 (Table 24, Figure 27). Except for the new broad NIR band, most are narrower bands, centered at important wavelengths for spectral absorption features, compared to L-8 and -9.

This enhancement in spectral resolution, coupled with five VNIR bands acquired at higher 10 m spatial resolution and ten additional bands with 20 m GSD, will provide data products that resemble those already available from the ESA’s Sentinel-2 satellites (Table 8). This change in spatial GSD resolution will require a new descriptive spatial reference system, the 3rd version of the World Reference System, WRS-3. These enhancements will enable more confidence in identification of specific land cover types (e.g., forests vs. grasslands) and their dominant species (e.g., oaks vs. pines), and will enhance the ability to identify both the status and changes in vegetation health and vigor. In addition, Landsat Next will include five TIR bands at 60 m GSD, compared to the two on L-8 and -9 at 100 m GSD. This will enable separation of thermal and emissivity properties based TIR radiance values for the first time, using the widely cited method of [388,389]. This capability will significantly contribute to more accurate estimates of surface temperatures and emissivity, and thus lead to better monitoring of urban heat islands, wildfires, and evaporation in agricultural crops and natural ecosystems. This evolution in the Landsat program marks a transition from previous multispectral imagers with just a few bands and aiming towards a full imaging spectrometer, with a complete spectrum of 100 s of contiguous bands (Section 3.4, Section 4.2.2, Section 8.1, Section 8.2, Section 9.1 and Section 9.4). The science benefits of continuous spectra acquired from space were first demonstrated by the EO-1 Hyperion instrument [81,82] and by data collected by the recently launched European satellites, PRISMA and EnMAP (Section 7), as well as the state-of-the-art imaging spectrometer, EMIT (Section 9.4). Given programmatic delays, the Sentinel Expansion CHIME mission (Section 16.2) will most likely precede or become contemporary with Landsat Next.

## 19. Summary

The evidence from the last 50+ years of space-based remote sensing data for Planet Earth has shown an astonishing amount of change from the dual impacts of human-caused changes in the natural environment and those secondarily caused by humans emitting greenhouse gases into the atmosphere, of which approximately half remain, and which are having additional impacts caused by the radiative consequences of their higher atmospheric concentrations. A recent estimate from Our World Data (https://ourworldindata.org/greenhouse-gas-emissions/ (accessed on 16 May 2024)) using original data [390] reports that the GHG CO_2_ equivalent (for NO_2_, CH_4_, and other trace gases) has increased from 29.13 B Tons CO_2_ in 1972 (at the dawn of EO remote sensing) to 54.59 GT in 2023 [391]. Determining the point sources and vulnerable places globally, while continuing to monitor over time, will continue to produce enormous amounts of biogeophysical information to capture evidence of impacts, which is necessary to better understand linkages among global biogeochemical processes. Reducing the destructive impacts of climate change and human exploits on the Earth’s natural resources (land, air, and seas) on which we all depend will require unprecedented international cooperation; collaboration among nations and among space agencies and environmental consortia is essential for this to happen. This commitment is necessary to continue to acquire essential information for policy and informing the public that is best acquired from space-based perspectives, and to confirm effectiveness of the remediation and protective actions undertaken, while avoiding increased degradation. This may best be supported by imaging spectrometers (≤50 m GSD); however, their datasets will enormously expand the global collection archives relative to Landsat-8 and -9. NISAR is expected to produce (www.earthdata.nasa.gov/learn/articles/getting-ready-for-nisar/ (accessed on 16 May 2024) approximately 10 fold the data volume that SBG-VSWIR will produce (https://www.nas.nasa.gov/SC21/research/project26.html/ (accessed on 16 May 2024)). NISAR satellite is expected to produce 140–150 PB in its 3 years of initial mission operation [349]. For the wealth of new and continuing data from satellites in space, we will lose their value if we can’t process and analyze the data to create actionable management and stewardship of the Earth.

We have provided an extended survey of the satellites that collect the data most relevant for addressing the wide scope of necessary environmental research. We included satellite instruments collecting data over a wide range of spatial scales, from a meter or a few meters up to a few kilometers. We included imaging and non-imaging instruments that measure across the electromagnetic spectrum, from the UV through visible, near infrared, midwave infrared, thermal infrared, far infrared out to wavelengths approximately 50 µm, and in radar and microwave bands from W to P band (millimeter to meter scales). We included satellites representing all types of orbital conditions, including different orientations, altitudes, and equatorial crossing times. We have also provided a description of the primary instruments of each satellite, and summarized how their data are used. We provided 20 tables that highlight the technical characteristics of each of the satellites and their primary instruments or those of most interest to the EO community. Also included are lengthy descriptions of important past satellites (the pioneers) and we have highlighted the heritage of newer instruments.

Because of the relatively short flight period of most instruments and their satellites (from three or five years, or rarely, a decade or at most two), time series studies require the integration or joining of data acquired by several satellites together, most likely of different types, that can form a 20- or 30-year or 50-year history. Another reason for discussing the pioneers was that we felt it was important to have some understanding of the precursor instruments and how datasets have changed over various time periods. This includes changing the wavelengths, the number and width of spectral bands, the acquisition time of day, the spatial resolution, the viewing angle, atmospheric correction certainty, and other significant factors (e.g., fidelity, SNR, processing algorithms), from the data characteristics found in many heritage datasets. Additionally, the way the instruments are configured, and the types of detectors or other instrument components can have significant impacts on interpreting the trends in time-line data. As well, interpretation of these apparent trends also needs to consider the analytical methods that were used, which have clearly undergone substantial evolution over this fifty-year time-period.

Despite the length of this paper, we have by necessity only provided a minimum amount of information on each instrument that we included. But we have provided links to data sources in the text and provide a long list of data sources in Section 1.3 of this paper to assist readers who want more depth on any of these instruments. We note that we encountered many examples where descriptive information differed, depending on the source (e.g., websites), and found it was sometimes difficult to determine precisely what the correct information is because much of it is not dated. This differing information according to source applies to many factors that describe an instrument or satellite’s key characteristics, and can be confusing, but we have made our best effort to present consensus information. Therefore, the included technical information is the best publicly available at the time of publication of this paper. We are hopeful that the reader will forgive any mistakes or omissions and will be encouraged to do their own investigations of those instruments and satellites that most intrigue them.

For missions in the pipeline now, the reality of information for instruments and satellites under fabrication, or not yet built, is that they may likely change. There are many reasons for this, such as discovering that essential components fail to meet required specifications during the mission review period, or the tests conducted prior to launch indicate their performance is not as expected (sometimes it is better, not always worse). Sometimes cost overruns (e.g., from delays in the schedule) require descoping decisions and sometimes unexpected glitches at launch may result in an underperforming instrument. Thus, keep in mind that all technical descriptions for satellites not yet in orbit can change and those in orbit can have post launch failures, and satellites can lose altitude because of drift or intentional movement. But on average, most satellites and instruments have been generally hardy and many more function well beyond their expected flight durations. Also, there is a constant tug of war between supporting the cost of old (beloved) instruments long past “retirement age” and funding new experimental or operational instruments. And sometimes it is more difficult to extend well-functioning satellites into “long-term” and “operational” status than to shift to a new satellite’s measurement mode that will require new algorithms and archival procedures, and community acceptance.

Nevertheless, we anticipate that our review will be useful in educating the remote sensing and environmental communities about the EO assets available to them and instill some urgency in finding ways to integrate the available datatypes into revealing action-inducing progress. In that regard, we implore those with the purse strings to allocate considerably more resources towards data integration, possibly with new initiatives that incorporate Artificial Intelligence and other sophisticated approaches for blending disparate data types.

## Figures and Tables

**Table 1:** Relating general radar frequency es and their associated wavelengths, and naming convention for wavelength bands.

Band	W	Ka	K	Ku	X	C	S	L	P
Frequency, GHz	75–100	40–100	18–26.5	12.5–18	8.0–12.5	3.90–8.0	1.55–3.90	0.39–1.55	0.216–0.45
Wavelength, cm	0.27–0.40	0.30–0.75	1.67–1.11	1.7–2.4	2.4–3.75	3.75–7.5	7.5–5	15–30	30–130

1 GHz = 10–9 Hz.

**Table 2:** NOAA satellites starting in the late 1970s and continuing into current satellites. The NOAA satellites carried versions 1–3 of the Advanced Visible High-Resolution Radiometer (AVHRR), and the METOP-A, B, and C satellites carry AVHRR-3 with red and NIR bands that produce the long-term NDVI data record used for global environmental change research. Technical descriptions are from NOAA AVHRR history and OSCAR, a web resource developed by the World Meteorological Organization (WMO) for earth observations.

Satellite/ Instrument	Launch Date(s)	Orbit Type	Altitude, km	Swath, km	Equatorial Crossing Time	UV 350–400 nm; VNIR 400–1500 nm; GSD km	SWIR, 1500–2500 nm, GSD km	Mid-IR 3.0–6.0 μm, TIR, 6–15.0 μm, GSD km
**NOAA** TIROS-N AVHRR-1	1978–1981	polar, LEO Sun synchronous	850	2900	14:30A	615 nm, 912 nm @ 1.1 km	--	3.74 μm 11.0 μm @ 1.1 km
**NOAA** 6, 8, 10 AVHRR	1979–1987 1983–1985 1986–2001	polar, LEO Sun synchronous	840, 820 810	~2700	14:30A 07:30D 07:30D	615, 912 nm @1.1 km	--	3.74 μm, 11.0 μm @1.1 km
**NOAA** 7, 9, 11, 12, 14 AVHRR/2	1986–2001 1988–2004 1991–2007 1994–2007	polar, LEO Sun synchronous	860, 850 843 804, 844	3000	14:30A 14:30A 04:10A 05:10A 09:30A	615, 912 nm @1.1 km,	--	3.74, 10.8, 12.0 μm @1.1 km
**NOAA** 15,16, 17, 18,19 AVHRR-3	1998–2024 2000–2014 2005–2024 2009–2024	polar, LEO Sun synchronous	813 810 854 870	3000	07:29D 09:03D 10:34D 08:22D	630, 862 nm @1.1 km	1.61 μm @1.1km	3.74, 10.80, 12.00 μm @1.1 km
**METOP-A, B, C**, AVHRR-3	2006–2021 2012–2024 2018–2027	polar, LEO Sun synchronous	827 827 817	3000	07:50D 09:31D 09:31D	630, 862 nm @1.1 km	1.61 μm @1.1 km	3.74, 10.8, 12.0 μm @1.1 km

**Table 3:** Pioneering instruments, mostly from the 1990s or later versions of an earlier instrument. The ATSAR and GOME instruments were on ERS-1 and -2, the GOME-2 was also carried on the METOP A, B, C series (Table 2). ATSAR’s SWIR, MWIR, and TIR bands provide atmospheric measurements along with a new space technology, the 2-band microwave sounder for water vapor, which was continued as GOME-2 on the METOP A-C series. The CHRIS instrument was the second hyperspectral imager in space (after EO-1/Hyperion), flown on the PROBA-1 satellite and had unique option for selectable bands and spatial resolution choice.

Satellite Instrument	Launch Date	Orbit Type	Altitude, km	Swath, km	Repeat Frequency, Days	Equatorial Crossing Time	UV 240–400 nm, VNIR, 400–1500 nm, SWIR 1500–2500 nm; GSD m	MidIR 3.0–6.0 TIR 6.0–15.0 μm, GSD	Radar and Microwave
**ATSR/ERS 1 ATSAR-2/ERS-2**	1991–2000, 1995–2008	Polar, LEO Sun synchronous	785	500	3 d TIR & 6 d SWIR, 3 d NIR & 6 d VIS	10:30D	ATSR-2: 550, 659, 865 nm; ATSR-1 &-2: 1610 nm @ 1 km GSD	ATSR-1 & -2: 3.70, 10.85, 12.00 μm @ 1 km GSD	ATSR-1, microwave sounder 23.8, 35.6 GHz @ 1 km GSD
**GOME/** **ERS-2**	1995–2008	polar LEO Sun synchronous	785	120 or 960	3 d NIR & 6 d VIS	10:30D	[240–295, 290–405, 400–605, 590–790 (1024 channels)], [292–402, 402–597, 597–790 nm (pol, 1 channel)] @ 40 × 40 km^2^ @ larger swath, or 40 × 320 km^2^ @ smaller swath	--	--
**CHRIS/PROBA-1**	2001–2022, 2001–2024	polar LEO Sun synchronous	615	Mode 1–4 13 km, Mode 5 6.5 km	7 d	07:30D	400–1030 nm, 150 channels, selectable Mode 1 up to 63 channels over 5 angles (−55, −36, 0, 36, 55) @36 m GSD. Mode 2–4, 18 bands @ 18 m GSD, Mode 5, 37 bands @ 18 m GSD.	--	Single frequency Ku band 13.575 GHz and bandwidth 350 GHZ. @15 km GSD in SAR model, along track resolution is 250 m.

**Table 4:** The 11 instrument Envisat platform provided land, oceans, and atmospheric measurements, complementary to those on the NASA EOS platforms. The primary instruments for EO are the MERIS, a general-purpose imager for monitoring terrestrial and ocean conditions, and the SCIAMACHY, an early instrument for monitoring atmospheric chemicals of concern for greenhouse gases and pollution and the JAXA GOSAT platforms-1 and -2, for measuring carbon-based greenhouse gases and The Landsat-7 Enhanced Thematic Mapper (ETM+). Technical descriptions are from OSCAR, ESA Earth Online, EO Portal, and mission websites.

Satellite /Instrument	Launch Date	Orbit Type	Altitude, km	Swath, km	Repeat Frequency Days	Equatorial Crossing Time	UV, VNIR, 400–1500 nm; GSD, m	SWIR, 1500 2500 nm, GSD m	Thermal IR 5.5–15 μm
**MERIS (Medium Resolution Imaging Spectrometer) ENVISAT**	2002– 2012	Polar, LEO Sun synchronous	774	1150	1–3 d	10:00D	412.5, 442.5, 490, 510, 560, 620, 685, 681.25, 708.75, 753.75, 760.625, 778.75, 865, 885, 900 nm @300 m basic GSD, 1200 m reduced resolution	--	--
**SCanning Imaging Absorption spectroMeter for Atmosphic CartograpHY (SCIAMACHY)/ENVISAT**	2002– 2012	Polar, LEO Sun synchronous	774	limb scanning, 500 km horizontal, 3 km vertical; Nadir scanning 960	3 d	10:00D	[214–334, 300–412, 383–628, 595–812, 773–1063, 971–1773 nm] with 1024 channels each. [310–2380 nm] with 7 bands	1934–2044, 2259–2386 nm, with 1024 bands each	--
**JAXA Greenhouse Gases Observing Satellite (GOSAT aka IBUKI)**	2009– 2024	Polar, LEO Sun synchronous	666	1150	1–3 d	10:00D	TANSO-CAI 380, 674, 870 nm @ 0.5 km; TANSO-FTS, 775–757 nm @ 10.5 km	TANSO-CAI 1600 nm 15 1.5 km; Tanso-FTS: 1720–1560 nm @ 10.5 km	TANSO—FTS, 14.28–5.55 μm, @ 10.5 km
**JAXA GOSAT-2 (IBUKI-2)**	2018– 2024 nadir, limb, and solar/lunar occultation	Polar, LEO Sun synchronous	613	790	3 d	06:00D	TANSO/CAI-2, 343 nm Fore, 380 nm Aft, 443 nm Fore, 550 nm Aft, 674 nm Fore & Aft, 869 nm Fore & Aft, 0.5 km GSD; TANSO/FTS/2 772–753 nm @10.5 km	TANSO/CAI-2 1630 nm Fore & Aft @ 1.5 km; TANSO-FTS/2 1560–1690 nm, 1920–2380 nm @ 10.5 km	TANSO-FTS/2 5.6–8.4 μm and 8.4–14.3 μm @ 10.5 km
**Landsat-7 ETM+**	1999–2022	Polar, LEO Sun synchronous	705	185	16 d	10:00	500–900 nm PAN @15 m GSD; 480, 560, 660, 830 nm @ 30 m GSD	1650, 2200 nm, 30 m GSD	11.45 μm @ 60 m GSD

**Table 5:** NASA satellites from the early 2000s: Hyperion and ALI flown on the technology demonstration New Millennium Program Earth Observer-1; MODIS and CERES were flown on both Terra and Aqua. Technical descriptions are from OSCAR, NASA, and instrument websites.

Satellite/ Instrument	Launch Date	Orbit Type	Altitude/ Swath km	Repeat Frequency, Days	Equatorial Crossing Time	UV, VNIR, 400–1500 nm; GSD m	SWIR, 1500–2500 nm, GSD m	Mid IR 3.0–6.0 μm, Thermal IR, 11–15 μm
**NMP Hyperion/EO-1**	2001– 2017	Polar, LEO Sun synchronous	691/7.7	yearly	9:45D	357–1000, 900–1600 nm @10 nm/band = 120 bands @ 30 m GSD	1600–2576 nm @ 10 nm/band = 95 bands @30 m GSD	--
**NMP ALI/EO-1**	2001– 2007	Polar, LEO Sun synchronous	691/37	80 d	09:45D	480–690 PAN @ 10 m GSD, 443, 482, 565, 660, 790, 867, 1250 nm @ 30 m GSD	1650, 2215 nm @ 30 m GSD	--
**MODIS/Terra & Aqua**	2000– 2027	Polar, LEO Sun synchronous	705/2230	1, 2 d	10:30D 10:30A	645, 858 nm @ 250 m GSD, 469, 555, 1240 nm @ 500 m GSD, 412, 443, 488, 531, 551, 667, 678, 748, 870, 905, 936, 940, 1375 nm @ 1 km GSD	--	3.750, 3.959, 4.050, 4.515, 6.715 μm @ 1 km GSD; 11.030, 12.020, 13.335, 13.635, 13.935, 14.235 μm @ 1l, GSD
**CERES/Terra & Aqua**	2000– 2022	Polar, LEO Sun synchronous	705/60	16 d	10:30D	560, 660, 810 nm @ 15 m GSD,	2165, 2205, 22,690, 2339, 2395 nm @ 30 m GSD	8.30, 8.65, 9.10, 10, 60, 11.30 μm @ 90 m GSD

**Table 6:** MODIS, ASTER and MISR were the imagers on Terra, a 5-instrument NASA Earth Observation system (EOS) facilities platform; Aqua, a 6 instrument NASA EOS facilities platform also included AIRS, a multiband VNIR and thermal imager; and Aura, a 5-instrument NASA EOS facilities platform that included the TES instruments measuring atmospheric chemistry in the lower troposphere. Data from OSCAR, NASA, and instrument websites.

Satellite /Instrument	Launch Date	Orbit Type	Altitude/ Swath km	Repeat Frequency, Days	Equatorial Crossing Time	UV, VNIR, 400–1500 nm; GSD m	SWIR 1500–2500 nm, GSD m	Mid IR 3.0–6.0 μm, Thermal IR, 8–12 μm
**ASTER/Terra**	2000–2022	Polar, LEO Sun synchronous	705/60	16 d	10:30D	3 bands, 560, 660, 810 nm @ 15 m GSD,	4 bands, 2165, 2205, 2339, 2395 nm @ 30 m GSD	5 bands, 8.30, 8.65, 9.10, 10,60, 11.30 μm @ 90 m GSD
**MISR/Terra**	2000–2027	Polar, LEO Sun synchronous	705/380	9 d, daylight	10:30D	4 bands, 446.4, 557.5, 671.7, 866.4 nm, @ 9 view angles ±26.1°, ±45.6°, ±60.0°, ±70.5° nadir @ 250 m GSD off-nadir @ 275 m GSD	--	--
**AIRS/Aqua**	2002–2026	Polar, LEO Sun synchronous	705/1650	daily	10:30A	4 bands, 425, 630, 715, 815 nm @ 2.3 km GSD	--	3 bands, 4.175, 7.21, 12.1 μm @13.5 km GSD
**TES-Nadir, TES-** **limb on Aura**	2004–2025	Polar, LEO Sun synchronous	705/885 (nadir), effective resolution 300 (limb)	nadir 16 d, limb 3 d	13:40A	--	--	4 bands of 43,750 channels nadir, 4 bands 162, 162 channels limb 11.11–15.38, 8.70–12.20, 5.13–9.09, 3.28–5.26 μm @ 0.53 × 0.53 km nadir, @ 2.3 km limb, lowest altitude

**Table 7:** This group of satellites represent an expansion in radar and microwave measurement capability, approximately during the second decade of the 2000s that include gravity measurements using dual-frequency radar with high precision accelerometers and GPS locations (GRACE, GRACE-FO), multiband radar polarimeters measuring components of the water cycle: ocean salinity (Aquarius), soil moisture (SMOS, SMAP). Technical descriptions from the OSCAR, EO-Portal, ESA Earth Online, and NASA instrument sites.

Satellite/ Instrument	Launch Date	Orbit Type	Altitude, km	Swath, km	Repeat Frequency, Days	Equatorial Crossing Time	Radar and Microwave, Type(s), Frequency(s), GSD, m
**Gravity Recovery and Climate Experiment (GRACE) NASA/DLR**	2002–2017	89° drifting orbit	485	--	--	varies	High Accuracy Inter-Satellite Ranging System, HAIRS, K-band 24 GHz ~1 cm and Ka-band 32 GHz ~9 mm. SuperSTAR Accelerometers. Blackjack GPS system.
**Gravity Recovery and Climate Experiment Follow-On (GRACE-FO) NASA/DLR**	2018– 2026	89° drifting orbit	490	--	gridded geolocated monthly, and time averaged	varies	High Accuracy Inter-satellite Ranging System, HAIRS, K-band 24 GHz ~1 cm and Ka-band 32 GHz ~9 mm. SuperSTAR Accelerometers. Tri GPS (GPS, Galileo, and GLONASS)
**Aquarius/SAC-D**, Joint NASA/Argentina, ocean salinity	2011– 2015	Polar, LEO Sun synchronous	657	390	7d	18:00 on ascending track	3 L-band radiometers and 1 L-band scatterometer, operating at 1.412 and 1.2 GHz, respectively, @ 150 km
**NASA Soil Moisture Active/Passive** (SMAP)	2015– 2026	Polar, LEO Sun synchronous	685	1000	8 d repeating ground track	06:00D	1.41 GHz, L-band. microwave radiometer @ 40 km GSD, co-aligned with SAR 1.26 GHZ full Pol HH, VV, HV, VH, @ 30 km unprocessed, 3 m km processed; 6 m antenna
**Surface Water and Ocean Topography (SWOT) CNES and NASA**	2022– 2026	78° Drifting orbit	891	120	90% globe w/2x sampling every 21 d	varies	KaRIN is Ka (35.5 GHz) radar altimeter, w/2 antennas @ 50 m horizontal GSD on land, @1km on ocean. Microwave altimeter provides water vapor correction in 3 frequencies at 18.7, 23.8, 34.6 GHz.
**Global Precipitation Measurement Constellation, NASA and JAXA. Core Observatory**	2014– 2030	62° Drifting orbit	398 km; raised to 442 km on 7–8 November 2023	245 km@13.6 GHz and 125 km @35.5 GHz	Near global 5 days/latitudes > ±65° not covered; FMI near global 2 days, latitudes > ±70° not covered	varies	Dual frequency Precipitation Radar (DPR) Ku band 13.6 GHz and Ka band 35.55 GHz; GPM Microwave Imager (GMI) 13 bands 10.65, 18.7, 23.8, 36.5, 89, 166.5, 183.31 ± 7, 183.31 ± 3 GHz with vertical polarization; 5 bands 10.65, 18.7, 36.5, 89.0, 166.5 GHz with horizontal polarization.

**Table 8:** High-spatial-resolution vertical height measurements from the first lidars in space (ICESat, ICESAT-2), and updated satellites for atmospheric chemistry, the OCO-2 and Sentinel-5 P instruments. The technical descriptions from OSCAR, EO-Portal, ESA Earth Online, and NASA instrument sites.

Satellite/ Instrument	Launch Date	Orbit Type	Altitude, km	Swath, km	Repeat Frequency, Days	Equatorial Crossing Time	UV 270–400 nm, VNIR 400–1500 nm, SWIR 1500–2500 nm	Radar and Microwave, Type(s), Frequency(s), GSD, m
**NASA GLAS/** **ICESat (LiDAR)**	2003–2010	94° drifting	600	--	91 d	varies	2 bands 532, 1064 nm Lidar @ 66 m GSD samples at 170 m intervals and 10 cm vertical, surface to cloud top at 200 m.	--
**NASA ATLAS/** **ICESAT-2** **(LIDAR)**	2018–2026	94° drifting	481–495	--	183d	varies	1 band, 1064 nm lidar @ 66 m GSD horizontal samples at 170 m intervals and 10 cm vertical surface to 200 m cloud top	--
**Orbiting Carbon** **Observatory-2 (OCO-2)**	2014–2026	Polar, LEO Sun synchronous	705	x-track 10 km, 3 pointing modes	Global, 1 month	13:30A	--	3 bands with 1024 channels, 1594–1619 nm with 0.08 nm resolution, 2024–2082 nm with 0.10 nm resolution @1.29 km X-track × 2.25 km along-track
**Sentinel 5 Precursor (5P); Tropospheric Monitoring Instrument (TROPOMI)** (Trails VIIRS on Suomi NPP by 3.5 min.)	2017–2025; free flyer	Polar, LEO Sun synchronous	824	2600	<1 d	13:30A	TROPOMI 5 Hyperspectral regions UV1270–300 nm, UV2 300–400 nm with 0.39 nm resolution, VIS 400–500 nm with 0.58 nm resolution, NIR 700–775 nm with 1.03 nm resolution, SWIR3 2305–2385 nm with 2.01 nm resolution, 7 km GSD, relaxed to 50 km for wavelengths <300 nm	--

**Table 9:** The ESA’s new exploratory research missions: Earth Explorer Satellites (#1–5) launched between 2009 and 2018. GOCE and Aeolius have completed their missions and SMOS, Cryosat, and the SWARM satellites are still operating. Data from OSCAR, EO Portal, ESA, Copernicus, and mission websites.

Satellite/ Instrument	Launch Date	Orbit Type	Altitude, km	Swath, km	Repeat Frequency Days	Equatorial Crossing Time	UV, VNIR, 400–1500 nm; GSD m	Radar and Microwave, Band Type(s), Frequency(s), GSD, m
ESA Earth Explorer #1 Gravity Field and Stead-State Ocean Circulation (GOCE)	2009–2023	Polar, LEO Sun synchronous	230	--	--	6:00A	--	3-AXIS Electrostatic Gravity Gradiometer (EGG) with 6 accelerometers, Laser Retro-Reflector (LRR), Lagrange receiver Satellite-to Satellite Tracking System (SSTI), capable of tracking 12 GPS satellites for accurate positioning.
ESA Earth Explorer # 5 Aeolius with ALADIN (Atmsopheric Laser Doppler Instrument)	2018–2023	Polar, LEO Sun synchronous	320	87	weekly	06:00D	Aladin, 325 nm laser transmitter looking 35° off nadir. Pulsed laser source 50 Hz averaging over 87 km along track. High spectral resolution lidar (HSRL) @15 m GSD. 2 detectors discriminate Mei and Rayleigh winds	--
ESA Earth Explorer # 2, Soil Moisture and Ocean Salinity (SMOS)	2009, 2025	polar, LEO, sun-synchronous	758	1050	global soil moisture 3d; salinity monthly	06:00D	--	Microwave Imaging Radiometers with Aperture Synthesis (MIRAS), passive 2-D interferometric polarimetric radiometer; L-band SAR, w/several polarimetric modes; 1.413 GHz; 50 km GSD; can be degraded for salinity to 15 km grid
ESA Earth Explorer # 3, SIRAL/ Cryosat-2	2010–2028	LEO drifting, 92°	717	--	369d, binned latitudes, monthly	varies	--	SIRAL has a 13.575 GHZ frequency, 350 MHz bandwidth, and a pulse repetition frequency dependent on the operating mode SIRAL has along track resolution @15 km GSD, in SAR mode alongtrack resolution is250 m @ 0.45 km GSD
ESA Earth Explorer #4 SWARM constellation (A, B, C), Canada’s CASSIOPE integrated as SWARM E	A,B,C 2013–2025, 2018–2021	A,C 67.75°, B 87.75°, SWARM E 80.97°	A,C @ 462, B 511, SWARM E 670	SWARM A, B. C each had 7 identical instrument packages, Accelerometer (ACC), Absolute Scalar Magnetometer (ASM), Electric Field Instrument EFI), GPS Receiver (GPS), Laser Retro-Reflector (LRR),Star Tracker Set (STR), and Vector Field Magnetometer (VFM). CASSIOPE had 8 instruments: Imaging and Rapid-screening Ion Mass Spectrometer (IRS) Suprathermal Electron Imager (SEI), Fast Auroral Imager (DAI), Magnetic Field Instrument (MFI) Radio Receiver Instrument (RRI), GPS Attitude and POsitioning Experiment (GAP), Coherent Electromagnetic radiation (CER)				

**Table 10:** A new generation of moderate-resolution VSWIR multispectral imagers including Landsat-8 and -9, and VIIRS on three platforms. Technical details from OSCAR, USGS and NASA for Landsat.

Satellite /Instrument	Launch Date	Orbit Type	Altitude, km	Swath, km	Repeat Frequency Days	Equatorial Crossing Time	UV/VNIR, 400–1500 nm; GSD m	SWIR, GSD, m	MidWave IR 3.0–6.0 μm; Thermal IR 6.0–14.0 μm, GSD m
**Landsat 8, 9**	2013– 2024, 2021– 2031	polar, LEO, sun-synchronous	705	185	16 (8 d together)	10:11D	OLI, 1 PAN 500–680 nm @ 15 m; 5 bands (444, 480, 560, 655, 865 nm) @30 m GSD	OLI 2 SWIR bands 1610, 2200 nm @ 30 m GSD	TIRS 2 bands at 10.895, 12.519 μm @ 100 m GSD
**Visible Infrared Imaging Radiometer Suite (VIIRS)**, on NASA’s Suomi NPP	2012– 2028 Extended	polar, LEO, sun-synchronous	833	3000	1, 8, 16 d	13:25A	VIIRS I-Bands: 1 day/night band 500–900 nm @ 375 m GSD; 2 bands at 640, 865 nm @ 375 m GSD; 9 bands, 412, 445, 488, 555, 672, 746, 865, 1240, 1378 nm @ 750 m GSD)	VIIRS, I-band, 1 at 1.61 μm @ 375 m; M-bands, 2 at 1.61, 2.250 μm @ 750 m	VIIRS I-bands, 2 at 3.74, 11.45 μm @ 375 m GSD, M-bands 5 at 3.70, 4.05, 8.55, 10.763, 12.013 μm @ 750 m GSD
**VIIRS on NOAA 20, 21** (previusly JPSS-1 & JPSS-2)	2017– 2027, 2023– 2030	polar, LEO, sun-synchronous	834	3000	1, 8, 16 d	13:25A	VIIRS I-Bands, 1 day/night band 500–900 nm @ 375 m GSD; 2 bands at 640, 865 nm @ 375 m GSD); M-bands, 9 at 412. 445, 488, 555, 672, 746, 965, 1240, 1378 nm @ 750 m GSD	VIIRS, I-band, 1 at 1.61 μm @ 375 m; M-bands, 2 at 1.61, 2.250 μm @ 750 m	VIIRS I-bands, 3 at 3.74, 11.45 μm @ 375 m GSD; M-bands, 5 at 3.70, 4.05, 8.55, 10.763, 12.013 μm @ 750 m GSD

**Table 11:** The European Union’s Operational Copernicus Sentinel Satellites, launched in pairs, between 2014 and 2020. These include Sentinel-1, a SAR imager with polarimetry and interferometry to measure terrestrial topography and surface deformation patterns. Sentinel-2 is a multiband moderate-resolution general purpose imager like the mission of Landsat, but with more (and narrower) bands and higher spatial resolution, but without TIR bands. Sentinel-3 imagers monitor the Earth’s oceans, land, ice and atmosphere, and the Sentinel-6 Michael Freilich is a SAR 2-band altimeter that measures waves and sea-surface heights. Description of Sentinel-5P is included in Table 6 with other second-generation atmospheric chemistry instruments. Sentinel-4 and -5 will measure atmospheric chemistry but are not yet in orbit, with S-4 planned for GEO orbit and S-5 for polar orbit. Technical descriptions from OSCAR, EO-Portal, ESA Earth Online, and instrument sites.

Satellite/ Instrument	Launch Date	Orbit Type	Altitude, km	Swath, km	Repeat Frequency, Days	Equatorial Crossing Time	VNIR, 400–1500 nm; GSD m; SWIR 1500–2500 nm, GSD m	MidIR 3.0–6.0 μm, GSD km, TIR 6-15.0 μm, GSD, km	Radar & Microwave, bands or Frequencies, GSD, km
**Sentinel 1A, 1B, 1C, 1D Interferometric SAR**	1A 2014–2024, 1B 2016 2022 1C 2024–2031, 1D 2025–2032	polar, LEO, sun synch-ronous	693	depends on mode 80, 250, 400 km	extra wide swath 8 d, 12 d each, 180° apart	06:00D	**--**	**--**	1 SAR C band at 5.405 GHz side looking 15°–45° off nadir, Dual polarization, SAR imager 4–80 m GSD mode dependent, interferometric SAR (nSAR), 20 km GSD.
**Sentinel 2A and 2B and Sentinel 2C and 2D**	2A 2015–2024, 2B 2017–2024, 2C 2024–2031, 2D 2028–2033	polar, LEO, sun synch-ronous	786	290	10 d each, together 5 d	10:30D	Multi-spectral Instrument (MSI) 4 bands at 490, 560, 665, 842 nm @ 10 m; 4 bands at 705, 740, 783, 865 nm @ 20 m; 2 bands at 1610, 2190 nm @ 20 m GSD. Includes 3 calibration bands 443, 945, 1375, nm bands @ 60 m GSD	**--**	**--**
**Sentenel 3A and 3B, Ocean and Land Colour Instrument (OLCI) and Sea and Land Surface Temperature Radiometer (SLSTR) and Sentinel-3 radar altimeter.**	3A 2016–2026, 3B 2018– 2028	polar, LEO, sun synch-ronous	814.5	OLCI = 1270, off nadir view avoids sunglint; SLSTR nadir swath 1400 km all viewing; diurnal viewing @740 km	OLCI global 1 mo. for 30 km ave. spacing or 10d for 100 km ave. spacing. SLSTR global coverage monthly, or 10 d w/100 km ave. spacing.	10:00D	OLCI, push-broom side-to-side telescopes. 2 bands 761.25, 767.5 nm at 2.5 nm, 1 band 764.275 nm at 3.75 nm; 9 bands 412.5, 442.5, 490, 510, 620, 665, 708.25, 885, 900 nm at 10 nm, @ 300 m; 2 bands 940, 1020 nm at 40 nm @300 m GSD SLSTR, 6 bands 555, 659, 865, 1375, 1610, 2250 nm @ 500 m GSD	SLSTR, 3 bands 3.74, 10.85, 12.02 μm @ 1 km	SRAL SAR altimeter dual frequency C-band ~5 cm and 5.4 GHz and a Ku-band ~2 cm at 13.58 GHz 20 km GSD. MicroWave Radiometer 2 channels K band ~0.8 cm and 23.8 GHZ; Ka band ~0.27 cm and 36.5 GHz; nadir viewing 20 km GSD. Includes a Precise Orbit Determination package.
**Sentinel 6A and 6B, Michael Freilich, Jason Continuity of Service (Jason-CS A and Jason-CS B)**	6A 2020–2027, 6B 2025–2032, 6C 2030–2036	66° drifting orbit	1336	290	10d	**--**	**--**	**--**	POSEIDON-4, 2-band SAR altimeter, Ku ~2 cm and 13.575 GHz and C ~5 cm and 5.31 GHz; w/12 m antenna. A 3-band Multi-frequency 18.7, 23.8 and 34 GHz microwave radiometer, approximately Ku-, K, Ka bands. Advanced Microwave Radiometer for Climate, (AMR-C), with High-Resolution Microwave Radiometer (HRMR) mm-wave channels at 90, 130, 160 GHz (λ = ~31, 6.1, 3.7 μm)

**Table 12:** Landsat-8, -9 and Sentinel-2A, 2B harmonized data product. Native data characteristics had to be reconciled to create a harmonized data set with the best resolution possible, given differences in orbits, instrument characteristics. Table from Harmonized Landsat Sentinel-2 (HLS) Produce User Guide V2.0 (https://lpdaac.usgs.gov/documents/698/HLS_User_Guide_V2.pdf (accessed on 3 May 2024)).

Satellite Characteristics		Landsat 8/OLI-TRS	Sentinel 2A/MSI	Sentinel-2B/MSI
		11 February 2013	23 June 2015	7 March 2017
**Equatorial Crossing Time,** **Descending orbit**		10:00	10:30	10:30
**Spatial Resolution, GSD**		30 m OLI/100 m TIRS	10 m/20 m/60 m (for different bands)	
**Swath/Field of View**		180 km/15°	290 km/20.6°	
**Spectral bands, Central Wavelength**	Ultra blue	443 nm	443 nm (60 m)	
	Visible	482, 561, 655 nm	490 (10 m), 560 (10 m), 665 (20 m)	
	Red edge	--	705 (20 m), 740 (20 m), 783 nm (20 m)	
	NIR	865 nm	842 (10 m), 865 nm (20 m)	
	SWIR	1373 nm	1375 nm (60 m)	
	Water Vapor	--	945 nm (60 m)	
	Thermal	10.9, 12.0 μm	--	

**Table 13:** Next-generation EO Imaging Spectroscopy has arrived with two free-flying full-spectrum imaging spectroscopy missions for land observation. The Italian Space Agency (ASI) launched PRISMA in 2019, a 237-band, 2-detector imager, measuring the spectrum from 400 to 2500 nm with 30 m GSD. The German Space Agency (DLR) launched EnMAP in 2022, a 146 band, 2-detector imager measuring the spectrum from 420 to 2450 nm, with 30 m GSD. Technical descriptions from OSCAR, EO-Portal, ESA Earth Online, the PRISMA and ENMAP sites.

Satellite/ Instrument	Launch Date	Orbit Type	Altitude	Swath, km	Repeat Frequency Days	Equatorial Crossing Time	UV/VNIR, 400–1500 nm, GSD, m	SWIR, 1500-2500 nm, GSD, m
**Italian Space Agency (ASI) PRecursore IperSpettraie della Missione Applicativa (PRISMA)**	2019–2025	polar, LEO, sun- synchro- nous	615	30	29d	10:30D	66 bands 400–1010 nm at 10 nm bandwidth @ 30 m GSD	171 bands 920–2505 nm at 10 nm bandwidth @ 30 m GSD
**German Space Agency (DLR)** **Environmental Monitoring and Analysis Program (EnMAP)**	2022–2026	polar, LEO, sun- synchro- nous	653	30	27d	11:00D	92 bands 420–1000 nm; 6.5 nm ave. bandwidth @30 m GSD	48 bands SWIR 1: 900–1380 nm; 28 bands SWIR 2: 1480–1760 nm; 50 bands SWIR3: 1950–2450 nm, 10 nm ave. band-width @30 m GSD

**Table 14:** Experimental EO instruments on the International Space Station. There are three imaging spectrometers currently on the ISS: DESIS, an experimental imaging spectrometer from the DLR that covers the VNIR only, but in 235 bands with high spectral resolution (2.55 nm bands). HISUI is a full-range 185-band imaging spectrometer from a joint JAXA and METI program. EMIT, a NASA Earth Ventures Instrument (EVI-4) is a high-fidelity instrument with 285 nm bands across the 380–2500 nm VSWIR region. The OCO-3 is a NASA Earth System Pathfinder (ESSP-6) technology demonstration instrument to measure carbon in the atmosphere. The ECOSTRESS thermal imager (EVI-2) instrument is an 8-band TIR imager with high spatial resolution. Lastly, the GEDI (EVI-1) is a 1064 nm band lidar imaging system to map land cover vegetation height. Technical descriptions from OSCAR, Gunter’s page, ISS website, and NASA websites for individual missions.

Satellite/ Instrument	Launch Date	Orbit Type	Altitude km	Swath, km	Repeat Frequency, Days	VSWIR 400–2500 nm at 10 nm Resolution @ GSD	TIR 6–15.0 μm, GSD, m
**DLR Earth Sensing Imaging Spectrometer** (DESIS)	2017– 2024	51.6° drifting orbit	407	30	3 d spatial repeat, but 63 d for time of day repeat (TOD)	240 VNIR (450–915 nm) with ~1.9 nm bandwidths, @ 30 m GSD	--
**HISUI Hyperspectral imager and Multispectral imager**; Japanese Ministry of Education, Culture, Sports, Science and Technology (MEXT) and Japanese Space Agency (JAXA).	2019– 2024	51.6° drifting orbit	407	20	3 d spatial repeat, but 63d for TOD	185 bands 400–250 nm, with 10.0–12.5 nm spectral resolution, IFOV is 20 m × 30 m	--
**Earth Surface Mineral Dust Source Investigation (EMIT)**. NASA Earth Ventures Instrument (EVI-4)	2022– 2026	51.6° drifting orbit	407	75	3 d spatial repeat, but 63d for TOD	286 bands 380–2500 nm at 7.4 nm sampling, @60 m GSD	--
**NASA Orbiting Carbon Observatory (OCO3)**, ESSP-6 mission	2019– 2029	51.6° drifting orbit	407	4.5 × 4.5; Pointing 40 km both sides ISS ground track	3 d spatial repeat, but 63d for TOD	--	--
**NASA ECOsystem Spaceborne Thermal Radiometer Experiment on Space Station** (ECOSTRESS) EVI-2	2018– 2026	51.6° drifting orbit	407	384	4d repeat w/63d exact repeat for time of day	--	5 bands at 8.285 *, 8.785, 9.060 *, 10.552, 12.001 μm at 69 m × 38 m GSD * bands unavailable after 5 May 2019
**NASA/U. Maryland, Global Ecosystem Dynamics Investigation** (GEDI), EVI-2	2018– 2031, removed 3/2023–7/2024, returns until January 2030	51.6° drifting orbit	407	7	3 d spatial repeat, but 63 d for TOD	3 Nd:YAG lasers emitting at 1064 nm light, split into 7 beams, dithered to produce 14 ground track spot beams, 25 m footprints, spaced 500 m x-track and 60 m along track. Pointing strategy for targets.	--

**Table 15:** The technical characteristics of currently flying and next-generation non-governmental (commercial, NGO) satellites. Data from these satellites are widely used, especially for small pixel sizes of sub-meter to a few meters that are highly desired by environmental remote sensing data users. There are public archives for some of these data, especially older data. Some data can be acquired at no cost if you agree to various non-commercial limitations on the data. Technical descriptions from OSCAR, Gunter’s page, and, and from individual mission websites.

Satellite/ Instrument	Launch Date	Orbit Type	Altitude km	Swath, km	Repeat Frequency Days	Equatorial Crossing Time	Panchromatic Resolution, GSD m	VNIR, Resolution, 400–1500 nm, GSD m	Radar, Microwave, m GSD
**Satellite pour** **l’Observation de la Terre (SPOT), 6,7**	2012, 2014, 10 year life	Polar LEO sun synchro-nous	679	60	26 d each, with pair 13 d	10:00D	1 BAND 450–745 nm @1.5 m GSD	4 bands 485, 560, 660, 825 nm @ 2.0 m GSD	--
**Pléiades 1A and 1B (CNES)**	2011–2024, 2012–2024	Polar LEO sun synchronous	694	20, at NADIR	26d each, with pair 13 d	10:00D	1 band at 480–930 nm @ 50 cm GSD	4 bands 490, 550, 660, 850 nm @ 2.0 m GSD	--
**Pléiades NEO, 4 satellites at 90° (CNES)**	2020 (pair 2), 2022 (pair 2)	PolarLEO sun synchronous	620	14, at NADIR	4 w/6.5 day repeat	10:30D	1 band at 450–800 nm @ 30 cm GSD	6 bands at 425, 485, 560, 655, 725, 825 nm @ 1.2 m GSD	--
**TanDEM-X (DLR, data provided by Astrium/Infoterra)**	2010–2026	Polar LEO sun synchronous	505	10–100, mode dependent		06:00D	--	--	x-band SAR (9.65 GHz) @ 1–16 m GSD mode dependent Multipolarization
**PlanetScope Constellation of 200+ Dove Cubesats, 3-band sensors PS-2 and 4-band PS2.SD.**	2014-	Polar LEO sun synchronous	475–525	24 × 8 km or 24 × 16 km scene based	daily, at Nadir, global	9:30–11:30D	--	Originally 3-band RGB satellites, now 4 band B, G, R, NIR, @ 3.0–4.1 m GSD	--
**PlanetScope Constellation of ~195** **SuperDove satellites. called SPB.SD** **imagers**	2018-	Polar LEO sun synchronous	504	32.5 × 19.6 km scene based	~daily at Nadir, global	9:30–11:30D	--	8 bands, B (443, 490), G (531, 565), Y 610, R 665, Red- edge 705, NIR 865 @ 3.7 m GSD	--
**PlanetScope Constellation of 21** **SkySats**	2014–2025	Polar LEO sun synchronous	450	Sats 1,2 8 km, Sats 3 15 are 5.8 km, Sats 16 21 are 20.5 km × 5.9, all at Nadir	4–5 day individual revisit; Constellation ave. 6–7 images/day global	10:30D (Sats 1–6, 14–16) 13:00A (Sats 1, 2, 8–13)	1 pan 400–900 nm @ 0.5 m GSD	4 VNIR bands at 482.5, 555, 650, 820 @ 2.0 m GSD	--
**PlanetScope RapidEye-4; a 5-satellite constellation, originally launched in 2008**	2008- Planet acquired in 2015, retired 2020	Polar LEO sun synchronous	630	77	daily with pointing; 5.5 d at nadir over mid latitudes	11:00 + 1 h D	--	Multispectral Imager (MSI) 5 VNIR bands at 465, 555, 660, 710, 820, at 6.5 m GSD st Nadir, resampled to 5 m on ortho products	1 pan 400–900 nm @ 0.5 m GSD

**Table 16:** Geostationary Satellites Currently in Orbit. Modern GEO measure the full disk of the Earth as the largest area covered. Japanese AHI and NOAA ABI imagers have 16 bands from visible through the thermal infrared; only 1 band of which is different. The EUMETSAT Flexible Combined Imager (FCI) on the MTG-1 satellite acquires similar spectral and spatial measurements. Both GOES and MTG l1 have a lightning strike imager (GLM and LI) on their platforms (along with other instruments). Technical descriptions for all instruments are found OSCAR, EO Portal, Himawari at NASA Earth Data, GOES at NOAA, and MTG l1 at EUMETSAT.

Satellite/ Instrument	Launch Date	Orbit Type	Altitude km	Swath, km	Acquisition Periods	VNIR, SWIR 400– 1500 nm, GSD, m	Mid IR 3.0–6.0 μm TIR, 6–15.0 μm, GSD, km
**JAXA Himawari 8 and 9 3rd Generation, contains Advanced Himawari imager (HMI) and a data collection service.**	2014– 2030, 2016– 2030	Japan, western Pacific, 140.7°	35,786	Full disk and regional	Multi modes, 2.5 min to 10 min full disk	Advanced Himawari Imager (AHI) 1 VIS Red at 645 nm @0.5 km, 3 VNIR 455, 510, 860 nm @ 1 km GSD, 2 SWIR 1610, 2260 nm @ 2 km GSD	10 AHI 3.85, 6.25, 6.95, 7.35, 8.60, 9.63, 10.45, 11.20, 12.35, 13.30 μm @ 2 km GSD. Data transfer uses Ka band (18.1–18.4 GHz)
**NOAA GOES 16 (GOES-** **East), 17 (GOES West), 18 (GOES** **West, replaced GOES** **17) Advanced Baseline Imager (ABI), Geostationary** **Lightning Mapper (GLM)**	2016– 2030, 2017– 2032, 2022– 2033	GOES, 16 over Eastern US (75.2° W), GOES 2017/2018 over western US (137.0° W)	35,786	Full disk and regional	multi-modes from 30 s to 15 min	ABI VIS 1 band 640 nm @ 0.5 km, 3 VNIR bands 470, 865, 1378 nm @ 1k GSD, 2 SWIR bands 1610, 2250 nm @ 2 km GSD. GLM measures flash intensity against threshold background, optical energy over 2 × 2 km grid cell. Units of Average Flash Area in km^2^ and Total Optical Energy per grid cell per time period (5 min) in femtojoules (fJ, 10^−15^ J)	ABI 10 bands, 3.90, 6.19, 6.90, 7.3, 8.4, 9.6, 10.3, 11.2, 12.3, 13.3 μm @ 2 km GSD
**EUMETSAT METOP** **Third Generation, MTG Flexible Combined** **Imager (FCI) on MTG-I1, MTG-I2. Lightning Imager (LI) on MTG-I1, MTG I2.**	MTG-I 1 2022– 2030, MTG- I 2 2026– 2036	GEO 0° Latitude	37,786	Full disc and regional	Multi modes, 2.5 min to 10 min full disk	Flexible Combined Imager (FCI), 2 channels 640, 2200 nm @ 500 m GSD and 6 bands 444, 510, 640, 865, 914, 1380, 1610, 2250 nm @ 1 km GSD. Lightning Imager (LI), measures pulse intensity over a narrow band at 777.4 nm with a 4.5 km GSD	FCI, 2 bands at 3.8, 10.50 @ 1 km GSD in hi res mode. and 8 bands 3.8, 6.30, 7.35, 8.70, 9.66, 10.50, 12.30, 13.3 μm @ 2.0 km GSD
**EUMETSAT METOP** **Third Generation, MTG-S) Sounder. the Infrared** **Sounder (IRS) and Sentinel 4 UVN will be flown on the MTG-S1.**	MTG-S 1 2025– 2035; MTG-S 2 ~10 years after launch of S 1	GEO over 0° Latitude	75,779	Full disc and regional	Multi modes, 2.5 to 10 min for full disk	--	IRS Fourier transform Interferometer Sounder w/large detector arrays, 4.44–6.25 μm and 8.26–14.70 μm at 0.604 cm^−1^ wave number resolution, @ 4 km GSD
**Sentinel 4A, 4B, Ultraviolet, Visible, Near-Infrared Imager (UVN) Flown on the MTG-S1 with the Infrared Sounder (IRS)**	A 2025–2032 B launch~ 2034	GEO, 30–65° N latitude, 30° W–45° E longitude.	37,786	60 min cycle	--	UVN. 190 bands Ultraviolet 305–400 nm, 200 bands Visible 400–500 nm @ 0.5 nm wavelength resolution; 21 bands NIR 750–775 nm at 1.2 nm wavelength resolution @ 8 × 8 km GSD	--

**Table 17:** Three new instrument technologies. The PACE mission, launched in February 2024, will monitor the global biosphere of the oceans, with its imaging spectrometer, the Ocean Color Instrument (OCI). It is assisted by the SPEXone polarimeter and the HARP-2 Hyper-Angular Research Polarimeter. TRISHNA (2025 launch) is a multiband thermal imager to measure surface temperature and evapotranspiration. It will be a push-broom imager with high-spatial-resolution SWIR and TIR bands. NISAR is an R&D satellite (2024 launch) to provide more detailed global hydrology, soil moisture, and biomass estimation. It has two SAR frequency instruments, SAR-L and SAR-S, both with multiple polarization modes and variable spatial resolution to provide high-resolution all-weather imagery of ocean and land surfaces, including soil moisture. Technical descriptions from OSCAR, EO-Portal, NASA, and instrument sites.

Satellite/ Instrument	Launch Date	Orbit Type	Altitude	Swath, km	Repeat Frequency Days	Equatorial Crossing Time	UV/VNIR/SWIR, 400–2500 nm, GSD; an TIR 6.0–12.0 μm @GSD in km	Radar and Microwave Bands, Wavelengths, Frequencies
**PACE Ocean Color Instrument (OCI) and 2 polarimeters (SPEXone and HARP2)**	2024–2027	polar, LEO, sun-synchron ous	676	**OCI** = 2663 at 20° Tilt to avoid sunglint, SPEXone = 100 km, HARP2 = 1556 km	OCI = 1–2 d; SPEXone = 30 d, Harp2 = 2 d	12:00D,	OCI 46 bands from 342.5–887 nm at 5 nm resolution, 7 bands NIR-SWIR bands 940, 1038, 1250, 1378,1615, 2130, 2260 nm @ 1 km GSD. SPEXone is a multiangle polarimeter, measures intensity angle and polarization linearity, 385–770 nm in 2–4 nm steps (yielding about 150 bands) at 5 view angles (−57°,−20°, 0°, 20°, 57°) @ 2.5 km^2^ GSD; also bands in same range as the 7 OCI bands but in 15–45nm steps yielding about 30–88 bands; HARP-2 has 4 bands 441, 549, 669, 873 nm with bandwidths of 15, 12, 16, 43 nm with 10 view angles at 440, 550, 870 nm at 3 angles 0, 45°, 90° at 2.6 km GSD.	--
**Carbon Mapper Tanager satellites from Planet**	first 2 Tanager satellites, 2024; Phase 2 expansion 2025; 5 yrs	Polar, LEO Sun Sychro-nous	405	18 km, measured in 1200 km wide-strips	TBD	TBD	Pushbroom VSWIR imaging spectrometer 300–2500 nm with 5 nm spectral sampling @30 m GSD	--
**MethaneSat Environmental Defense Fund and New Zealand Space Agency**	launched 3/4/2024, 5 yrs	51.6° Drifting Orbit	590	200	3–4 days global	TBD	VSWIR 2 HgCdTe detectors, #1 with 2 bands having 28 channels between 1249–1305 nm and 42 channels between 1598–1683nm w/0.2 nm resolution and 0.6nm spectral sampling @ 100 × 400 m GSD. SNR 190. #2 VSWIR HgCdTe detectors: 400 channels from 1583–1683 nm with 0.25 nm resolution and 0.08 nm sampling. @100 × 400 m GSD and SNR 190.	--
**Thermal infraRed Imaging Satellite for High resolution Natural resource Assessment (TRISHNA) French CNES/** **Indian ISRO Space Agencies.**	2026–2031	polar, LEO, sun-synchro-nous	761	932	3 d	12:30D	4 bands (485, 555, 670, 860, 1380, 1650 nm) @ 57 m GSD and 4 bands TIR 4 bands, (8.6, 9.1, 10.4, 11.6 µm) @ 57 m GSD	--
**NASA-Indian Space Research Oeganization (NASA ISRO) joint NISAR Mission**	2024–2026	polar, LEO, sun-synchro-nous	747	242	12 A and D (6 day for both)	06:00D 18:00A	--	Dual frequency SAR multi-polarimetric modes, GSD mode dependent 2–7 m. Side looking 33–47°. S-band 3.162–3.237 GHz (~9 cm) and 3–24 m GSD; and L band 1.215–1.3 GHz (24 cm) and 3–48 m GSD.

**Table 18:** The METOP Second-Generation (SG) series of polar weather satellites will have two platforms (1A and 1B). The SG-1A has two imagers, METImage and 3MI, and a temperature profiling sounder, IASI-NG. Sentinel-5 will fly on this platform (Sentinel-5P, still flying, is described in Table 5), and measures atmospheric chemistry with the UVN sounder. The SG-1B carries the Ice Cloud Imager (ICI) with radar at mm and sub-mm frequencies, and a MicroWave Imager (MWI) to measure precipitation and humidity soundings. Older NOAA polar platforms are described in Table 2 and the current NOAA-20 and -21 platforms are in Table 7. Technical descriptions from OSCAR, EO-Portal, ESA Earth Online, and EUMETSAT.

Satellite /Instrument	Launch Date	Orbit Type	Altitude	Swath km	Repeat Frequency Days	Equatorial crossing Time	VNIR/SWIR, 400 2500 nm, GSD, km	MIDIR 3.0–6.0 μm, TIR 6.0–14.0 μm, @ km GSD	Radar and Microwave Bands, Wavelengths, Frequencies, GSD km
METOP-SG-Polar Weather Satellite 1A, 2A	2025–2033, 2032–2040	Polar, LEO, Sun-synchro-nous	835	METImage 2670 km, 3MI 2200 km, IASI-NG 2000 km, S-5 2715 km, MWS 2300 km	3MI D in sunlight; MET- Image, D, VSWIR, 2x D TIR. MWS near 2 x D	09:30D	3MI: 9 channels w/polarization 410, 443, 490, 555, 670, 865, 1370, 1650, 2130 nm, 3 channels no pol: 763, 765, 910 nm @ 4 km GSD. METImage 11 VNIR/SWIR Channels 443, 555, 668, 751.5, 762.7, 865, 914, 1240, 1375, 1630, 2250 nm @0.5 km	METImage 9 bands: 3.74, 3.959, 4.05, 6.725, 7.325, 8.54, 10.69, 12.02, 13.345 μm @ 0.5 km; IASI-NG sounder w/16,921 channels 4.62–15.50 μm @4 × 412 km spaced GSD, within 100 × 100 km^2^ grid.	MWS 24 channels, single pol (V or H), at frequencies: 54.4, 54.94, 55.5, 57.290344 ± 0.3222 + 0.022, 57.290344 ± 0.3222 ± 0.010, 57.290344 ± 0.3222 ± 0.0045, 89, 164–167, 183.311 ± 7.0, 183.311 ± 4.5, 183.311 ± 3.0, 183.311 ± 1.8, 183.311 + 1.0, 229.0 GHz@ GSD 17 GSD channels 89–229 GHZ, 20 km for channels 50–59 GHZ, 40 km for channels 23.8–31.4 GHz.
METOP-SG-Polar Weather Satellite 1B, 2B	2026–2034, 2033–2041	Polar, LEO, Sun- synchro-nous	835	ICI, MWI = 1700 km, SCA 2 swaths @ 660 km separated by 525 km gap. 3 looks at 45, 90, 135°. MWI = 1700 km swath	ICI, MWI D, SCA ~1.5 D	09:30D	--	--	ICI 11 frequency channels 183.31 ± 7.0, 183.31 ± 3.4, 183.31 ± 2.0, 243.2 ± 2.5, +325.15 + 9.5, 325.15 ± 3.5, 325.15 + 1.5, 448 ± 7.2, 448 ± 3.0, 448 ± 1.4, 664 ± 4.2 GHz.; SCA C-band, 5.355 GHZ side looking, 25 km sampling at 12.5 km intervals, hi res mode 15–20 km w/6.25 km sampling intervals all @ 15 km GSD; MWI 18 frequencies 26 channels, 18.7, 23.8, 31.4, 50.3, 52.7, 53.24, 53.75, 89, 118.7503 ± 3.2, 118.7503 ± 2.1, 118.7503 ± 1.4, 118.7503 ± 1.2, 165.5 ± 0.725, 183.31 ± 7.0, 183.31 ± 6.1, 183.31 ± 4.9, 183.31 ± 3.4, 183.31 ± 2.0 GHz, GSD wavelength dependent
Sentinel-5 flown on METOP-SG-1A, SG-2A	2025–2033, 2932–2040	Polar, LEO, Sun-synch-ronous	835	2715	daily	09:30D	UVNS: 7 channels, 30 bands from 270–300 nm @ 1 nm sampling, 140 bands from 300–370 nm @0.5 nm sampling, 260 bands from 370–500 nm @0.5 nm sampling, 6 bands from 685–710 nm @ 0.4 nm sampling, 7 bands from 745–773 nm @ 0.4 nm sampling, 340 bands from 1590–1675 nm @ 0.25 nm sampling, 320 bands from 2305–2385 nm @ 0.25 nm sampling. @ 7 km GSD, degraded to 28 km for 270–300 nm.	--	--

**Table 19:** The ESA Earth Explorers, numbers: −6, −7, −8 are expected to launch between 2024 and 2026, all three into LEO sun-synchronous orbits. The **EarthCARE** (EE #6) is composed of three instruments, a multispectral imager, a Broad-Band (microwave) Radiometer, and a Cloud Profiling Radar to measure vertical distribution of water and ice. **Biomass** (EE #7) will carry the first P-band radar in space, a quad polarized Interferometric SAR (433 MHz, and ~70 cm). It will measure aboveground forest canopy biomass densities and distributions. **FLEX** (EE #8) is designed to capture solar induced florescence (SIF) with its FLORIS instrument, and will fly in tandem with Sentinel-3D, to utilize its measurements in fluorescence computations, and to provide context across landscapes. Technical descriptions from OSCAR, EO-Portal, ESA Earth Online, and instrument sites.

Satellite/ Instrument	Launch Date	Orbit Type	Altitude	Swath, km	Repeat Frequency Days	Equatorial crossing Time	UV/VNIR, 400–1500 nm, GSD m	TIR GSD μm	Radar and Micro- Wave, Bands Wavelengths, Frequencies
**ESA Earth Explorer Missions: #6 Earth Cloud, Aerosol, and Radiation Mission (EarthCARE)**	2024– 2027, 3.5 year life	polar, LEO, sun-synch-ronous	394	MSI = 150 BBR = 3 view angle, 10 km each	25 d	14:00D	Multi-Spectral Imager (MSI) 4 bands 670, 865 nm, 1.67, 2.21 μm @ 500 m; ATLID Measures 1 band at 354.8 nm	MSI TIR 8.8, 10.8, 12.0 μm @ 500 m	Broad-Band Radiometer (BBR) 2 channels, 0.25–4.0,0.25–50 μm at 3 view angles (+ 50° Fore, AFT and nadir) with 10 km footprint @ 1 km GSD; CPR 1 band at 94.05 GHz at@ 500 m.
**BIOMASS**	2025, 5- year life	polar, LEO, sun-synch-ronous	660	50–30 km	25 d	06:00D	--	--	SAR P-Band ~70 cm wavelength and 435 MHz; SAR, Quad polarized Interferometric. 12 M antenna; side looking incidence angle 25° @50–60 m GSD.
**ESA Earth Explorer # 8, Fluorescence Explorer (FLEX)** flys in tandem w/Sentinel-3	2026, 3.5 year life	polar, LEO, sun-synch-ronous	800	150	27 d	10:00D	FLORIS 3 bands with 2800 channels from 500–780, 110 channels from 686–697, and 10 channels from 759–769 at 0.1 nm, and bands with 750–1500 channels from 500–2000, sampled at 1–2 nm resolution; all @ 300 m	--	--
**ESA Earth Explorer #10, Harmony**, 1A and 1B, flys in formation with Sentinel-1, 350 km front and behind. Definition stage.	2029– 2034	98.2° polar sun Synchro-nous	693	TBD	27 d	06:00D	--	Muiltiview VIS/TIR imager TBD	receive only, C-band SAR from Sentinel 1.

**Table 20:** NASEM Decadal Survey of 2017. The NASEM report to NASA identified five missions as highest priority for the Earth Science Directorate. The original missions were Surface Biology and Geology (SBG), now SBG-TIR and SBG-VSWIR instruments. Mass change (MC), a follow-on mission to GRACE-FO, and the surface deformation and change has been held in various planning exercises. The fourth mission was to support aerosol research and monitoring and the fifth to improve understanding of precipitation, convection, and clouds. These were grouped together into one Atmosphere-Observing System (AOS) mission that now has planned instruments in two orbits, polar and inclined. The AOS Sky and HAWCSat platforms will be in the polar orbit (2031 launch) and the AOS STORM and PMM will fly in an inclined orbit (2029 launch), each with multiple instruments. The original descriptions came from the 2017 Decadal Survey and current program plans. Technical descriptions from 2017 DS, OSCAR WMO, NASA, instrument sites.

Satellite /Instrument	Original NASEM Recommendation	Launch Date	Orbit Type	Alttude, km	Swath, km	Repeat Frequency, Days	Equatorial crossing Time	VNIR, SWIR, 400–1500 nm, 1500–2500 nm, GSD m	TIR 3.0–13.0 μm, GSD m
**NASA Surface Biology and Geology** (SBGTIR)	Hyperspectral imagery in the visible and shortwave infrared and Multiband or Hyperspectral imagery in the Thermal IR	2029, 3 year life	polar, LEO, sun synchro- nous	665	VNIR camera 630. TIR TBD up to 935	2–3 d	1:30D	Visible Near-Infrared VNIR 2-band camera from ASI (665, 835), @ 30 m; 1 SWIR band at 1.65 μm @ 60 m	**TIR** 8 bands TBD, between 3.0–12 μm, at <60 m GSD
**NASA Surface Biology and Geology (SBG-** VSWIR)		delayed, 2029– 2032	polar, LEO, sun synchro- nous	623	185	16 d	11:00D	VNIR 52 bands 380–900, 10 nm sampling @ 30 m GSD, SWIR 1605 bands from 900–2500 nm @ 30 m GSD	**--**
**Mass Change and Geosciences International Constellation (MAGIC-1, MAGIC-2)** NASA & ESA Joint venture	“Either of two ranging techniques (microwave and optical) that are being evaluated as part of mission planning. GRACE-FO uses a two frequency (24 GHz) and Ka-(32 GHz) high accuracy inter-satellite ranging system and Tri-G GPS (GPS, Galielieo, GLONASS) system.	2029 first pair, second TBD	70° Drifting orbit	397	**--**	**--**	**--**	MicroSTAR, a 3 axis accelerometer to correct non-gravitational acceleration error along 3 ultra sensitive axes. Laser Tracking Instrument (LTI) with 4-corner cube reflectors with lasers at 1064 nm wavelength for tracking the other satellite in pair.	**--**
**Surface Deformation and Change**	Interferometric Synthetic Aperture Radar, (InSAR) with ionospheric correction	TBD program still in Pre Phase A	**--**	**--**	**--**	**--**	**--**	**--**	**--**
**Atmosphere Observing System (AOS) AOS-Sky**	Aerosols: Backscatter lidar and multichannel multiangle/polarization imaging radiometer flown together; Clouds, Convection and Precipitation: Dual-frequency radar, multifrequency passive microwave and sub-mm radiometer	2031	2 satellite polar sun synchronous orbit; 2 in drifting 55° inclined orbits	450	AOS-sky MWR 750 km, SKY Multiangle Polarimeter 300 km, FIR Imaging Radiometer 100 km. AOS HAWCSat ALI 200 km limb scanner. SHOW (SAWCSat) 63 km. TICFIRE 640 km w/30 km vertical.	ALI (SAWCSat) 24 d; SHOW (SAWCSat) global 2 d; FIR Imaging Radiometer global 2 d;	AOS-Sky 13:30D	AOS-Sky Lidar range 532 nm and 1064 nm, polarization sensitive; w/30 m vertical resolution, 350 m footprint, SKY Multi-angle Polarimeter (MAP) 9 bands 380–1570 nm: 2 channels UV 350–390 nm, VIS 410–750 nm for aerosol and spectral cloud. 1350–1400 nm for cirrus clouds, 3 channels from 870–1570 nm for bispectral cloud plus Hyperangle 670–870 nm, 900–960 nm for H2Ov in bispectral clouds, at 0.5 km GSD. HAWCSat Aerosol limb Imager (ALI) 10 channels 610–1560 nm limb scanning and 0.25 km vertical resolution. HAWCSat-SHOW limb viewing imager 1362–1368.32 nm range, 0.13 km vertical resolution	AOS sky: high frequency MWR 89–113 GHz, 3-channel 183, 325 GHz, 2- channel 640–700 GHz nadir viewing @ 10 km GSD, FIR Imaging Radiometer (TICFIRE) 6 bands 7.5–50 μm (7.5–9.5, 10–12, 12–14, 27.35–29.75, 22.5–27.5, 30–50 μm), AOS-Sky Radar, nadir viewing Doppler radar Ka band 35 GHz or 94 GHz (W-band) @2 km horizontal and @500 m GSD.
**Atmosphere Observing System (AOS) AOS-Storm**		Storm 2029, HAWCsat in 2031	drifting 55° inclined orbits	430	AOS-Storm MWR 700 km, Aerosol Limb imager 200 km, Water Vapor 430 km. Limb imager 63 km. Ku AOS Doppler SAR 255 km. AOS PMM TBD.	AOS STORM MWR global 2 d. PMM MWR global 2 d. PMM Doppler Radar global 5.5 d.	various	AOS-Storm MWR (SAPHR-NG) passive microwave 89–113, 184 (6 channels), 325 (3 channels) GHz @ 3–10 km GSD; AOS-Lidar nadir viewing, 2 band 532, 1064 nm polarizationsensitive lidar @ 350 m footprint, 30 m vertical. AOS PMM TBD	AOS-Storm MWR 89, 183, 325 GHz @ 3–10 km GSD; AOS Doppler Radar (JAXA), Ku (13.6 GHz) wide-swath doppler radar @ 500 m vertical resolution and 5 km
									GSD.

**Table 21:** Six Copernicus Sentinel Expansion Missions. CO2M, will be the first, to be launched in 2025 and the others between 2029 and 2033. The CO2M, CHIME, and LSTM will fly in LEO sun-synchronous orbits. The CO2M mission has three platforms (A, B, C) to monitor global CO_2_ patterns with an integrated imaging spectrometer and a 3-band cloud Imager (CLIM). The second mission, CHIME (A, B), will have a full-spectrum imaging spectrometer to be launched into the Sentinel-2 orbit. The third mission is the LSTM (A, B), previously delayed (launch 2028) to be paired with TRISHNA and the SBG-TIR mission, will have a 12:30 orbit to obtain high-spatial-resolution multiband thermal imagery in support of the Sentinel-2, CHIME, and SBG-VSWIR missions. Sources include instrument sites, OSCAR, Copernicus Expansion missions, and EO Portal.

Satellite/ Instrument	Launch Date	Orbit Type	Altitude, km	Swath, km	Repeat Frequency Days	Equatorial crossing Time	VSWIR 400–2500 nm; TIR 7.5–13 μm GSD m or km
**Copernicus Expansion Mission, Copernicus Anthropogenic Carbon Dioxide Monitoring (CO2M)**	CO2M-A 2026–2033, CO2M-B 2027–2034, CO2M-C, 2029–2036	Polar sun synchronous	735	CO2I/NO2I 250, MAP swath increases with view angle, oversampling keeps pixel resolution ~1 km; CLIM 465	11 d	11:30D	CO2I & NO2I 405–490 nm @ 0.6 nm resolution, 747–773 nm @0.12 nm, 1590–1675 nm @0.30 nm, 1990–2095 nm w/0.35 nm resolution at 0.8 km GSD. MAP 410, 443, 490, 555, 670, 753, 865 nm scanned along track fore, aft, ±60°, 12 x-track detectors, ~1km GSD. CLIM 670, 753 nm @ 174 m GSD, 1370 nm @ 348 m GSD
**Copernicus Sentinel Expansion Mission Copernicus Hyperspectral Imaging Mission for the Environment (CHIME)**, A, B	A 2029-2034, B 2031–2037	Polar sun synchronous	786	290	25 d, together 12.5 d	10:45D	~210 bands 400–2500 nm @ ≤10 nm; 20–30 m GSD band dependent.
**Copernicus Expansion mission Land Surface Temperature Monitoring (LSTM),** 1A, 1B	A 2029–2034, B 2031–2038	Polar sun synchronous	640	600–700 km	4 d with 1 instrument; 2 d with 2 instruments 1 d with 4 instruments	12:30A	Primary mission 11 bands, VSWIR 490, 665, 865, 945, 1380, 1610 nm @ 30 m and 8.6, 8.9, 9.2, 10.9, 12.0 μm. Secondary mission 13 bands, TIR 8.2, 9.1, 8.63, 12.63, 7.5, 12.2, 9.0, 9.8, 10.5, 10.95, 12.3, 9.3, 9.53 μm, all @ 30–50 m GSD, band dependent.

**Table 22:** Rose-L is the fourth of the missions and will fly in a polar orbit. The fifth (CIMR) will be in a quasi-polar LEO orbit at a constant incidence angle of 55.5°, and the last one (CRISTAL) will be placed into an inclined orbit. The CIMR (2029) will measure sea-surface salinity, sea-surface temperature, and ice in polar regions. And the last mission is CRISTAL (A, B) to measure polar ice and snow topography. All three are in formulation phases, so less can be described about them. Sources include instrument sites, OSCAR, Copernicus Expansion missions, and EO Portal.

Satellite/ Instrument	Launch Date	Orbit Type	Altitude km	Swath, km	Repeat Frequency Days	Equatorial crossing Time	Radar/Microwave Bands, Wavelengths, Frequencies
**Copernicus Expansion Mission Observation System for Europe L-band (ROSE-L) A, B** **Complements C-Band Sentinel-1**	A: 2030– 2036 B: 2931– 2038	Polar LEO sun-synchro- nous	693	80–400 km mode dependent	days-weeks, mode dependent	18:00A 06:00D	L-band SAR (1.2575 GHz) polaremetric and interferometric, side looking 15–45° off Nadir, best resolution is 5 m
**Copernicus Expansion Mission Copernicus Imaging Microwave Radiometer (CIMR); A, B fly in loose formation with METOP-SG-B**	A 2029- 2036, B 2031– 2036	Quasi-polar, circular and sun synchronous at constant incidence angle of 55.5° around the poles	830	1900	sub-daily “no hole” polar cover; 95% global coverage daily	dawn /dusk circular orbit; 06:00D	Conical scanning w/8 m reflector antenna, 5-band multifrequency microwave radiometer; Dual polarization V and H, in Ka (36.5), Ku (18.7), C (6.875), X (10.65), L (1.4135) GHz. GSD for Ka, Ku 2.5 km, for C, X 7.5 km, and L 30 km pixel resolution.
**Copernicus Expansion Mission Copernicus polaR Ice and Snow Topography ALtimeter-CRISTAL A, B**	A 2028– 2034, B 2031– 2036	drifting 92° polar orbit, will not measure beyond 81.5° N & S	717	NA	IRIS 367 d, AMR-CR monthly at 30 km or 10 d at 100 km	various	IRIS Dual frequency SAR altimeter Ku 13.5, Ka 35.75GHz @ 10 km GSD in SAR mode and 80 km in along-track mode. Microwave Radiometer (AMR-CR) for atmospheric corrections and surface type characterization with 3 bands: Ka 35.75, Ku 18.7, 34 GHz @ 25 km GSD.

**Table 23:** New satellites in the next decade.

Satellite /Instrument	Launch Date	Orbit Type	Altitude, km	Swath, km	Repeat Frequency Days	Equatorial crossing Time	UV/VNIR/SWIR, 380–2500 nm, GSD in m	MID IR 3.0–6.0 µm, TIR 6.0–14.0 μm. Various GSD	Radar and Microwave Bands. Wavelengths, Frequencies, GSD Various
**JAXA GOSAT-GW**	2024–2031	Polar, LEO, sun synchronous	666	AMSRE3 = 1450, TANSO-3 selectable 911 for wide or 90 for focus	3d	13:30 A	TANSO-3, Bands, band1,~450 nm, <0.5 nm (NO_2_), band 2 ~760 nm <0.05 nm Band 3 ~1600 nm <0.2 nm spectra resolution, for CO_2_ and CH_4_	--	AMSRE3 11 frequencies 6.9–183 GHz with 21 channels, GSD changes with frequency.
**Canadian WildFireSat, Pre Phaes A planning**	around 2030, 5 years	Polar or GEO, or 2 sats.	n/a	n/a	daily or subdaily	18:00 ± 2 h	Multispectral VNIR, possibly like Landsat-8 or Sentinel 2	Uncooled IR Bolometer technology. Possible MWIR 3.1–4.8 μm ~400 m GSD, Likely 2 IR micro-bolometers 10–12 μm range, @400 m GSD	**--**
**4th Generation Himawari-10 GEO satellite**	2028–2038	GEO @ 140.7° E	35,786	Full disk of Earth, Continental l, Targeted	seconds, minutes to hourly depending on mode	Full disk = 10 min, regional 1000 km × 1000 km every 2.5 min (up to 4 specified). High res areas 1000 km × 500 km every 30 s	GHMI: 470 (<1 km), 550 (<1), 660 (<0.5), 860 (<1), 1380 (<2), 1610 (<1) in km. 2255 (<1 km) in nm	GHMI: 3.85 (<1 km), 5.15 (<km), 6.25 (<2 km 6.95 (<2 km) μm. 7.35, 8.60, 9.625, 10.4, 11.2, 12.40, 13.3μm (all <2 km). GHMS 1689–2250 cm^−1^, 680–109 cm^−1^	**--**
**GOES-XO GOES EAST,** **WEST, and Continental**	XO-East 2032–2047, XO- West 2035–2050, XO- Central 2035–2050.	GEOS East/West will have improved imager, hyperspectral IR sounder for ocean color and atmospheric condition. GOES Central will have a sounder and an atmospheric composition instrument.	35,786	Full disk of Earth, U.S. CONUS, targeted	full disk and U.S. CONUS better than 15 min and 5 min sampling.	Seconds to hourly, depending on mode	GXI Imager: 18 channels, improve ABI with 1 new band (910 nm) for WV in lower troposphere (daylight), improved day/night band, 3.9 μm @at 1km and 640 nm @ 250 m GSD	**--**	improve ABI with 1 new band (5.15 μm) for WV in lower troposphere, near the ground
**Landsat-Next (L-10), w/3** **Platforms, LandIS** **instrument**	2030–2038	Polar, LEO sun synchronous	653	164	16 (6 d w/3 together)	10:00	LandIS (bandwidth, nm) 5 bands: 490 (65), 560 (35), 665 (30), 842 (115), 1610 (90) nm, @ 12 m GSD, 10 bands 443 (20), 600 (30), 620 (20), 650 (35), 705 (15), 740 (15), 865 (20), 985 (20), 1035 (20), 1090 (20), 2038 (25), 2198 (20) @ 20 m GSD. Cal bands 412, 945, 1375 nm @ 60 m GSD	--	LandIS (bandwidths, nm) 5 bands 8300 (250), 8600 (350), 9100 (350), 11,300 (550), 12,000 (550) nm @60 m GSD

**Table 24:** Comparison of spectral bands of Landsat-8 and -9 with Landsat Next. On the left side of the table are the bands for Landsat-8 and Landsat-9, and on the right side, those planned for Landsat Next (L-10). The number of bands increases from 11 to 26 and pixel size decreases from 30 m to 10 m, 20 m, or 30 m, depending on band wavelengths. Redrawn from websites cited above.

L-8/L-9	https://www.usgs.gov/faqs/what-are-band-designations-landsat-satellites						L10	https://landsat.gsfc.nasa.gov/satellites/					
Band	λ Region	Purpose	GSD	λ, nm	λ Center	Band Width	Band	λ Region	Purpose	GSD	λ, nm	λ Center	Band Width
							1	Violet	Improve aerosol retrieval & CDOM inland & coastal water	60	402–422	412	20
1	Blue 1	Coastal/Aerosols	30	430–450	440	20	2	Blue	Landsat Heritage Coastal/Aaerosols	20	433–453	443	20
2	Blue2	water	30	450–510	480	60	3	Blue	Landsat Heritage	10	457.5–522.5	490	65
3	Green	Carotenoids	30	530–590	560	60	4	Green	Landsat Heritage	10	542.5–577.5	560	35
8	Panchromatic	pan shaprening	15	500–680	590	180							
							5	Yellow	Chlorosis, Plant stress	20	590–610	600	20
							6	Orange	Phycocyanin detection, Harmful algal blooms (HABs)	20	610–630	620	20
							7	Red 1	Phycocyanin, chlorophyll	20	640–660	650	20
4	Red	Chlorophyll	30	640–670	655	30	8	Red 2	Landsat Heritage Chlorophyll	10	650–680	665	30
							9	Red Edge 1	LAI, Chlorophyll, plant stress (Sentinel-2 (S-2))	20	697.5–712.5	705	15
							10	Red Edge 2	LAI, Chlorophyll, plant stress (S-2)	20	732.5–747.5	740	15
							11	NIR Broad	10 NDVI (S-2)	10	789.5–894.5	842	115
5	Near-infrared (NIR)	NIR plateau	30	850–880	865	30	12	NIR 1	Landsat Heritage	20	855–875	865	20
							13	Water Vapor	Atmospheric corrections, Land Surface Temperature, Surface Reflectance (S-2)	60	935–955	945	20
							14	Liquid Water	Liquid water	20	975–995	985	20
							15	Snow/Ice 1	Snow grain size for water resources	20	1025–1045	1035	20
							16	Snow/Ice 2	Ice absorption,	20	1080–1100	1090	20
									snow grain size				
9	NIR	Cirrus	30	1360–1380	1370	20	17	NIR	Landsat Heritage Cirrus	60	1360–1390	1375	30
6	Shortwave Infrared (SWIR) 1	Water absorption	30	1570–1650	1610	80	18	SWIR 1	Landsat Heritage	10	1555–1655	1610	90
							19	SWIR 2a	Landsat Heritage subdivided for Cellulose/crop residue	20	2025.5–2050.5	2038	25
							20	SWIR 2b	Landsat Heritage subdivided for Cellulose/crop residue	20	2088–2128	2108	40
7	Shortwave Infrared (SWIR) 2	separation minerals, cellulose/crop residue	30	2110–2290	2200	180	21	SWIR 2c	Landsat Heritage subdivided for Cellulose/crop residue	20	2191–2231	2211	40
							22	TIR 1	Mineral surface composition (ASTER)	60	8175–8425	8300	250
							23	TIR 2	Emissivity separation, volcanoes (SO2) (MODIS/ASTER)	60	8475–8725	8600	250
							24	TIR 3	Mineral surface composition (ASTER)	60	8925–9275	9100	350
10	Thermal infrared TIRS 1	Radiance, Temperature	100	10600–11,190	10,895	590	25	TIR 4	Landsat Heritage Surface temperature, carbonates	60	11,025–11,575	11300	550
